# Phosphorylation by CaMKIV regulates the transcriptional repressor activity and half-life of REST/NRSF

**DOI:** 10.1126/sciadv.aec0781

**Published:** 2026-10-16

**Authors:** Hanako Tsushima Semini, Beatrice Corradi, Caterina Canevari, Dmytro Shmal, Martina Chiacchiaretta, Nara Liessi, Andrea Di Fonzo, Emanuele Carminati, Andrea Armirotti, Anna Fassio, Luca Maragliano, Anna Rocchi, Fabio Benfenati

**Affiliations:** ^1^ Center for Synaptic Neuroscience and Technology, Istituto Italiano di Tecnologia, Largo Rosanna Benzi, 10, 16132 Genova, Italy.; ^2^ Department of Experimental Medicine, Università degli Studi di Genova, Viale Benedetto XV 3, 16132 Genova, Italy.; ^3^ Analytical Chemistry Facility, Istituto Italiano di Tecnologia, 16163 Genoa, Italy.; ^4^ IRCCS Azienda Ospedaliera Metropolitana, Largo Rosanna Benzi 10, 16132 Genova, Italy.; ^5^ Department of Life and Environmental Sciences, Polytechnic University of Marche, Via Brecce Bianche, 60131 Ancona, Italy.

## Abstract

Repressor Element-1 (RE1)–Silencing Transcription Factor (REST) homeostatically regulates neural activity by repressing a large cluster of neural genes. However, whether REST can be regulated by calcium(2+) (Ca^2+^)–activated kinases is unknown. We investigated Ca^2+^/calmodulin-dependent protein kinases (CaMKs) as upstream regulators of REST fate and activity. We show that REST is phosphorylated by CaMKIV at Ser^322^ site in the fifth-to-sixth Zn-finger domain linker. A phosphomimic mutant at Ser^322^ decreased REST repressor activity and caused its transition to the cytosol, followed by degradation. Molecular dynamics simulations of the phosphomimic REST mutant revealed a sharp decrease in the stability of the REST-RE1 binding interface. Phosphorylation of REST by CaMKIV suppressed the downscaling of excitatory postsynaptic current (EPSC) frequency in response to hyperactivity. Moreover, the homeostatic effects of CaMKIV on EPSC amplitude were inhibited in the absence of REST. The results demonstrate that CaMKIV phosphorylation plays a crucial role in regulating REST levels and repressor activity and that an interplay exists between CaMKIV and REST in homeostatic plasticity mechanisms.

## INTRODUCTION

REST [Repressor Element-1 (RE1)–Silencing Transcription Factor], also known as NRSF (Neuron-Restrictive Silencer Factor), is a transcriptional repressor that plays a key role in gene regulation (*1*, *2*). It contains nine Zn-finger (ZNF) domains, through which it binds to a specific DNA sequence of 17 to 21 nucleotides named RE1 (*3*, *4*), where it recruits cofactors [such as Co-REST, mSin3, and histone deacetylases (HDACs)] to modify chromatin structure and repress the transcription of the downstream gene. RE1 elements have been identified in more than 1800 genes in the human and mouse genomes, with variable copy number and affinity for REST (*1*). Many of the REST target genes are neuronal, while others are involved in cell cycle regulation and cytoskeleton assembly. In nonneuronal tissues, REST is active and suppresses neuronal gene expression, while during neuronal differentiation, REST is down-regulated, allowing neuronal genes to be expressed [(*5*–*8*); see (*9*) for review].

In addition to its role in neuronal differentiation, residual REST expression is preserved in mature neurons, albeit at low levels, and plays postdevelopmental roles in the brain. In neurons, REST is an essential actor of homeostatic plasticity responses. It is promptly induced by hyperactivity and migrates into the nucleus, where it inhibits the transcription of genes governing intrinsic excitability and synaptic transmission (*9*, *10*). Under conditions of neuronal hyperactivity, REST induction inhibits the expression of voltage-gated Na^+^ channels (*11*), blunts excitatory synaptic transmission at the presynaptic level (*12*), and enhances inhibitory synaptic transmission onto excitatory neurons (*13*). REST is also involved in controlling changes in neuronal networks in response to early-life adversity, promoting resilience (*14*–*16*). In addition, REST was identified as playing a key role in regulating life span and stress resistance during aging by repressing hyperactivity (*17*, *18*) and by preventing senescence through a regulation of the autophagic flux (*19*). A homeostatic effect against hyperactivity was also observed in astrocytes, where REST increases glutamate uptake and potassium clearance from the synaptic environment (*20*). Beyond its canonical role in neurodevelopment and its pivotal role in regulating brain homeostasis, REST dysregulation has been implicated in a variety of pathological contexts, including epilepsy, neuroinflammation, brain ischemia, pain, and Alzheimer’s and Huntington’s diseases, where it may contribute to neuronal vulnerability or adaptive responses [(*17*, *19*, *21*–*27*); see (*28*) for review].

REST shuttles between the nucleus, where it associates with a variety of interactors and actuates transcriptional repression, and the cytoplasm, where it remains inactive. Several regulators of REST favor its retention in the nucleus, such as the cofactors mSin3, Co-REST, and RILP (*2*, *29*), and the optogenetic disruption of these interactions inhibits REST activity in the nucleus (*30*). Although no full-length experimental structure is available for REST, domain mapping suggests that it is a modular protein comprising N- and C-terminal repression domains and a central ZNF DNA binding region. The latter adopts the canonical C2H2 architecture, consisting of nine conserved Cys^2^-His^2^ motifs arranged in tandem, each coordinating a Zn^2+^ ion and mediating DNA binding (*31*–*33*). Structural modeling and sequence alignment suggest that REST’s ZNF domains adopt the classical ββ-α fold, namely, a β-hairpin followed by an α helix, stabilized by Zn^2+^ coordination (*34*). Each ZNF module typically interacts with a trinucleotide segment of DNA, allowing REST to contact an extended nucleotide sequence and achieve high specificity (*35*).

REST activity is tightly regulated, not only at the transcriptional and epigenetic levels but also through posttranslational modifications, among which phosphorylation plays a central role. Phosphorylation has been shown to regulate REST half-life and turnover. Casein kinase 1 (CK1) was found to associate and phosphorylate REST at four serine residues (Ser^1013^, Ser^1024^, Ser^1027^, and Ser^1030^) within two degron motifs in the C-terminal region, enabling recognition by the E3 ubiquitin ligase β-TrCP and targeting it for proteasomal degradation (*36*–*38*). Moreover, extracellular signal–regulated kinases 1 and 2 (ERK1/2) were found to phosphorylate the proline-directed phosphorylation motifs in REST. ERK1/2 phosphorylation promotes rapid REST degradation, relieving repression of neuronal gene expression, while their dephosphorylation by the C-terminal domain small phosphatase 1 increases REST stability and half-life (*39*). This ERK-mediated regulation plays an important role during neurogenesis, in which ERK activation in response to growth factors down-regulates REST, allowing neuronal differentiation.

Despite these insights, the full spectrum of REST phosphorylation sites and their functional consequences remain incompletely understood. While phosphorylation has been predominantly viewed as a mechanism to down-regulate REST in neural progenitors and differentiating neurons, it could also help regulate downstream effects of REST overexpression during hyperactivity in mature neurons by attenuating REST-mediated transcriptional repression and restoring REST levels to baseline. As calcium entry and calmodulin (CaM) activation are the most likely signals that trigger homeostatic plasticity and REST overexpression (*40*, *41*), we investigated whether REST can also be phosphorylated by Ca^2+^/CaM-dependent kinases (CaMKs) and whether this phosphorylation affects its repressor activity, nuclear/cytosolic shuttling, and half-life.

We have found that, consistent with the presence of consensus phosphorylation sites for CaMKs, REST is phosphorylated by CaMKIV in primary neurons on a main site intercalated between the fifth and sixth ZNF domains; and its phosphorylation causes dissociation from the DNA RE1 motif, inhibition of its transcriptional repressor activity, and escape from the nucleus to the cytoplasm, where it undergoes degradation, likely mediated by Cathepsin B. Phosphorylation of REST by CaMKIV counteracts the downscaling of excitatory postsynaptic current (EPSC) frequency under basal conditions and in response to hyperactivity. Moreover, the homeostatic effects of CaMKIV on the EPSC amplitude were inhibited in the absence of REST, suggesting that REST is a downstream effector of CaMKIV. The results demonstrate that CaMKIV phosphorylation plays a crucial role in the homeostatic regulation of REST levels and repressor activity and that an interplay exists between CaMKIV and REST in homeostatic plasticity mechanisms.

## RESULTS

### CaMKs decrease the expression and transcriptional repressor activity of REST in human embryonic kidney 293T cells and primary neurons

Recent evidence suggests that posttranslational modifications, including phosphorylation and ubiquitination, influence the stability and turnover of the REST protein. ERK1/2 and CK1 kinases phosphorylate REST and promote its degradation by the ubiquitin-proteasome system (*36*, *38*, *39*). Given that REST is up-regulated in mature neurons by hyperactivity, a condition that promotes Ca^2+^ entry, we tested whether CaMK-mediated phosphorylation regulates REST turnover and fate in neurons. We first evaluated whether Ca^2+^/CaM signaling interferes with REST stability. When primary cortical neurons were incubated with the CaM inhibitor *N*-(6-aminohexyl)-5-chloro-1-naphthalenesulfonamide hydrochloride (W7), an increase in REST protein was observed (Fig. 1A, left). Because CaM activates various kinases and phosphatases, we asked which downstream effector could be involved. Primary cortical neurons were treated with either the calcineurin inhibitor cyclosporin A (CsA) or with the competitive CaMK blocker *N*-{-[*N*-(4-chlorocinnamyl)-*N*-methylaminomethyl]phenyl}-*N*-(2-hydroxyethyl)-4-methoxybenzene sulfonamide (KN93). While inhibition of calcineurin did not affect REST protein levels (Fig. 1A, middle), KN93, at concentrations inhibiting all CaMKs, induced a significant increase in REST protein levels that was completely absent when its inactive analog 2-[*N*-(4-methoxybenzenesulfonyl)]amino-*N*-(4-chlorocinnamyl)-*N*-methylbenzylamine phosphate (KN92) was used (Fig. 1A, right). None of the treatments was associated with changes in REST mRNA levels, as shown by reverse transcription quantitative polymerase chain reaction (RT-qPCR) analysis (fig. S1). The same treatments applied to primary cortical astrocytes did not alter REST protein or mRNA levels (fig. S2), indicating that the effects of CaMK inhibition were specific to neurons.

**Fig. 1.**
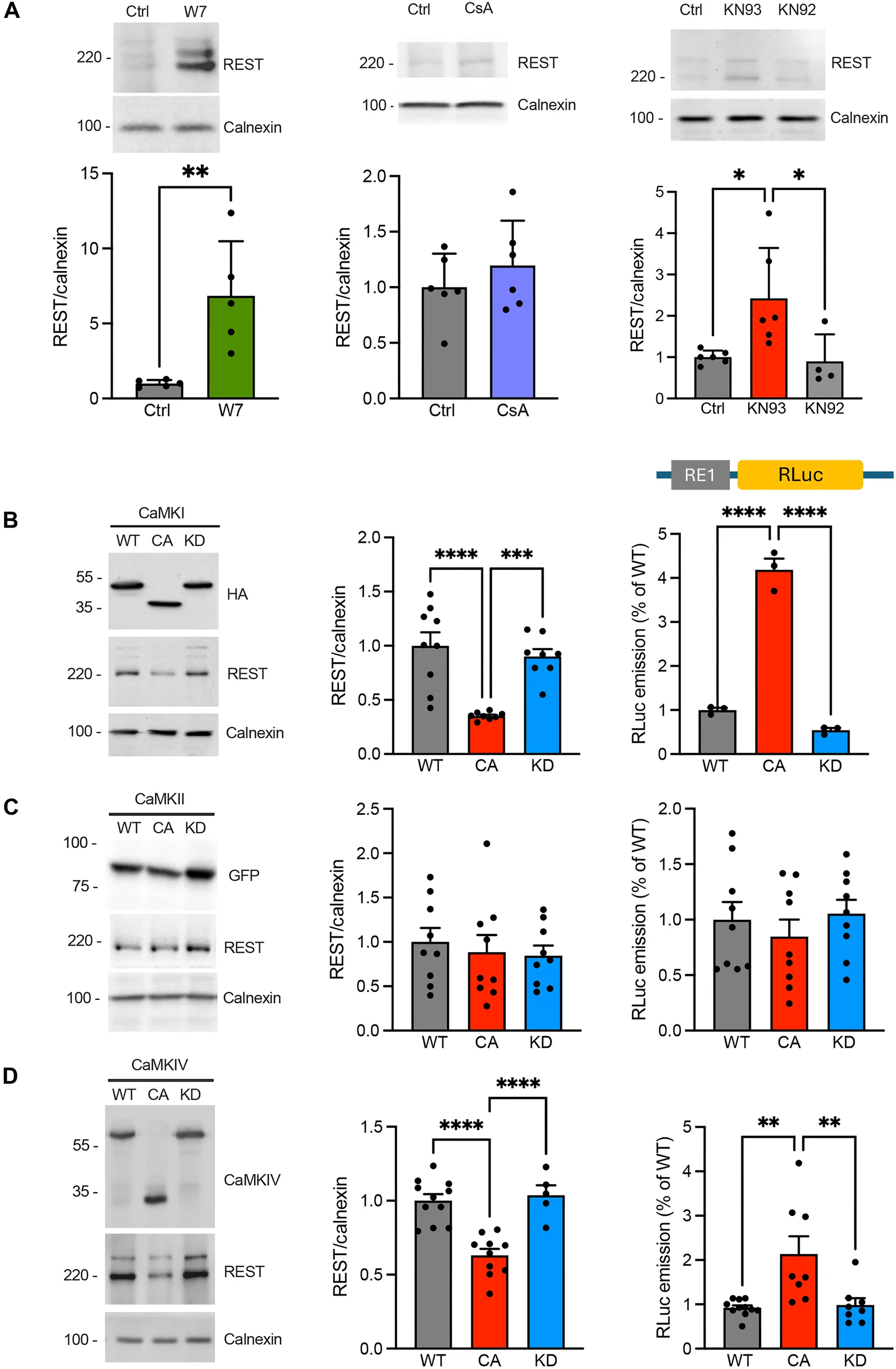
Inhibition of Ca^2+^-CaM–dependent activities increases the endogenous levels of REST protein in primary mouse cortical neurons and HEK293T cells. (**A**) Left: Representative immunoblots and mean ± SEM REST protein in primary neurons [days in vitro 14 (DIV14)] treated with W7 (20 μM). **P = 0.0071; unpaired Student’s *t* test (*n* = 5 independent preparations). Middle: Representative immunoblots and mean ± SEM REST protein in primary neurons (DIV14) treated with CsA (1 μM). *P* = 0.36; unpaired Student’s *t* test (*n* = 5 independent preparations). Right: Representative immunoblots and mean ± SEM REST protein in primary neurons (DIV14) treated with vehicle (Ctrl), the KN93 (10 μM), or KN92 (10 μM). *P = 0.031, Ctrl versus KN93; *P = 0.032, KN93 versus KN92; one-way analysis of variance (ANOVA)/Holm-Šídák’s tests (*n* = 5 independent preparations). Calnexin was used as a loading control. (**B** to **D**) Left: Representative immunoblots of the expression of wild-type (WT), constitutively active (CA), and kinase-dead (KD) CaMKs, and REST in HEK293T cells 48 hours after transfection. The expression of CaMKI (B), CaMKII (C), and CaMKIV (D) was followed using antibodies to hemagglutinin (HA) tag, green fluorescent protein (GFP) tag, and CaMKIV, respectively. Middle: Mean ± SEM REST levels shown as REST/calnexin immunoreactivity ratios. Right: A RE1-Luciferase construct, with *Renilla* Luciferase (*RLuc*) gene cloned downstream of the RE1 sequence, was used to detect the repressor activity of endogenous REST in HEK293T cells, transfected with the three variants of CaMKI (B), CaMKII (C), and CaMKIV (D). The *RLuc* chemiluminescence ratio is shown as mean ± SEM. (B) REST/calnexin: ***P = 0.0007, CA versus KD; ****P < 0.0001, CA versus WT or KD. *RLuc*: ****P < 0.0001, CA versus WT or KD. (C) REST/calnexin: *P* = 0.86, CA versus WT; *P* = 0.98, CA versus KD. *RLuc*: *P* = 0.74, CA versus WT; *P* = 0.58, CA versus KD. (D) REST/calnexin: ****P < 0.0001, CA versus WT or KD; *RLuc*: **P = 0.0018, CA versus WT; **P = 0.0057, CA versus KD. One-way ANOVA/Tukey’s tests (*n* = 8 to 9 independent preparations).

KN93 is a broad-spectrum CaMK inhibitor with multiple molecular targets; thus, the results do not identify the specific CaMKs that mediate the effects on REST protein levels. To address this point, we overexpressed CaMKI, CaMKII, and CaMKIV as the wild-type (WT) form or as constitutively active (CA) and kinase-dead (KD) variants in human embryonic kidney (HEK) 293T cells that constitutively express REST. Under these experimental conditions, we evaluated (i) the changes in the expression levels of the REST protein by immunoblotting as a ratio between REST and calnexin immunoreactivities and (ii) the effects on the REST repressor activity using a gene reporter assay in which *Renilla* luciferase (*RLuc*) is driven by the SV40 promoter fused to a triple RE1*cis* site from the promoter region of the voltage-gated Na^+^ channel 1.2 (*SCN2A*) gene, a well-known REST target [Fig. 1, B to D, and (*30*); see Materials and Methods].

Western blotting analysis revealed that the CA forms of both CaMKI and CaMKIV significantly decreased REST levels, while the CA form of CaMKII was virtually ineffective (Fig. 1, B to D, left and middle). No effects with respect to the WT forms of the kinases were observed with the respective KD variants, suggesting that the endogenous activation of these kinases is not functional in this cell line under these conditions. In agreement with the observed decrease in REST levels, we observed a markedly increased luciferase signal in HEK293T cells transfected with the CA forms of both CaMKI and CaMKIV and no effect with the active form of CaMKII (Fig. 1, B to D, right), indicating a corresponding drop in the REST repressor activity at RE1 sites. The data indicate that CaMKI and CaMKIV are potentially implicated in the regulation of REST protein stability, at least in HEK293T cells.

### Knockdown of CaMKIV but not of CaMKI in primary neurons recapitulates the effects of CaMK inhibition on REST protein levels

To gain insights into the mechanism underlying CaMKI/CaMKIV-mediated regulation of REST levels in neurons, we analyzed REST protein levels and activity after silencing either CaMKI or CaMKIV by RNA interference using lentiviral vectors (Fig. 2 and fig. S3). While we used a very effective, previously reported short hairpin RNA (shRNA) for CaMKI (*42*), we tested two distinct shRNAs (sh#1 and sh#2) for their efficiency in knocking down CaMKIV (fig. S3A). Both shRNAs effectively down-regulated CaMKIV in primary neurons, resulting in >60% decreases in CaMKIV levels. We selected sh#2 for subsequent experiments and found that shRNA targeting of CaMKIV was highly selective and did not alter the mRNA levels of either *CaMKI* or *CaMKII* (fig. S3, B and C).

**Fig. 2.**
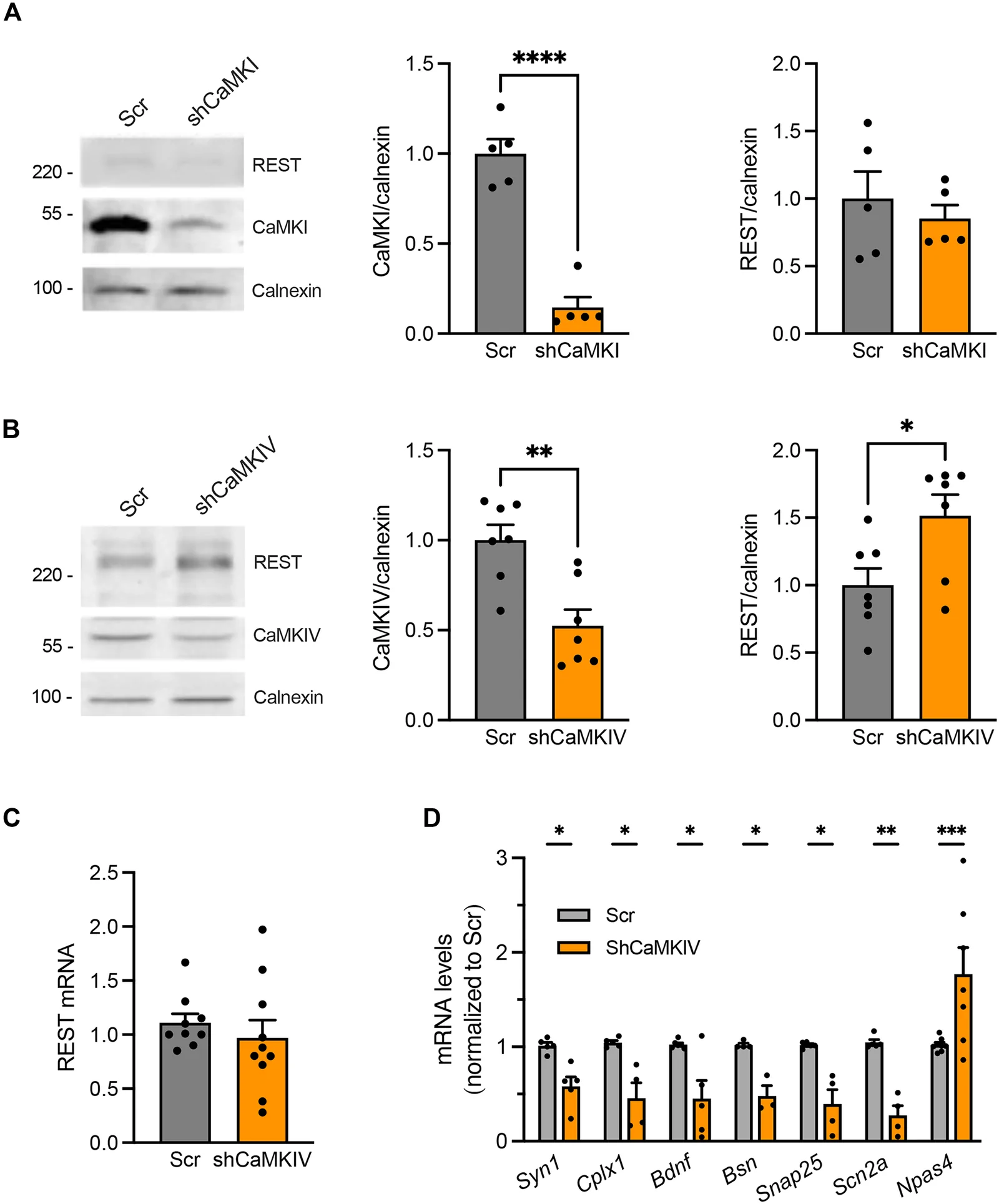
Down-regulation of CaMKIV but not of CaMKI increases the endogenous levels of REST in mouse primary cortical neurons. (**A**) Primary mouse cortical neurons were transduced at DIV7 with lentiviruses encoding shRNAs to down-regulate CaMKIα (shCaMKI) or a scrambled version thereof (Scr). Left: Representative immunoblots monitoring the expression of endogenous CaMKIα, REST, and calnexin in the neuronal preparations at DIV14. Middle and right: Mean (±SEM) CaMKIα (middle) and REST (right) expression shown as ratios with calnexin immunoreactivity. CaMKIα/calnexin: ****P < 0.0001; REST/calnexin: *P* = 0.53. Unpaired Student’s *t* test (*n* = 5 independent preparations). (**B**) Primary mouse cortical neurons were transduced at DIV7 with lentiviruses encoding shRNAs to knock down CaMKIV (shCaMKIV) or a scrambled version thereof (Scr). Left: Representative immunoblots monitoring the expression of endogenous CaMKIV, REST, and calnexin in the neuronal preparations at DIV14. Middle and right: Mean (±SEM) CaMKIV (middle) and REST (right) expression, shown as ratio with calnexin immunoreactivity. CaMKIV/calnexin: **P = 0.0024; REST/calnexin: *P = 0.025. Unpaired Student’s *t* test (*n* = 7 independent preparations). (**C**) Mean (±SEM) REST mRNA levels, assessed by real-time qPCR in primary cortical neurons treated with either Scr or shCaMKIV. *P* = 0.46; unpaired Student’s *t* test (*n* = 6 independent experiments). (**D**) Real-time qPCR of the REST target genes synapsin I (*Syn1*), complexin-1 (*Cplx1*), brain-derived neurotrophic factor (*Bdnf*), Bassoon (*Bsn*), synaptosomal-associated protein 25 (*Snap25*), voltage-gated Na^+^ channel 1.2 (*Scn2a*), and neuronal PAS domain protein 4 (*Npas4*) in primary cortical neurons treated with either Scr or shCaMKIV. *Syn1*, *P = 0.0477; *Cplx1*, *P = 0.0175; *Bdnf*, *P = 0.0150; *Bsn*, *P = 0.0477; *Snap25*, *P = 0.0150; *Scn2a*, **P = 0.0023; *Npas4*, ***P = 0.0001. Two-way repeated-measures ANOVA/Holm-Šídák’s tests (*n* = 5 to 6 independent experiments).

To ascertain whether both CaMKI and CaMKIV are active in decreasing REST levels in neurons, we silenced either kinase by transducing neurons with lentivirus encoding the respective shRNA. Western blotting analysis revealed that silencing CaMKIV, but not CaMKI, reproduced the observed increase in REST protein levels (Fig. 2, A and B) in the absence of changes in REST mRNA levels (Fig. 2C). Consistent with the elevated REST levels, qPCR performed on the same neuronal preparations showed significant decreases in mRNA levels of a cohort of REST target genes, such as those encoding synapsin I (*Syn1*), complexin-1 (*Cplx1*), brain-derived neurotrophic factor (*Bdnf*), Bassoon (*Bsn*), synaptosomal-associated protein 25 (*Snap25*), and Na_V_1.2, (*Scn2a*) while, as previously reported (*43*), the expression of the neuronal PAS domain protein 4 (*Npas4*) gene was increased (Fig. 2D).

In conclusion, the data suggest that CaMKIV is responsible for the posttranscriptional modulation of REST protein levels in primary neurons, consistent with the preliminary results obtained with CaMK inhibitors. Notably, while CaMKI is expressed by both neurons and astrocytes, CaMKII and CaMKIV are virtually neuron specific (fig. S4), indirectly confirming the role of CaMKIV in regulating REST levels, observed in neurons but not in astrocytes under the same experimental conditions. Moreover, the distinct effects of CaMKI and CaMKIV in neurons may depend on their subcellular distribution. Whereas CaMKIV is predominantly nuclear and therefore positioned to directly interact with nuclear REST, CaMKI is largely cytosolic, which limits its ability to efficiently target REST in neurons, despite its effectiveness in heterologous systems such as HEK293T cells.

### REST degradation is triggered by CaMKIV phosphorylation

Phosphorylation by CK1 and ERK1/2 has been shown to target REST to the ubiquitin-proteasome degradation. Thus, we sought to investigate whether phosphorylation by CaMKIV, which also decreased REST protein levels, was associated with a similar mechanism. We considered the canonical degradative pathways: the proteasomal and the lysosomal degradation (Fig. 3A).

**Fig. 3.**
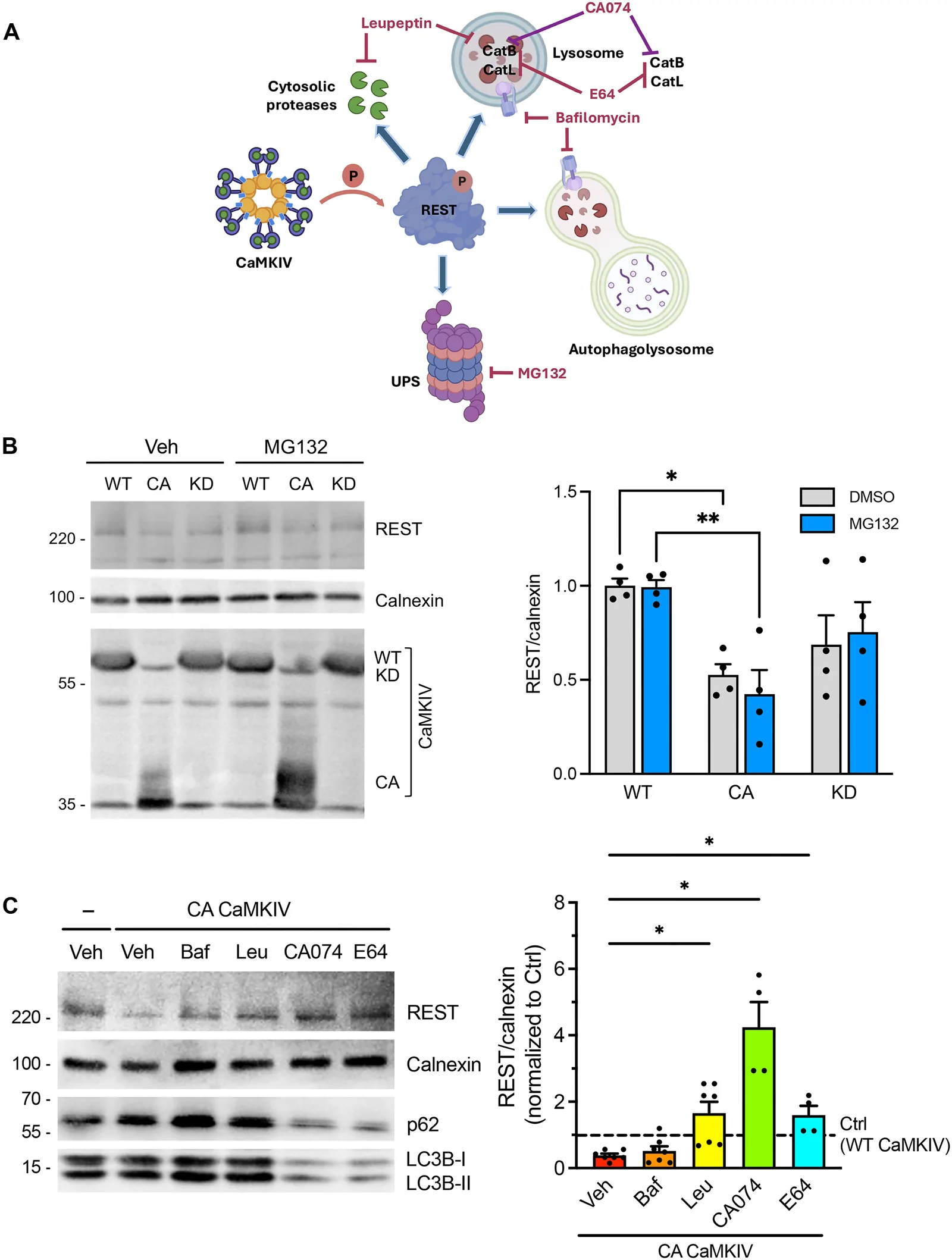
CaMKIV phosphorylation of REST triggers its lysosomal degradation. (**A**) Schematics of the four possible degradation pathways that CaMKIV-phosphorylated REST can undertake and the three inhibitors that were used to dissect the fate of REST. Cat, cathepsin. (**B**) HEK293T cells were transfected with WT, CA, or KD CaMKIV and treated with cycloheximide (CHX) to in4hibit further translation. Then, cells were treated for 16 hours with either vehicle [dimethyl sulfoxide (DMSO)] or MG-132 (5 μM), an inhibitor of the ubiquitin-proteasome system (UPS). Left: Representative immunoblots monitoring the expression of WT, CA, and KD CaMKs, REST, and calnexin, respectively, in HEK293T cells, 48 hours after transfection with the kinase constructs. Right: Mean (±SEM) REST protein levels, shown as ratios with calnexin immunoreactivity under the various experimental conditions. No effects of inhibition of the UPS were observed. *P = 0.017, CA versus WT in DMSO; **P = 0.0048, CA versus WT in MG-132. Two-way ANOVA/Tukey’s tests (*n* = 4 independent preparations). (**C**) HEK293T cells were transfected with either WT or CA CaMKIV and treated with CHX to inhibit further translation. Successively, cells were treated for 16 hours with DMSO (Veh), bafilomycin A1 (Baf; 100 nM; an inhibitor of lysosomal acidification and autophagy), leupeptin (Leu; 40 μM; an inhibitor of the lysosomal and cytosolic proteases), CA074 (a specific inhibitor of Cathepsin B), or E64 (an inhibitor of cathepsins). Left: Representative immunoblots monitoring the expression of REST, calnexin, and the autophagy markers p62 and LC3B-I and -II. Right: Mean (±SEM) REST/calnexin ratios normalized with respect to the control REST/calnexin ratio in WT CaMKIV–expressing cells. Leu, *P = 0.0351; CA074, *P = 0.0361; E64, *P = 0.0481. Mixed-effects ANOVA/Dunnett’s tests (*n* = 4 independent preparations).

To this aim, we transfected HEK293T cells with WT, CA, or KD CaMKIV and treated them with cycloheximide (CHX) to block REST neosynthesis. Then, we treated the cells with either vehicle or 5 mM MG-132, a specific inhibitor of the ubiquitin-proteasome system. As shown in Fig. 1, only cells expressing CA CaMKIV exhibited significantly decreased REST levels. However, this down-regulation of REST protein was not attributable to proteasome-mediated degradation, as MG-132 was ineffective at antagonizing the decreased REST levels observed with CA kinase expression (Fig. 3B). The lack of MG-132 effects in cells expressing WT or KD CaMKIV also indicates that, in the absence of specific activation of signal transduction pathways, REST is not constitutively targeted to the proteasome. Next, we addressed the possibility that REST is degraded by alternative pathways, such as lysosomal degradation. We transfected HEK293T cells with either WT or CA CaMKIV and blocked REST neosynthesis (see above) before treating the cells with vehicle, 100 nM bafilomycin A1 (inhibitor of v–adenosine triphosphatase responsible for lysosomal acidification), 40 μM leupeptin (inhibitor of cysteine proteases), 10 μM CA074 (specific cathepsin B inhibitor), or 10 μM E64 (inhibitor of cathepsins). The results show that CA074 potently reversed the degradation of REST induced by CA CaMKIV, increasing its overall levels, whereas leupeptin and E64 restored REST levels to control levels (Fig. 3C). In contrast, bafilomycin A1 was completely ineffective, indicating that REST degradation does not require acidic pH. The differential effect of bafilomycin A1, which additionally blocks autophagy progression, is demonstrated by the increase in the autophagy markers LC3-II and p62 observed after bafilomycin A1 treatment but not after leupeptin, CA071, or E64 treatment (fig. S5). Together, the data suggest that REST degradation is likely mediated by Cathepsin B and does not require acidic pH.

### REST is phosphorylated by CaMKIV on serine-322 between the fifth and the sixth ZNF domain, and its phosphomimic mutant impairs the transcriptional repressor activity and half-life

CaMKIV, together with CaMKI and CaMKII, is defined as a “multifunctional CaM kinase” for its ability to phosphorylate a wide range of substrates (*44*, *45*). CaMKIV needs to be activated in the cytoplasm by CaMK kinase (CaMKK) before entering the nucleus, where it acts as a potent activator of Ca^2+^-dependent transcriptional effects through phosphorylation of downstream transcriptional effectors, such as adenosine 3′,5′-monophosphate response element–binding protein (CREB), MeCP2, and HDAC4 (*44*, *46*, *47*). We thus searched for putative consensus sequences for CaMKIV phosphorylation within the primary sequences of mouse and human REST [fig. S6 and (*48*)]. We identified three putative CaMKIV phosphorylation sites within the eight ZNF central cluster region of mouse and human REST, namely, Ser^177^, Thr^294^, and Ser^322^, located at position 1 of the ZNF linkers L1, L4, and L5. All three sites display the required −3 arginine residue but have the required hydrophobic residue at −4 rather than at the canonical −5 position (where they have a basic histidine). While all three putative sites have a hydrophobic residue at +4 that is highly preferred by CaMKI, Thr^294^ and Ser^322^ are particularly interesting because they display an acidic residue at +2 (glutamate), which is found in optimal consensus sequences for CaMKII. In contrast, Ser^177^ has a basic residue instead. The three putative phosphorylation sites were analyzed for their potential to be phosphorylated by CaMKs using the prediction software Musite and NetPhos3.1. As shown in table S1, in both human (hREST) and mouse (mREST) sequences, the Ser^322^ site has the highest phosphorylation potential, while Ser^177^ displays the lowest. In addition to these structural issues, the REST peptide linkers encompassing the Thr^294^ and Ser^322^ putative sites share a strong analogy with other transcription factors containing ZNF clusters, such as the transcription factor *Ikaros* (*49*), in which ZNF domains are usually organized in arrays joined by short 5- to 7-mer linker sequences that often start with a phosphorylatable residue. In the REST protein, the linker peptide containing the putative site 1 (Ser^177^) is the least conserved from mouse to man and bears no resemblance to the short linker peptides that join adjacent ZNF domains, being a long 36-mer peptide connecting ZNF1 to ZNF2. On the contrary, the 7-mer Thr^294^- and Ser^322^-containing linkers L4 and L5 closely resemble Ikaros ZNF linkers. For all these reasons, we focused on Thr^294^ and Ser^322^ and tested whether they could be phosphorylated by CaMKIV (Fig. 4A).

**Fig. 4.**
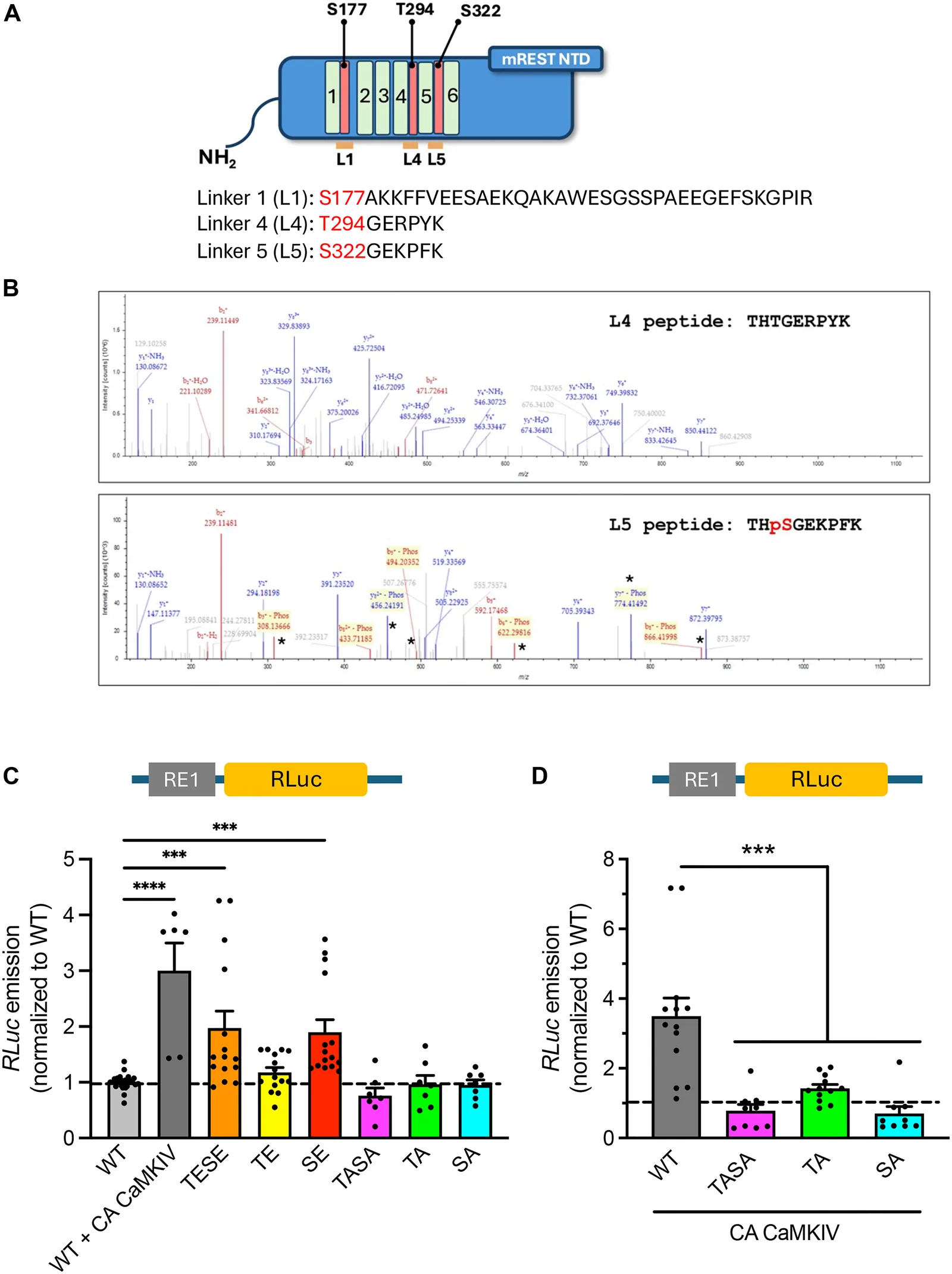
Ser^322^ in the linker between the fifth-to-sixth ZNF domains is the major CaMKIV phosphorylation site in mouse REST. (**A**) The Ser^177^, Thr^294^, and Ser^322^ phosphorylation sites encompass the linker peptides between the first and second (L1), the fourth and fifth (L4), and the fifth and sixth (L5) in mouse REST ZNF domains, respectively. (**B**) Tandem mass spectra for L4 (top) and L5 (bottom) tryptic peptides derived from *Myc*-tagged recombinant mouse REST expressed in HEK293T cells, affinity purified with anti-*Myc* magnetic beads, and phosphorylated on beads by purified CA CaMKIV. L5 shows phosphorylation of Ser^322^, as demonstrated by the corresponding diagnostic sequence fragment ions (asterisk in the spectrum). (**C** and **D**) A RE1-Luciferase construct, with the *RLuc* gene cloned downstream the promoter region containing triple RE1 sequence from *Scn2a*, was used to assess, in HEK293T cells, the extent of transcriptional repression exerted by the expression of WT REST or its phosphorylation site mutants Ser^322^>Glu/Thr^294^>Glu (TESE), Thr^294^>Glu (TE), Ser^322^>Glu (SE), Ser^322^>Ala/Thr^294^>Ala (TASA), Thr^294^>Ala (TA), and Ser^322^>Ala (SA) in the absence (C) or presence (D) of CA CaMKIV. The *RLuc* chemiluminescence ratio was normalized to the control WT REST and is shown as mean ± SEM. *RLuc* emission is increased by phosphomimic mutants SE and TESE, as well as by WT REST in the presence of CA CaMKIV, in comparison to the WT REST control, while the nonphosphorylatable mutants do not change it significantly. (C) ****P < 0.0001, WT versus WT + CA CaMKIV; ***P = 0.0003, WT versus TESE; ***P = 0.0009, WT versus SE. (D) ***P < 0.0001, WT versus TASA or SA; *P* = 0.0005, WT versus TA. One-way ANOVA/Dunnett’s tests (*n* = 6 to 18 independent experiments).

To this aim, we expressed *Myc*-tagged recombinant mouse REST in HEK293T cells, affinity purified it using anti-*Myc* magnetic beads, and phosphorylated it on the beads with purified CA CaMKIV. Purified phosphorylated mREST was subjected to tryptic cleavage and tandem mass spectrometry (MS/MS). Tandem mass spectra for L4 (top) and L5 (bottom) tryptic peptides revealed phosphorylation of Ser^322^ but not of Thr^294^, as demonstrated by the corresponding diagnostic sequence fragment ions (Fig. 4B).

The location of Thr^294^ and Ser^322^ in the very-short L4/L5 ZNF linkers of mouse REST resembles that of *Ikaros*, in which phosphorylation of the first residue in the short ZNF linkers impairs the binding of the transcription factor to DNA (*49*). This raises the possibility that phosphorylation of Ser^322^ (and possibly of Thr^294^ ) affects the interaction between REST and the RE1 motifs in the DNA, promoting cytoplasmic migration of REST, which eventually undergoes degradation. To verify this, we replaced either or both phosphorylatable residues with negatively charged glutamate or with alanine, generating single [Thr^294^>Glu (TE) and Ser^322^>Glu (SE)] or double (TESE) phosphomimic mutants, as well as single [Thr^294^>Ala (TA) and Ser^322^>Ala (SA)] or double (TASA) nonphosphorylatable mutants. The analysis of REST repressor activity was performed after transfecting HEK293T cells with REST phosphomimic and nonphosphorylatable mutants in the absence (Fig. 4C) or presence (Fig. 4D) of cotransfected CA CaMKIV, respectively. To uncover the mutants’ transcriptional effects, we used the gene reporter assay (see Fig. 1B). *RLuc* expression was significantly increased by expressing WT REST in the presence of CA CaMKIV, suggesting that phosphorylation impairs its transcriptional repressor activity. This hypothesis was confirmed by the significantly increased *RLuc* expression with both the SE and TESE mutants, but not TE or the nonphosphorylatable mutants, in the absence of CA CaMKIV, strongly implicating Ser^322^ as the major CaMKIV phosphorylation site (Fig. 4C). When CA CaMKIV was coexpressed with either WT REST or its nonphosphorylatable mutants, *RLuc* expression was markedly increased with WT REST, totally unaffected with the TASA and SA mutants, and only slightly increased with the TA mutant (Fig. 4D).

### The impaired repressor activity and degradation of REST phosphomimic mutants are due to their translocation from the nucleus to the cytoplasm

To further investigate the subcellular trafficking of WT REST and of its phosphomimic or nonphosphorylatable mutants, we transfected HEK293T cells with either WT REST or with the single or double REST phosphomimic and nonphosphorylatable mutants, using cytosolic mCherry as a transfection reporter and Hoechst staining to morphologically identify the nuclear areas (Fig. 5A). To uncover changes in the subcellular distribution of REST, we calculated the nuclear/cytosolic ratio of REST immunoreactivity (see Materials and Methods) by calculating the nuclear REST fraction, as the REST immunoreactivity overlapped with Hoechst staining, and the cytosolic REST fraction, as the difference between the total and the nuclear REST immunoreactivities. Expression of REST nonphosphorylatable mutants did not significantly alter the nuclear-to-cytoplasmic ratio of WT REST, which predominantly localizes to the nucleus to exert its repressor activity on target genes. In contrast, the SE but not the TE phosphomimic REST mutant displayed a markedly reduced nuclear-to-cytoplasmic ratio, suggesting that, after dissociating from DNA, the SE mutant is exported from the nucleus to the cytoplasm (Fig. 5B). An alternative analysis of the colocalization of REST immunoreactivity with Hoechst nuclear staining was performed by calculating the Pearson’s correlation coefficient (see Materials and Methods). While the Pearson’s coefficients of nonphosphorylatable REST variants, ranging between 0.17 and 0.26, indicate a predominant nuclear compartmentalization, the same analysis, applied to the expression of the phosphomimic REST mutants, confirmed the significant decrease in the extent of nuclear colocalization of the SE mutant (fig. S7).

**Fig. 5.**
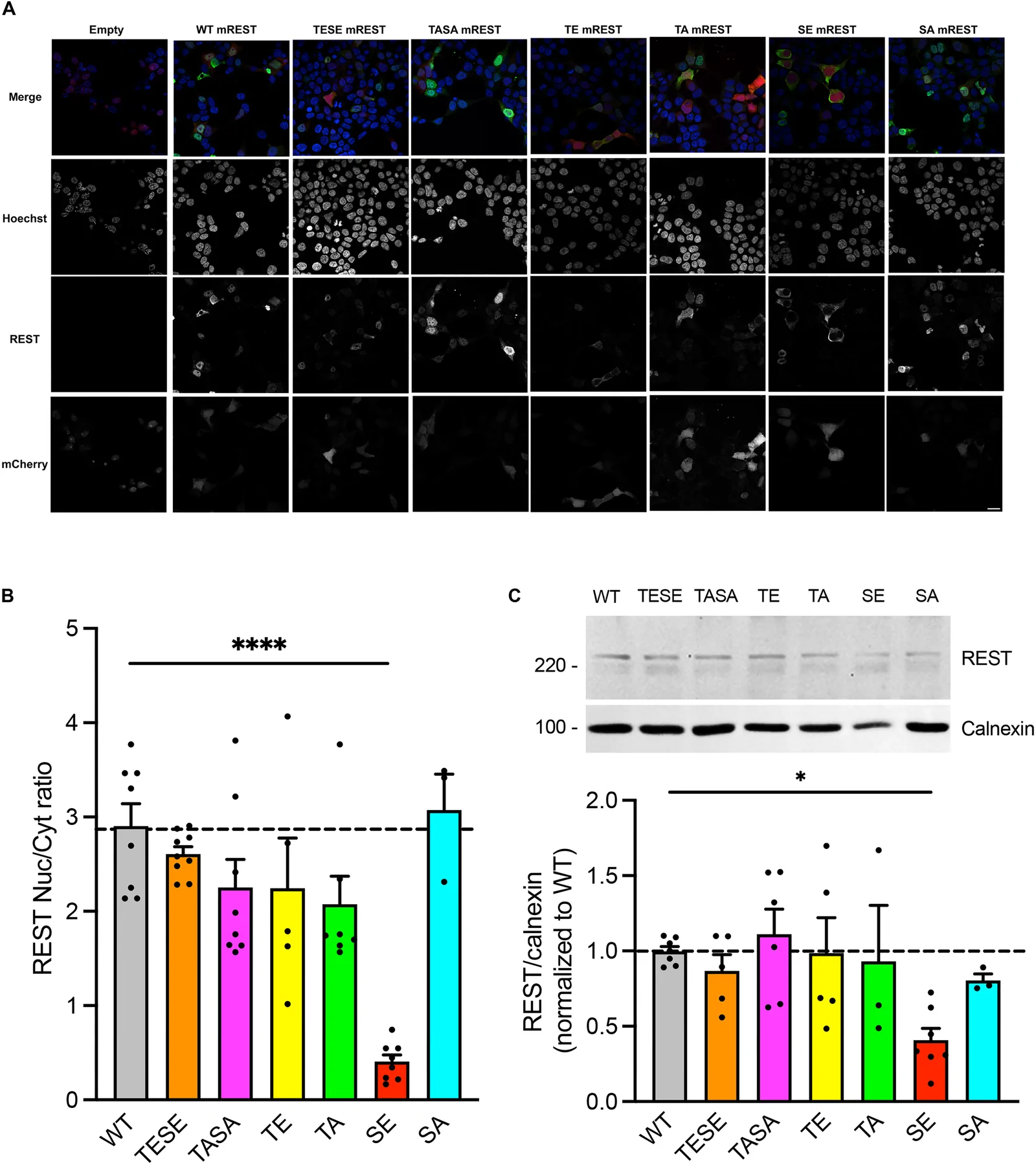
The phosphomimic SE REST mutant has an impaired repressor activity and shuttles to the cytosol, where it undergoes degradation in HEK293T cells. (**A** and **B**) HEK293T cells were transfected with WT REST or with its phosphorylation site mutants TESE, TE, SE, TASA, TA, and SA phosphomimic mutants with soluble mCherry as a reporter. (A) Representative samples of the seven experimental groups are shown, in which REST immunostaining, Hoechst nuclear staining, and mCherry fluorescence were used to evaluate the cytosolic/nuclear localization of REST mutants. Transduction of HEK293T cells with an empty vector was used as a negative control for REST immunoreactivity. (B) Nuclear/cytosolic ratio of REST immunoreactivity. The nuclear REST signal was defined as the fluorescence overlapping the Hoechst staining, while the cytosolic REST signal was obtained by subtracting the nuclear REST signal from the total REST immunofluorescence. Mean (±SEM) nuclear/cytosolic ratios of REST immunoreactivity calculated for WT REST and its phosphorylation mutants. ****P < 0.0001, SE versus WT. One-way ANOVA/Dunnett’s tests. Nuc/Cyt, nuclear/cytosolic. (**C**) HEK293T cells were transfected with WT REST or its phosphorylation site mutants TESE, TE, SE, TASA, TA, and SA. The levels of the phosphomimic mutant SE were strongly decreased in comparison to the levels of the other mutants. *P = 0.0441, SE versus WT. One-way mixed-effect ANOVA/Dunnett’s tests (*n* = 5 to 8 independent experiments).

Last, analysis of total REST protein levels under the same experimental conditions revealed significantly increased degradation of the SE phosphomimic mutant to the same extent as the nuclear escape. On the contrary, the expression of the TESE or TE phosphomimic mutants and of the nonphosphorylatable REST mutants was not associated with significant REST degradation (Fig. 5C). Together, the data indicate that phosphorylation of Ser^322^ mimics the effects of CA CaMKIV and may trigger a sequence of events comprising impairment of REST repressor activity, translocation from the nucleus to the cytoplasm, and proteolytic degradation.

### Phosphomimic substitutions destabilize the mREST:RE1 interaction interface and promote local structural collapse

To evaluate the impact of phosphomimic substitutions on the stability of the mREST:RE1 interaction interface, we conducted atomistic molecular dynamics (MD) simulations on both WT and mutant complexes. We focused on the mREST segment V130-H267. The mREST:RE1 complex was reconstructed using a multistep protocol comprising structural modeling, protein-DNA docking, complex refinement, and Zn^2+^ coordination optimization (figs. S8 to S13). The resulting structure was used as the starting conformation for three independent 1-μs replicate MD simulations (R1 to R3).

To monitor global structural deviations over time, we computed the root mean square deviation (RMSD) using all heavy atoms of the protein and the DNA. The RMSD profiles are stable around 2.8 to 4 Å, indicating no significant structural deviations from the starting configuration (fig. S14). We quantified the integrity of the protein-DNA interface by measuring the distance between a selected phosphate atom of the DNA backbone adjacent to the first ZNF module and the center of mass (c.m.) of the Zn-coordinating side-chain atoms (cysteine and histidine residues) as a distance-based descriptor. This distance was stable throughout all simulations of the WT system, with average values of 10.3 ± 2.4 Å (R1), 11.1 ± 1.5 Å (R2), and 7.6 ± 0.7 Å (R3), and no local structural deviations were observed (Fig. 6A), indicating that the WT mREST:RE1 complex maintains a stable interface over time.

**Fig. 6.**
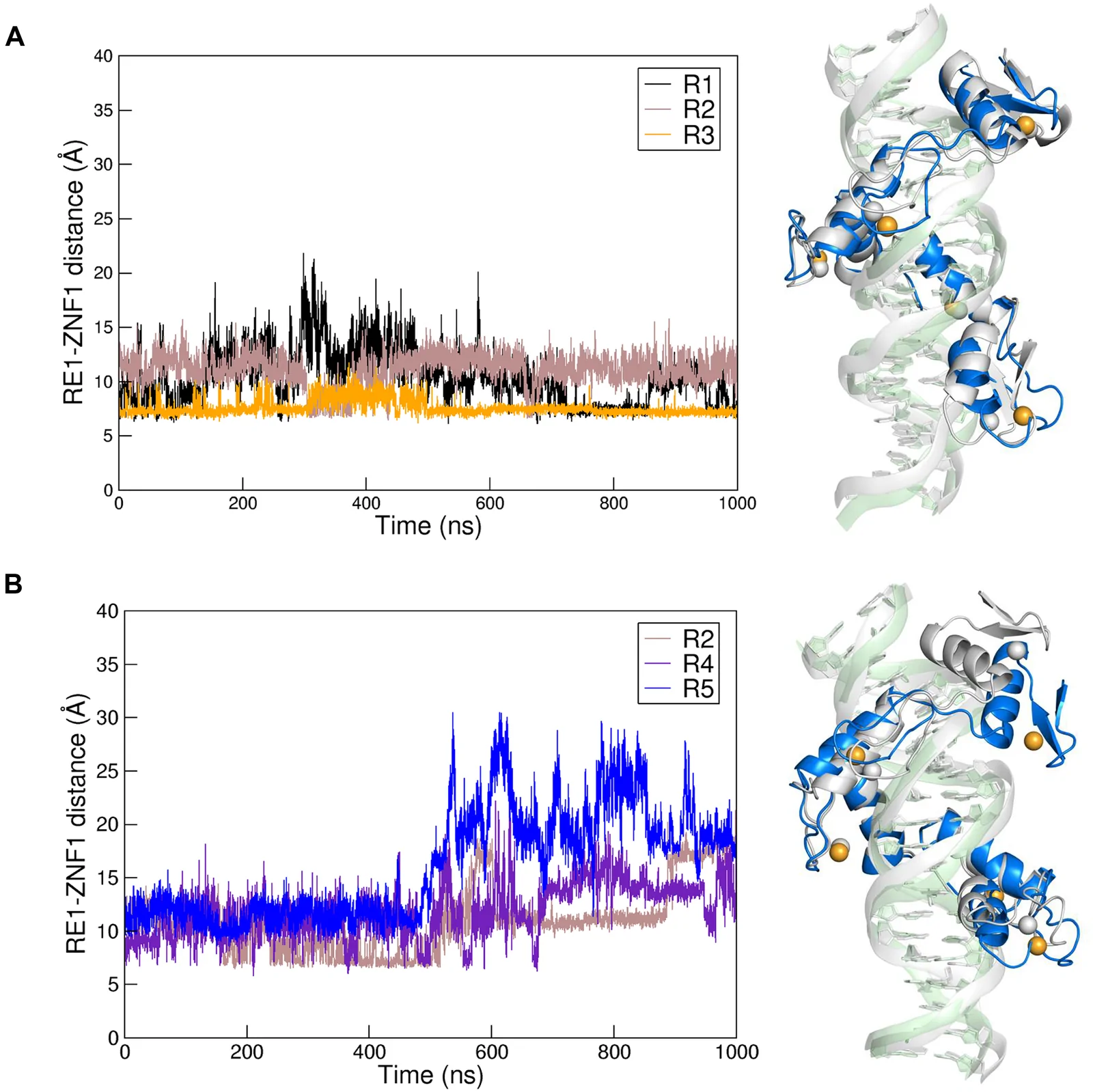
Stability of the mREST:RE1 interface in WT and SE phosphomimic complexes. (**A**) Left: Time evolution of the RE1-ZNF1 distance for three WT replicate simulations. Right: Structural superimposition of mREST:RE1 WT complex configurations at the beginning (gray) and at the end (green: DNA, blue: protein, orange: Zn ion) of one simulated trajectory. (**B**) Left: Time evolution of the RE1-ZNF1 distance in three SE replicas. Right: Structural superimposition of REST:RE1 SE mutant configurations at the beginning (gray) and at the time point corresponding to the maximum RE1-ZNF1 separation observed in the three trajectories.

To evaluate the potential destabilizing effects of phosphorylation-mimicking mutations, we generated two mREST variants: SE and TE, in which Ser^322^ and Thr^294^, respectively, were substituted with glutamate. Each mutant was simulated in nine independent 1-μs MD replicas. For the SE mutant, three replicas (R2, R4, and R5) showed an increase in the c.m.-phosphate distance to 11.2 ± 3.3 Å (R2), 11.8 ± 2.5 Å (R4), and 16.1 Å (R5), with maximum values reaching 24.8, 24.3, and 30.6 Å, respectively, indicating dissociation of the first ZNF domain from its DNA binding site (Fig. 6B). Three other replicas (R7, R8, and R9) exhibited structural irregularities, prompted by DNA termini fragilization, leading to complete system collapse in R9 (fig. S15). The remaining three trajectories (R1, R3, and R6) maintained stable interactions, with average distances of 8.8 ± 1.7, 7.5 ± 0.8, and 9.9 Å ± 1.8 Å and lower peak excursions (13.6 to 17.7 Å) (fig. S16). For the TE mutant, two replicas (R1 and R4) displayed interface dissociation, with average distances of 11.9 ± 2.2 and 10.3 ± 2.6 Å and maximum distance values of 22.4 and 25.0 Å (fig. S17A). Two trajectories (R8 and R9) showed DNA fragilization, and five replicas (R2, R3, R5, R6, and R7) remained structurally stable, with average distances ranging from 7.8 ± 1.0 to 11.6 ± 1.6 Å and peak values between 13.0 and 20.9 Å (fig. S17B). Together, these results show that both phosphomimic substitutions at Ser^322^ and Thr^294^ introduce measurable destabilization of the mREST:RE1 interaction interface, but that interface dissociation and structural fragilization are more likely and of greater impact in SE.

### The REST phosphomimic mutant impairs the homeostatic effects of REST overexpression on miniature EPSC frequency

To dissect the effects of REST phosphorylation on basal excitatory synaptic transmission, we transduced primary cortical neurons with lentiviruses encoding WT REST or its Ser^322^ phosphorylation mutants SA (nonphosphorylatable), or SE (phosphomimic), and recorded miniature EPSCs (mEPSCs) by patch clamp at days in vitro 14 (DIV14) (Fig. 7, A and B).

**Fig. 7.**
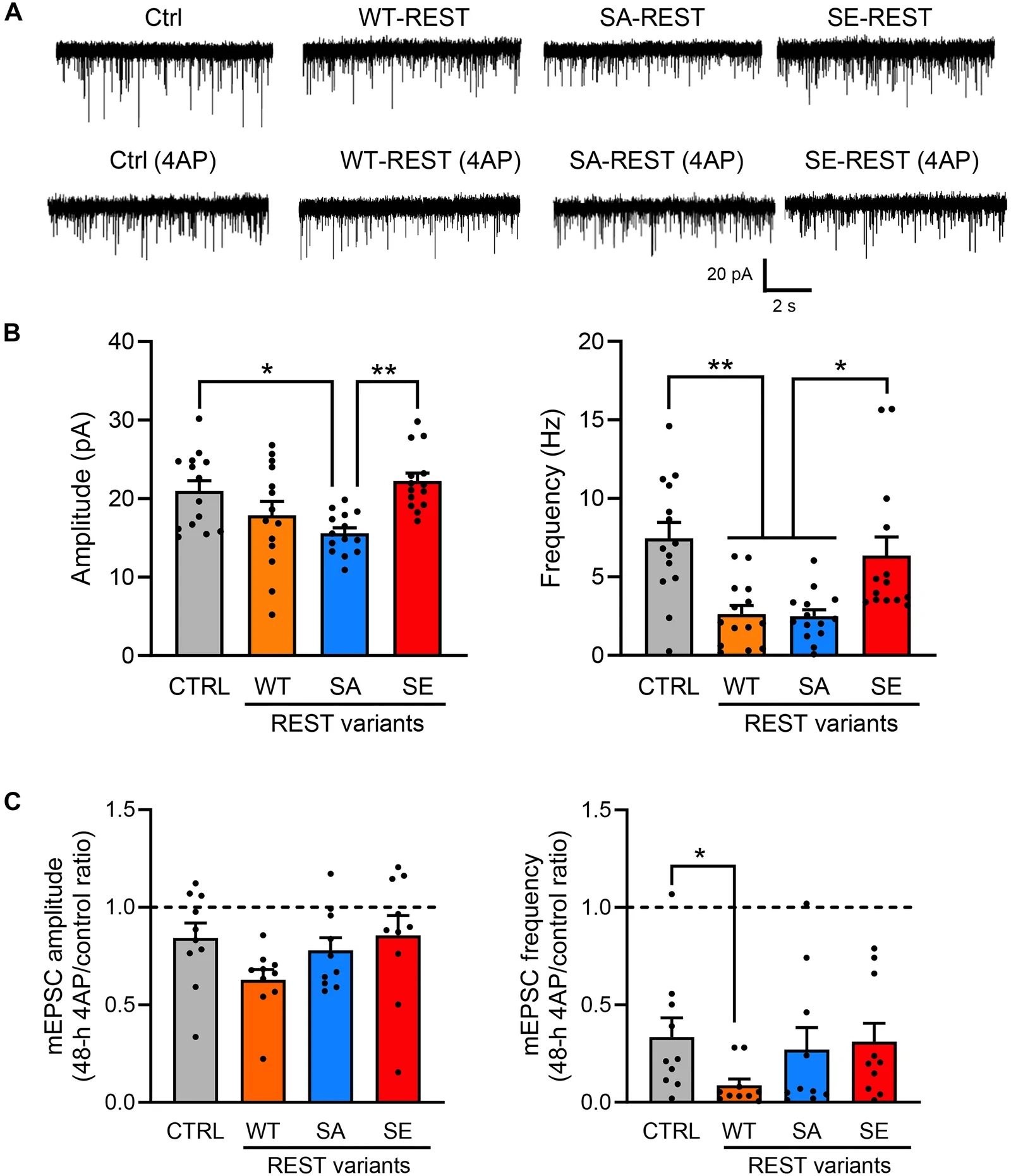
Pseudophosphorylation of REST at Ser^322^ abolishes the homeostatic effects of dephosphorylated REST on mEPSC frequency. (**A**) Representative traces of mEPSCs recorded in primary cortical neurons that were transduced at DIV7 with a control vector, WT REST, its nonphosphorylatable (SA) mutant, or its phosphomimic (SE) mutant and treated at DIV12 with either vehicle (top row) or 100 μM 4-aminopyridine (4AP; bottom row) for 48 hours. (**B**) Mean (±SEM) mEPSC amplitude (left) and frequency (right) in the four experimental groups treated with vehicle (*n* = 14 for each experimental group). One-way ANOVA/Tukey’s tests (amplitude: *P = 0.0197, Ctrl versus SA; **P = 0.0026, SE versus SA) (frequency: **P = 0.0011, Ctrl versus WT; **P = 0.0008, Ctrl versus SA; *P = 0.0164, WT versus SE; *P = 0.0120, SA versus SE). (**C**) Mean (±SEM) mEPSC amplitude (left) and frequency (right) in the four experimental groups treated with 100 μM 4AP for 48 hours starting from DIV12 and analyzed at DIV14. mEPSC amplitude and frequency were normalized over the control/vehicle group. The broken horizontal line represents the absence of homeostatic effects in response to 4AP-induced hyperactivity (*n* = 10 for each experimental group). Kruskal-Wallis one-way ANOVA/Dunn’s tests (amplitude: not significant; frequency: *P = 0.0452, Ctrl versus WT). h, hour.

Under control conditions, the expression of either WT REST or its dephosphomutant induced a decrease in the mEPSC frequency, as previously reported for endogenous REST (*12*). Notably, the phosphomimic mutant was virtually ineffective at altering mEPSC frequency, confirming its inability to bind the RE1 domain reported above (Fig. 7B). The effects of either WT REST or SA REST on mEPSC amplitude were less pronounced, consistent with the predominant effects of REST on presynaptic function, and reached significance only with the nonphosphorylatable mutant. This effect was also abolished by the REST SE phosphomimic variant, which returned the amplitude to the same level observed in the absence of REST expression (Fig. 7B). We also overexpressed WT REST, the nonphosphorylatable SA mutant, or the phosphomimic SE mutant in REST-silenced neurons. The results were very similar, showing that while both WT and SA-REST were effective in rescuing the silencing phenotype in mEPSC frequency, the phosphomimic SE variant was completely ineffective (fig. S18).

REST is known to operate in mature neurons by tuning the expression of the synaptic machinery to maintain synaptic stability in response to hyperactivity (*9*, *10*). Thus, we investigated the effects of the nonphosphorylatable SA mutant and the phosphomimic SE mutant on the downscaling of excitatory synaptic transmission in primary cortical neurons subjected to chronic hyperexcitability induced by 48 hours of exposure to the convulsant 4-aminopyridine (4AP; Fig. 7, A and C). When neurons were treated with 4AP and recorded 48 hours later, the expression of WT REST increased the extent of the homeostatic decrease in mEPSC frequency, as previously described (*12*), while the two phosphorylation mutants were not significantly different from the control, suggesting that the boosting of the homeostatic REST response needs phosphorylation/dephosphorylation cycling. No significant alterations were observed in the homeostatic response in mEPSC amplitude after expression of either WT REST or its phosphomutants with respect to control (Fig. 7C).

### The CaMKIV/REST interaction underlies the homeostatic effects of CaMKIV on the amplitude of excitatory synaptic transmission in primary neurons

CaMKIV is known to couple increases in intracellular Ca^2+^ to compensatory changes in excitatory synaptic transmission (*50*). Having identified a mechanistic link between CaMKIV activation and REST activity and half-life, we investigated the interplay between CaMKIV and REST on excitatory synaptic transmission by knocking down REST (REST-KD) and either down-regulating CaMKIV expression (CaMKIV-KD) or expressing CA CaMKIV in primary cortical neurons.

Primary neurons in which CaMKIV was knocked down displayed a significant increase in mEPSC amplitude and no effects on the mEPSC frequency, in agreement with previous reports (*50*) and indicating a tonic homeostatic control of mEPSC amplitude, but not frequency, by CaMKIV. On the contrary, REST-KD increased mEPSC frequency without affecting mEPSC amplitude, confirming the predominant presynaptic effects of REST modulation. After combining both treatments, REST-KD antagonized the effects of CaMKIV-KD on mEPSC amplitude, with a significant interaction between the two treatments, whereas CaMKIV-KD did not significantly affect the response of mEPSC frequency to REST-KD (Fig. 8, A and B).

**Fig. 8.**
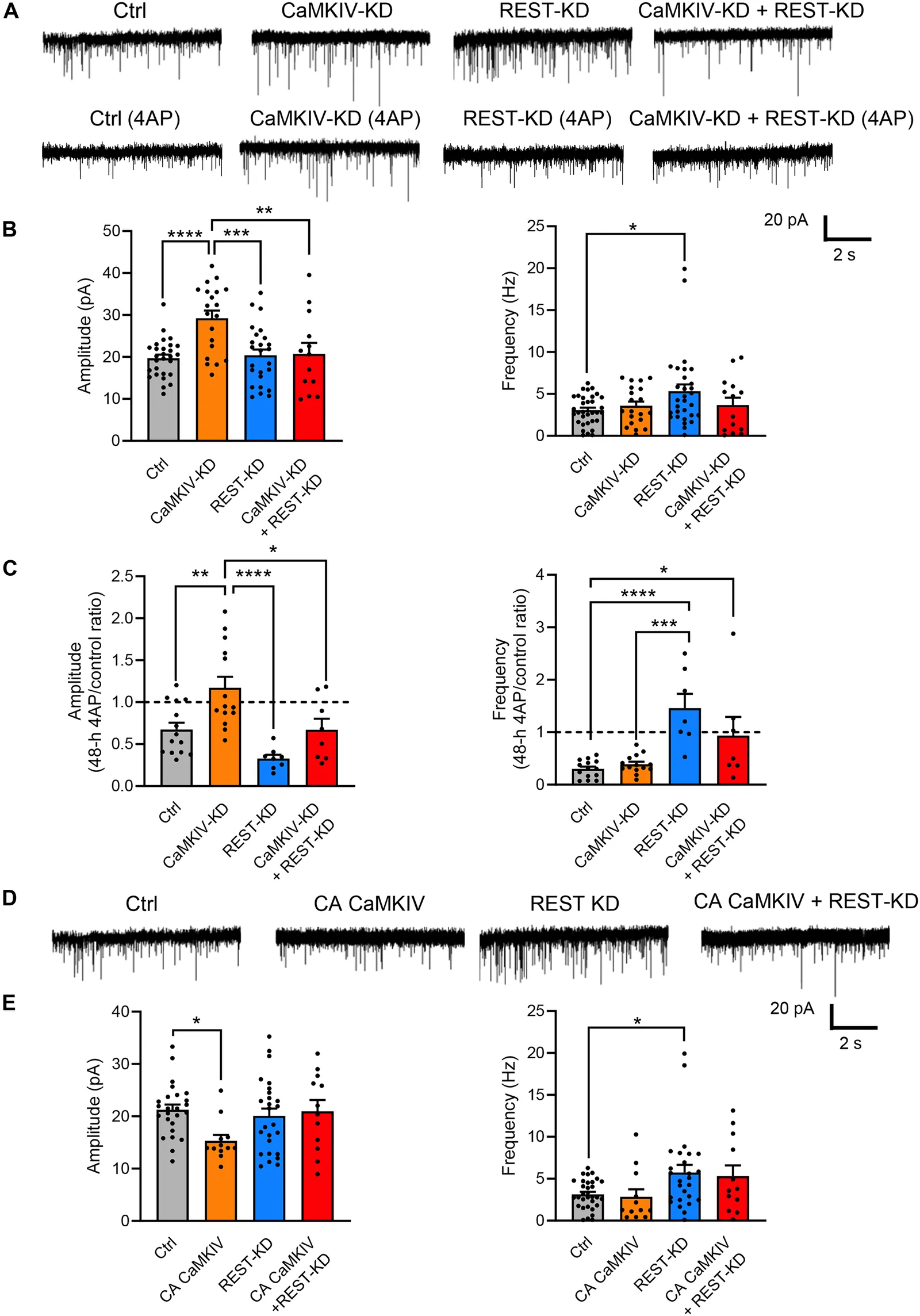
REST knockdown antagonizes the effects of CaMKIV knockdown on excitatory synaptic transmission in primary neurons. (**A** to **C**) Interaction between knockdown of REST and CaMKIV. (A) Representative traces of mEPSCs recorded in primary cortical neurons that were transduced at DIV7 with scrambled (Ctrl), shREST (REST-KD), and shCaMKIV (CaMKIV-KD) or cotransduced with both shREST and shCaMKIV (CaMKIV-KD + REST-KD) and treated at DIV12 with either vehicle (top row) or 100 μM 4AP (bottom row) for 48 hours. (B) Mean (±SEM) mEPSC amplitude (left) and frequency (right) (*n* = 14 to 33) recorded in the experimental groups treated with vehicle. Two-way ANOVA (amplitude: *F*_1,81_ = 8.460, *P* interaction = 0.0047; frequency: *F*_1,93_ = 2.588, *P* interaction = 0.1110) followed by Tukey’s or Holm-Šídák’s test (amplitude: ****P < 0.0001, Ctrl versus CaMKIV-KD; ***P = 0.0006, CaMKIV-KD versus REST-KD; **P = 0.0060, CaMKIV-KD versus CaMKIV-KD + REST-KD) (frequency: *P = 0.0317, Ctrl versus REST-KD). (C) Mean (±SEM) mEPSC amplitude (left) and frequency (right) in the experimental groups treated with 4AP. mEPSC amplitude and frequency were normalized over the control/vehicle group. The broken horizontal line represents the absence of homeostatic effects in response to 4AP-induced hyperactivity (*n* = 7 to 14). Two-way ANOVA (amplitude: *F*_1,40_ = 0.4507, *P* interaction = 0.5058; frequency: *F*_1,38_ = 3.590, *P* interaction = 0.0658) followed by Tukey’s test (amplitude: **P = 0.0050, Ctrl versus CaMKIV-KD; ****P < 0.0001, CaMKIV-KD versus REST-KD; *P = 0.0193, CaMKIV-KD versus CaMKIV-KD + REST-KD) (frequency: ****P < 0.0001, Ctrl versus REST-KD; *P = 0.0403, Ctrl versus CaMKIV-KD + REST-KD; ***P = 0.0002, CaMKIV-KD versus REST-KD). (**D** and **E**) Interaction between REST knockdown and CA CaMKIV. (D) Representative traces of mEPSCs recorded in primary cortical neurons transduced with scrambled (Ctrl), CA CaMKIV, and shREST (REST-KD) or cotransduced with CA CaMKIV and shREST (CA CaMKIV+REST-KD) at DIV7 and recorded at DIV14. (E) Mean (±SEM) mEPSC amplitude (left) and frequency (right) (*n* = 12 to 33). Two-way ANOVA (amplitude: *F*_1,71_ = 5.117, *P* interaction = 0.0268; frequency: F_1,78_ = 0.0060, *P* interaction = 0.9382) followed by Tukey’s test (amplitude: *P = 0.0345, Ctrl versus CA CaMKIV; frequency: *P = 0.0322, Ctrl versus REST-KD).

To further confirm the results of the interaction between REST and CaMKIV, we knocked down the kinase by RNA interference in primary cortical neurons derived from conditional REST knockout (cKO) mice (Rest^GTi^) in which the *Rest* gene was fully deleted by transduction of Cre recombinase (Cre) or left intact by transduction with a catalytically inactive Cre deletion mutant (ΔCre). The results were entirely consistent with experiments using RNA interference to knock down REST. In addition, in these cKO neurons, CaMKIV-KD increased mEPSC amplitude without affecting EPSC frequency. REST KO neurons experienced an increase in excitatory strength with augmented mEPSC frequency and no significant effects on mEPSC amplitude, as shown by RNA interference, while concomitant REST deletion virtually abolished the effects of CaMKIV silencing (fig. S19).

Both CaMKIV and REST are known to couple increases in intracellular Ca^2+^ to compensatory changes in excitatory synaptic transmission (*9*, *10*, *50*). Thus, we sought to apply a homeostatic paradigm that scales down excitatory transmission in response to sustained hyperactivity, achieved by incubating primary neurons with 4AP for 48 hours and by alternately or concurrently applying CaMKIV and REST suppression via RNA interference (Fig. 8, A and C). CaMKIV-KD significantly counteracted the homeostatic effect of the kinase on mEPSC amplitude, while it did not affect the homeostatic downscaling of mEPSC frequency. On the other hand, REST-KD did not significantly affect the homeostatic response of mEPSC amplitude, while it fully abolished the homeostatic downscaling of mEPSC frequency. However, while the effects of REST-KD were not altered by concomitant CaMKIV-KD, REST-KD significantly counteracted the effect of CaMKIV-KD on mEPSC amplitude by reinstating a homeostatic response (Fig. 8C).

To further clarify the REST-CaMKIV interaction, we combined CA CaMKIV expression with REST-KD. CA CaMKIV decreased the amplitude but not the frequency of mEPSCs, supporting the results obtained with CaMKIV-KD. REST-KD had no effect per se on the amplitude but, when combined with CA CaMKIV, antagonized the effect of CaMKIV, confirming the existence of a significant interaction between REST and CaMKIV. Consistent with the above results, CA CaMKIV did not affect mEPSC frequency, whereas REST-KD increased mEPSC frequency independently of CaMKIV activity (Fig. 8, D and E).

Together, the data indicate that the Ca^2+^-dependent homeostatic effects of CaMKIV, predominantly exerted at the postsynaptic level, are dependent, at least in part, on the presence of REST, while the effects of REST-KD or cKO are not greatly affected by the modulation of CaMKIV activity, posing REST downstream of the kinase in the integrated control of excitatory strength.

## DISCUSSION

### CaMKIV and REST are major homeostatic regulators of transcription

Mature neurons can regulate their network activity to maintain a target level over time through homeostatic plasticity mechanisms regulating intrinsic excitability and synaptic transmission. Several distinct homeostatic transcriptional and posttranslational mechanisms have been identified (*10*), triggered by a proxy for hyperactivity, such as the cytosolic Ca^2+^ concentration, which serves as an integrated activity signal, as most studies indicate. Calcium increases activate CaM-dependent enzymes, including the array of CaMKs that, in turn, act at the posttranslational and transcriptional levels, modifying function and expression of ion channels and synaptic proteins (*41*). A key role in the activity-regulated transcriptional mechanisms is played by transcription factors that can be directly stimulated by hyperactivity [e.g., cFos and Arc; (*51*)] or activated by phosphorylation. Among CaMKs, CaMKIV, once activated by CaMKK, enters the nucleus and phosphorylates a series of transcription factors such as CREB and MeCP2. A prominent transcription factor that responds rapidly and directly to hyperactivity is REST, an epigenetic master regulator that represses transcription of a large cluster of neuron-specific genes by interacting with RE1 motifs in their promoters (*2*, *9*). In addition to its fundamental role in REST down-regulation during neuronal differentiation, which enables full expression of the neuronal phenotype, REST also acts as an important homeostatic regulator in mature neurons, protecting them from hyperactivity and senescence. Dysregulation of REST in expression or mislocalization was found to associate with brain diseases, such as epilepsy, stroke, neuroinflammation, and neurodegeneration [(*17*, *19*, *21*–*27*); see (*28*) for review]. These considerations emphasize the importance of a tight regulation of REST-DNA interactions, nuclear-cytoplasmic shuttling, and cytoplasmic fate.

### REST is a phosphorylation substrate of CaMKIV in neurons

REST was reported to be phosphorylated by CK1 and ERK1/2 at distinct sites in its C-terminal region within phosphodegron sequences that direct the protein to the proteasome degradation machinery (*36*–*39*). These mechanisms are thought to be important for decreasing REST levels in neural progenitors, thereby permitting neuronal differentiation. As calcium is believed to be the most likely activator of the homeostatic responses mediated by REST and REST contains several consensus sequences for CaMKs, we analyzed the possibility that REST is phosphorylated by CaMKs, with phosphorylation modulating its transcriptional activity and/or cellular fate. We found that (i) REST is phosphorylated by CaMKIV at a major phosphorylation site between the fifth and sixth ZNF domains (Ser^322^), (ii) phosphorylation by CaMKIV inhibits the transcriptional repressor activity of REST by decreasing its affinity for RE1 motifs, (iii) dissociation from DNA favors shuttling of phospho-REST from the nucleus to the cytoplasm where it undergoes protease-mediated degradation, and (iv) the homeostatic effects of CaMKIV on excitatory synaptic strength require the presence of REST.

### Identification of CaMKIV phosphorylation sites

Structural studies have shown that the consensus sequences for phosphorylation by CaMKs are, in large part, shared by CaMKI, II, and IV and conform to the following minimal motif: Hyd-X-R-X-X-S*/T*-X-X-X-Hyd (*52*, *53*). While the −3 Arg and −5 hydrophobic residue seem important for all kinases, CaMKI prefers a +4 hydrophobic residue, while CaMKII prefers a +2 acidic residue. The motif for CaMKIV is more degenerate with fewer requirements than the other kinases, supporting the designation of CaMKIV as a multifunctional protein kinase. Of the three putative CaMKIV phosphorylation sites (Ser^177^, Thr^294^, and Ser^322^) within the eight ZNF central cluster region of mouse and human REST, analysis of the location of the basic, hydrophobic, and acidic residues in the consensus sequences found that the sequences encompassing Thr^294^ and Ser^322^ conform better to these requirements, in addition to being the most evolutionarily conserved of the three putative sites. This observation was fully confirmed by the predictions of putative phosphorylation sites using the software Musite and NetPhos3.1 in both mouse and human REST. Moreover, the REST peptide linkers encompassing the Thr^294^ and Ser^322^ putative sites share a strong analogy with other DNA binding transcription factors that contain ZNF clusters organized in arrays, joined by short 5- to 7-mer linker sequences. In the four-ZNF–containing transcription factor *Ikaros*, for example, ZNF domains are joined by three 5-mer peptides starting with a phosphorylatable residue that, if phosphorylated, impairs the binding of the transcription factor to DNA (*49*). MS analysis of in vitro phosphorylated recombinant REST revealed detectable phosphate incorporation at Ser^322^, indicating that Ser^322^ is the major CaMKIV site in REST.

### REST phosphorylation destabilizes the coordination geometry with DNA

To explore the mechanistic basis by which phosphorylation modulates REST-DNA binding affinity, we integrated biochemical analysis with atomistic MD simulations of the mREST:RE1 complex in both WT and phosphomimic variants. While the biochemical experiments establish that CaMKIV-mediated phosphorylation reduces REST affinity for RE1 motifs, the MD simulations further corroborate this effect and provide a high-resolution structural rationale for how perturbations at the protein-DNA interface weaken binding. Specifically, the simulations revealed that the SE substitution, mimicking phosphorylation at Ser^322^, induces marked destabilization of the ZNF-DNA interface. In three of nine independent SE trajectories, we observed dissociation of the first ZNF domain from its DNA binding site, an event that was never observed in WT simulations. Moreover, in several SE trajectories, destabilization propagated beyond the local interface, leading to fragilization of the DNA termini and, in some cases, collapse of the full complex. Although some reduction in interface stability was also observed in some TE simulations, dissociation events were less frequent and were not accompanied by complex-wide structural collapse, indicating a more spatially restricted effect on DNA binding. These computational findings are consistent with prior experimental observations showing that phosphorylation within or near ZNF domains can impair DNA binding affinity. Phosphorylation of conserved linker sequences in Cys^2^-His^2^–type ZNF proteins, such as Ikaros and Sp1, has been shown to abolish DNA binding during mitosis by introducing conformational constraints or electrostatic repulsion (*49*, *54*, *55*). The functional consequence of the SE substitution on REST-DNA binding was fully confirmed by the *RLuc* transcriptional experiments, in which the phosphomimic mutants SE and TESE removed the REST transcriptional repression. In line with this mechanism, the MD simulations reveal the underlying atomic-level process: A substitution between ZNF5 and ZNF6 directly perturbs the local coordination geometry and weakens contacts with DNA. This provides quantitative MD-based evidence for a site-specific posttranslational modification within a ZNF module that destabilizes a DNA-protein complex at atomic resolution. The absence of dissociation or collapse in WT simulations underscores the specificity of the phosphomimic effect and supports a model in which phosphorylation triggers REST disengagement from DNA, followed by nuclear export and cytosolic degradation. Together, the biochemical and MD results define a distinct layer of REST regulation that acts directly through the DNA binding domain, providing a structural basis for linking signaling pathways to gene-regulatory outcomes and complementing the previously described regulation mediated by C-terminal phosphodegrons.

### Effects of REST phosphorylation on nuclear-cytoplasmic shuttling

Release of CaMKIV-phosphorylated REST from DNA promotes its export to the cytoplasm, where it undergoes degradation. There are similarities and differences compared with previous reports on REST phosphorylation. Both CK1 and ERK1/2 are fundamental signaling pathways to achieve REST down-regulation during neural development. Their phosphorylation creates phosphodegrons in the C-terminal region of REST, far from the DNA-interacting ZNF domains, which directly target REST for proteasomal degradation (*36*–*39*). Given the location of the CaMKIV phosphorylation site within the ZNF domain, REST degradation is likely the consequence of its dissociation from DNA and cytoplasmic migration rather than the result of the formation of a phosphodegron that recruits ubiquitin ligases. Our results show that CaMKIV-phosphorylated REST is not targeted to the proteasome. Thus, CaMKIV may act by terminating REST activity through a sequence of events initiated by CaMKIV phosphorylation in the nucleus, dissociation of phospho-REST from the DNA, its shuttling to the cytoplasm, and the subsequent degradation. Moreover, REST degradation is resistant to the inhibition of organelle acidification by bafilomycin A1, indicating that it does not depend on low-pH–active proteases present in lysosomes. Rather, its degradation is strongly inhibited by CA074, a specific Cathepsin B inhibitor, which has recently been shown to be active also at extralysosomal cytosolic localizations (*56*, *57*). Thus, the available evidence suggests that REST degradation is mediated by Cathepsin B, most likely acting at cytosolic pH. However, we cannot rule out that REST is also degraded by the lysosome-resident Cathepsin B, regardless of the acidic milieu of these organelles (*58*). Thus, CaMKIV may act by terminating REST activity through a sequence of events initiated by CaMKIV phosphorylation in the nucleus, dissociation of phospho-REST from the DNA, its shuttling to the cytoplasm, and the subsequent degradation.

### REST phosphorylation affects synaptic transmission

As phosphorylation of REST at Ser^322^ impairs its repressor activity at target genes, including pre- and postsynaptic actors, it was important to assess its consequences on synaptic transmission. We confirmed that the main effect of dephosphorylated REST occurs at the presynaptic level, decreasing synaptic event frequency, an effect abolished by phosphorylation. The fact that both nonphosphorylatable and phosphomimic mutants inhibited the homeostatic downscaling of the frequency of synaptic events after chronic hyperactivity suggests, however, that a physiological phosphorylation/dephosphorylation cycling is necessary to preserve the effect of REST in the homeostatic response to hyperactivity. Dephosphorylated REST also has a smaller inhibitory effect on mEPSC amplitude, which is reversed by phosphorylation, and this may be ascribed to the repression of postsynaptic genes, such as the RE1-containing *Gria2* gene encoding the GluA2 subunit of the AMPA receptors (*59*–*61*).

### The CaMKIV-REST interplay in synaptic homeostasis

The interaction between two well-characterized homeostatic factors, CaMKIV and REST, which are coactivated in response to intracellular Ca^2+^ fluxes during hyperactivity, is of physiological interest. REST responds rapidly to hyperactivity by increasing expression, migrating into the nucleus, and repressing target genes (*11*, *13*). CaMKIV is highly expressed in neurons, where it acts downstream of Ca^2+^ signaling. Activated by CaMKK phosphorylation, it migrates to the nucleus and modulates the transcription of various genes by phosphorylating transcription factors (*44*). In addition to being a CaMKIV substrate and modulating homeostatic plasticity in the frequency domain (presynaptic effect), REST also appears to act as a downstream CaMKIV effector necessary for the homeostatic modulation of mEPSC amplitude (postsynaptic effect) exerted by the kinase. As knockdown or genetic deletion of REST abolishes the bidirectional modulation of mEPSC amplitude by CaMKIV and CaMKIV phosphorylation of REST disrupts its interaction with DNA, the effect can be attributed to the release of genes that modulate mEPSC amplitude from REST-mediated transcriptional repression or enhancement. It has been reported that REST can occasionally induce transcriptional activation by favoring the expression of other genes involved in plasticity and synaptic transmission, such as *Npas4* [(*43*); see Fig. 2D], *L1CAM* (*62*), *TET3* (*63*), or *EAAT2* (*64*). As Ca^2+^ is believed to be the most likely activator of the homeostatic responses mediated by both REST and CaMKIV, the results suggest that the homeostatic neuronal responses to hyperactivity may require an interplay between CaMKIV and REST, which may define the synaptic site and temporal window of activation of the homeostatic response. Termination of REST activity may involve a sequence of events initiated by CaMKIV-mediated phosphorylation in the nucleus, dissociation of phospho-REST from the DNA, shuttling to the cytoplasm, and subsequent degradation (fig. S20). It is tempting to speculate that the elucidated CaMKIV-REST interplay has an important regulatory role in the homeostatic scaling of synaptic strength at both pre- and postsynaptic levels, as well as in the return of the increased REST levels to baseline following the homeostatic response to hyperactivity.

In conclusion, we identified a signaling network that controls the homeostatic transcriptional response to hyperactivity, involving two well-known transcriptional regulators, REST and CaMKIV, acting predominantly at the presynaptic and postsynaptic levels, respectively. A deeper understanding of CaMKIV-mediated REST phosphorylation dynamics could uncover previously unknown regulatory nodes with therapeutic potential in neurological disorders.

## MATERIALS AND METHODS

### Materials and experimental animals

#### Experimental animals

WT C57BL/6J male mice were purchased from Charles River (Calco, Italy). Mice were maintained on a 12:12-hour light/dark cycle at constant temperature (22° ± 1°C) and humidity (60 ± 10%), with water and pellet diet ad libitum*.* GTinvRest (RestGTi) mice (*65*) were obtained by G. Mandel (Portland, OR) and the German Gene Trap Consortium (GGTC Partners) and kept on a C57BL/6J background. All experiments were conducted in accordance with the guidelines of the European Community Council (Directive 2014/63/EU of 15 May 2014) and were approved by the Italian Ministry of Health (Authorization #427/2021-PR).

#### Materials

The HEK cell line 293 expressing the large T antigen of simian virus 40 (HEK293T #HTL04001) was purchased from the Interlab Cell Line Collection located at IRCCS Ospedale Policlinico San Martino (Genova, Italy). W7, KN92, KN93, CA074, E64, bicuculline, CGP58845, d-aminophosphovalerate, and tetrodotoxin were from Tocris (Bristol, UK). CsA, CHX, MG-132, leupeptin, and bafilomycin A1 were from Merck Life Science (Milan, Italy). All other chemicals, unless otherwise specified, were from Sigma-Aldrich (Milano, Italy)

### Primary neuron cultures

Cultures of primary cortical neurons were obtained from either WT C57BL/6 or GTinvRest mouse embryos, as previously described (*66*–*68*). These cultures are highly homogeneous, containing ∼75% excitatory neurons with a recognizable pyramidal shape, ∼20 to 25% glutamic acid decarboxylase–positive inhibitory neurons, a very low percentage of astrocytes (5 to 7%), and no microglial cells due to the embryonic nature of the preparation. Briefly, cerebral cortices from embryonic day 17 (E17) to E18 embryos were dissociated by enzymatic digestion with 0.125% trypsin for 30 min at 37°C, followed by trituration with a fire-polished Pasteur pipette. Primary cultures of dissociated cortical neurons were subsequently plated onto poly-l-lysine (0.1 mg/ml; Merck Life Science)–coated 18-mm glass coverslips (6 × 10^4^ cells per coverslip for immunofluorescence and electrophysiology experiments) and poly-l-lysine (0.01 mg/ml)–coated 35-mm wells (5 × 10^5^ cells per well for molecular biological and biochemical experiments). Neurons were maintained in a culture medium consisting of Neurobasal, supplemented with B27 [1:50 (v/v)], GlutaMAX [1% (w/v)], and penicillin-streptomycin (1%) (Thermo Fisher Scientific, Monza, Italy), and kept at 37°C in a 5% CO_2_ humidified atmosphere up to DIV20.

### Manipulations of CaM-dependent pathways

Neurons were treated for 24 hours before harvesting with the following compounds: W7 (20 μM), CsA (1 μM), KN93 (10 μM), and KN92 (10 μM). All drugs were obtained from Tocris. Dimethyl sulfoxide (DMSO; Sigma-Aldrich, catalog no. D2650, Milan, Italy) was used as a vehicle control, as specified by the experimental conditions. To specifically alter their CaMK activities, HEK293T cells were alternatively transfected with the following plasmids using Lipofectamine 2000 (Thermo Fisher Scientific):

pCMV-HA CaMK1 (WT, CA, or KD; kind gift of T. Kaitsuka, Kumamoto University, Kumamoto, Japan);

pBS-EGFP-MT-CaMK2A (#AJ890283, EMBL Database), pBS-EGFP-MT-CaMK2A K42R (CA), and pBS-EGFP-MT-CaMK2A T286D (KD), kind gifts from A. Thalhammer (University of Trieste, Italy);

pRSV-CaMK4WT (#45062; Addgene, Watertown, MA), pRSV-CaMK4 CA (1 to 313) (#45063; Addgene) (*69*), and pRSV-CaMK4 KD, which was mutagenized using pRSV-CaMKIV WT as template.

### REST expression in HEK293T cells

HEK293T cells at ∼70% confluency in 15-cm plates were transfected with 9.2 μg of pCS-myc-mREST per plate (*11*). Cells were washed and collected 48 hours after transfection in phosphate-buffered saline (PBS). Cell lysates were prepared using the Pierce c-Myc-Tag Magnetic IP/Co-IP Kit (Thermo Fisher Scientific) according to the manufacturer’s instructions. Briefly, cells were collected by centrifugation at ×100*g* for 5 min. Pellets were resuspended in 6 ml of Co-IP buffer (Thermo Fisher Scientific), incubated on ice for 10 min, and then centrifuged at 18,500*g* for 15 min at 4°C. The supernatant was sonicated at 25% power for 5 s on ice. The protein concentration was determined using the bicinchoninic acid (BCA) assay with the Pierce BCA Protein Assay Kit (Thermo Fisher Scientific). The remaining total lysates were concentrated using Amicon Ultra Centrifugal Filter (50-kDa MWCO; Merck Life Science) at ×18,500*g* for 20 min at 4°C. Anti–c-Myc magnetic beads were washed three times in tris-buffered saline containing Tween 20 (TBS-T) [25 mM tris, 0.15 M NaCl, and 0.05% (v/v) Tween 20].

### On-bead REST phosphorylation by CaMKIV

Approximately 200 μg of the concentrated total lysate was incubated with Pierce anti–c-Myc magnetic beads (Thermo Fisher Scientific) that were prewashed according to the manufacturer’s instructions, diluted 1:15 with TBS-T buffer, and incubated at room temperature (RT) for 30 min with rotation. Beads, collected on the magnetic stand and washed three times in TBS-T, were resuspended in alkaline phosphatase buffer [5 mM tris-Cl (pH 8.7), 10 mM NaCl, 1 mM MgCl_2_, and 0.1 mM dithiothreitol (DTT)] supplemented with protease inhibitor cocktail (Merck Life Science) and treated with 25 U of alkaline phosphatase (Merck Life Science) for 30 min at 30°C. The reaction was stopped by removing the supernatant on the magnetic stand and washing the beads twice with 200 μl of TBS-T buffer supplemented with 10 mM EDTA and protease inhibitors. The beads were subsequently resuspended in in vitro phosphorylation assay buffer (50 mM Hepes at pH 7.4, 10 mM MgCl_2_, 1 mM EGTA, and 0.4 mM DTT), supplemented with protease and phosphatase inhibitors, 60 μM Mg^2+^–adenosine 5′-triphosphate (ATP), and 600 nM CaM (Merck Life Science). Either 1 μg of active recombinant CaMKIV (Merck Life Science) or vehicle (negative control) was added to the samples, and the mix was incubated for 1 hour at RT under rotation. The on-bead kinase assay was stopped by removing the supernatant and washing the beads with an ice-cold assay buffer (10 mM tris-HCl at pH 7.4, 75 mM NaCl, and 20 mM EDTA) supplemented with protease and phosphatase inhibitors. The bound fractions were eluted in 2× Laemmli buffer [4% SDS, 20% glycerol, 120 mM tris-HCl (pH 6.8), and 200 mM DTT].

### Protein digestion and MS

The protein content obtained from the on-bead phosphorylation assay was reduced with DTT, alkylated with iodoacetamide, and then digested overnight with trypsin, following a well-established protocol (*70*, *71*). The proteomic analysis was conducted on a Thermo Fisher Scientific Exploris 480 Orbitrap system coupled with a Dionex UltiMate 3000 RSLCnano system. After trapping and desalting, the tryptic peptides were loaded on an Aurora C18 column (75 mm by 250 mm, 1.6-μm particle size) nanocolumn (IonOpticks, Fitzroy, Australia) and separated using a linear gradient of acetonitrile in water (both added with 0.1% formic acid), from 3 to 41% in 1 hour, followed by column cleaning and reconditioning. The flow rate was set to 300 nl/min. Total run time was 1.5 hours. Injection volume was set to 1 μl. Peptides were analyzed in positive electrospray ionization mode, using a capillary voltage set to 2.0 kV. The radio frequency lens was set to 40%, and the AGC target was set to 300%. Data acquisition was performed in data-dependent mode. MS/MS spectra were acquired in HCD mode. All the collected MS/MS spectra were analyzed using the Proteome Discoverer software. Cysteine carbamidomethylation was selected as a fixed modification; acetylation of protein N terminus, methionine oxidation, and phosphorylation at serine, threonine, and tyrosine were selected as variable modifications. Positive protein identifications were retained at a 1% false discovery rate threshold and with at least two peptides matching the protein sequence. Unless otherwise specified, all reagents were purchased from Sigma-Aldrich and liquid chromatography–MS solvents from Thermo Fisher Scientific. All instruments and software were purchased from Thermo Fisher Scientific.

### Inhibition of REST degradation pathways

HEK293T cells at ∼70% confluency in 3.5-cm plates were transfected with 0.5 μg of either pRSV-CaMKIV (full-length; WT) or pRSV-CaMKIV (1 to 313; CA) using Lipofectamine 2000 (Thermo Fisher Scientific) and Dulbecco’s modified Eagle’s medium (DMEM; Thermo Fisher Scientific), supplemented with fetal bovine serum (FBS; 10%; Thermo Fisher Scientific) and penicillin-streptomycin (1%; Thermo Fisher Scientific), and were kept at 37°C in a 5% CO_2_ humidified atmosphere. One day after transfection, the medium was replaced with DMEM (Thermo Fisher Scientific) containing CHX (35 μM; Merck Life Science), 5% FBS, and 1% penicillin-streptomycin. Cells were kept at 37°C in a 5% CO_2_ humidified atmosphere. After 3 hours, cells were washed three times in DMEM/5% FBS/1% penicillin-streptomycin and incubated in the same medium containing vehicle (0.1% DMSO; Merck Life Science), MG-132 (5 μM; Merck Life Science), bafilomycin A1 (100 nM; Merck Life Science), leupeptin (40 μM; Merck Life Science), CA074 (10 μM; Tocris), and E64 (10 μM; Tocris). Following 16 hours of incubation at 37°C in a 5% CO_2_ humidified atmosphere, cells were washed three times in PBS and processed as described for Western blotting.

### Site-directed mutagenesis of REST phosphorylation sites

Nucleotides encoding Thr^294^ and Ser^322^ were mutated to either alanine or glutamic acid using the protocol of Dpn I–mediated site-directed mutagenesis (*72*). The primers used for the mutagenesis were the following:

*mRest*_TE_F: 5′-AGCACGTGCGAACTCACGAAGGAGAACGCCCGTAT-3′

*mRest*_TE_R: 5′-ATACGGGCGTTCTCCTTCGTGAGTTCGCACGTGCT-3′

*mRest*_SE F: 5′-GACACATGCGGACTCATGAAGGTGAGAAGCCAT-3′

*mRest*_SE R: 5′-ATGGCTTCTCACCTTCATGAGTCCGCATGTGTC-3′

*mRest*_TA_F: 5′-AGCACGTGCGAACTCACGCAGGAGAACGCCCGTAT-3′

*mRest*_TA_R: 5′-ATACGGGCGTTCTCCTGCGTGAGTTCGCACGTGCT-3′

*mRest*_SA F: 5′-GACACATGCGGACTCATGCAGGTGAGAAGCCAT-3′

*mRest*_SA_R: 5′-ATGGCTTCTCACCTGCATGAGTCCGCATGTGTC-3′

PCR amplification was performed on the template plasmid containing WT mREST cDNA in pEZ-Lv229-mREST-IRES2-mCherry constructs with the following protocol: 95°C for 30 s; 18 cycles of the following: 95°C for 30 s, 55°C for 1 min, and 68°C for 20 min using the pair of primers for each mutated site. The methylated template DNA contained in the PCR products was digested by incubation with 10 U of Dpn I for 2 hours at 37°C. A fraction (^1^/_50_) of the digested PCR product was used to transform TOP10 competent cells by heat shock at 42°C for 30 s, incubated in SOC medium for 30 min at 37°C, and then plated on an LB-agar plate with ampicillin (100 μg/ml). Minipreps from four colonies were prepared for full-length sequencing to identify the correct clone with mutated sites.

### Western blotting

Primary cortical neurons or HEK293T cells were used to obtain total cell lysates. Cells were collected in Lysis buffer [150 mM NaCl, 1% NP-40, 0.5% sodium deoxycholate, 0.1% SDS, 2 mM EDTA, and 50 mM tris-HCl (pH 7.4)] enriched with protease and phosphatase inhibitor cocktails (Merck Life Science, Milan, Italy). Following a 10-min incubation on ice, cell lysates were centrifuged at ×18,500*g* for 15 min at 4°C. The supernatant was collected and sonicated at 25% power for 5 s on ice. The BCA assay (Thermo Fisher Scientific) was used to determine protein concentration. Equivalent protein amounts were subjected to SDS–polyacrylamide gel electrophoresis on 7.5% polyacrylamide gels and blotted onto nitrocellulose membranes (Whatman, Maidstone, UK). Blotted membranes were blocked for 1 hour using 1% bovine serum albumin (Merck Life Science) in TBS [10 mM tris and 150 mM NaCl (pH 8.0)] together with 0.1% Triton X-100 and incubated overnight at 4°C with the following primary antibodies: anti-CaMKI (#ab68234, Abcam, Cambridge, UK), anti-CaMKII (#07-1496, Merck Life Science), anti-CaMKIV (#38 610275, BD Biosciences, Milano, Italy), anti-REST (#07-579, Merck Life Science), anti–glyceraldehyde-3-phosphate dehydrogenase (GAPDH; #2118, Cell Signaling/Euroclone, Milano, Italy), and anti-calnexin (#ADI-SPA-860, Enzo Life Sciences, Farmingdale, NY). Membranes were washed and incubated at RT for 1 hour with the following peroxidase-conjugated secondary antibodies: goat anti-mouse immunoglobulin G (IgG) (1:10,000; #32430, Thermo Fisher Scientific) and goat anti-rabbit IgG (1:5000; #32460, Thermo Fisher Scientific). Bands were detected using the enhanced chemiluminescence detection system (Thermo Fisher Scientific), acquired on the iBright Imaging System (Thermo Fisher Scientific), and immunoreactivity was quantified by densitometric analysis with ImageJ.

### Real-time qPCR

Total cellular RNA was extracted with TRIzol (Thermo Fisher Scientific), and RNA concentration was quantified using the NanoDrop 1000 spectrophotometer (Thermo Fisher Scientific). cDNA was synthesized starting from 0.25 μg of RNA with SuperScript IV Reverse Transcriptase kit (Thermo Fisher Scientific) according to the manufacturer’s instructions. Gene expression was measured by real-time qPCR using SsoAdvanced Universal SYBR Green Supermix (Bio-Rad Laboratories, Milano, Italy) on a C1000 Touch Thermal Cycler (Bio-Rad) on a CFX96 Real-Time System following the manufacturer’s protocol, with the following protocol: 5 min at 95°C, 10 s at 95°C, 20 s at 60°C, and 10 s at 72°C for 45 cycles; and melting curve (heating ramp from 55° to 95°C) to check for amplification specificity. The following primers (final concentration 0.25 μM) were used:

*Rest*_F: 5′-GAACCACCTCCCAGTATG-3′

*Rest*_R: 5′-CTTCTGACAATCCTCCATAG-3′

*Camk1*_F: 5′-ATCAAGGAAGTCAGGGTTT-3′

*Camk1*_R: 5′-GCAGTGAAGAGTGAGAGG-3′

*Camk2a*_F: 5′-ACTTCCTTCCACCACTTC-3′

*Camk2a*_R: 5′-TGAGATACAGCATTCCATACA-3′

*Camk4*_F: 5′-CAGTTCATGTTCAGGAGAAT-3′

*Camk4*_R: 5′-AATGTAGTCAGCCGTTTC-3′

*Gapdh*_F: 5′-AGGTCGGTGTGAACGGATTTG-3′

*Gapdh*_R: 5′-TGTAGACCATGTAGTTGAGGTCA-3′

*Syn1*_F: 5′-ACCTCAACAAATCCCAGTCT-3′

*Syn1*_R: 5′-CGGATGGTCTCAGCTTTCAC-3′

*Scn2a*_F: 5′-GGCTCTGCTGTCATTGTTG-3′

*Scn2a*_R: 5′-GAAGGCTAGGTGAGTACAT-3′

*Bdnf*_F: 5′-ATTACCTGGAATGCCGCAA-3′

*Bdnf*_R: 5′-TAATACTGTCACACACGCT-3′

*Snap25*_F: 5′-CCTAGTAGGTCTTGCACATCAC-3′

*Snap25*_R: 5′-GACAGAGCACACAGGACATT-3′

*Bsn*_F: 5′-CAGAGCAGAGGAGCCAAGG-3′

*Bsn*_R: 5′-CGAGGTGAATGGACGGTGTTA-3′

*Cplx1*_F: 5′-AAATAAGCCATAGTCCTAGATC-3′

*Cplx1*_R: 5′-GTCAGTGATGGCAGTATG-3′

*Npas4*_F: 5′-AGGGTTTGCTGATGAGTTGC-3′

*Npas4*_R: 5′-CCCCTCCACTTCCATCTTC-3′

Relative gene expression was determined using the 2^–ΔΔ*C*t^ method, with data normalized to the *Gapdh* housekeeping transcript (*73*).

### Immunocytochemistry and image analysis

HEK293T cells on 18-mm coverslips at 70% confluency were transfected with 0.5 μg of pEZ-Lv229-mREST-IRES2-mCherry WT (GeneCopoeia, Rockville, MD) or mutagenized constructs therefrom using Lipofectamine 2000 (Thermo Fisher Scientific). One day after transfection, cells were fixed in 4% paraformaldehyde in 0.1 M phosphate buffer, pH 7.4, for 20 min at RT. Fixed cells were stained with anti-REST antibody (#MABE1982, clone 12C11) and Hoechst to visualize the nuclear area. Briefly, cells were washed three times in PBS, permeabilized and blocked, and incubated with the primary antibody overnight at 4°C in 0.1% Triton X-100 and 5% normal goat serum in PBS. After four washes in high-salt PBS (20 mM Na_2_HPO_4_/20 mM NaH_2_PO_4_/0.5 M NaCl) for a total of 20 min, neurons were incubated in the same buffer with Alexa Fluor–conjugated secondary antibodies (1:500; Thermo Fisher Scientific) and counterstained with Hoechst 33342 for nuclear detection. Following 20-min wash in high-salt PBS, coverslips were washed once in PBS, once in double-distilled water, and mounted in Mowiol mounting medium. Tile-scan images of transfected HEK293T cells were acquired using a 60× objective with a Leica SP8 confocal microscope (Leica Microsystems, Wetzlar, Germany). The nuclear/cytosolic ratio of the REST immunoreactivity was calculated using Image Calculator in the Fiji distribution of ImageJ. Briefly, the nuclear REST immunoreactivity was defined as the REST signal that overlaps with the Hoechst signal, and the resulting image was used to subtract the nuclear REST signal from the total REST signal to obtain the cytosolic REST signal. The mean intensities in each panel were measured by ImageJ and were used to calculate the nuclear-to-cytosolic ratio. An alternative analysis of the colocalization of REST immunoreactivity with Hoechst (nucleus) or mCherry (cytoplasm) was performed on tile-scan images using the Colocalization Finder plugin for the Fiji distribution of ImageJ. Colocalization was computed between all channels, with focus on nuclear localization of anti-REST fluorescence (fig. S7). Pearson’s correlation coefficient was extracted as a measure of reciprocal channel colocalization, as previously described (*74*).

### Luciferase assay

HEK293T cells at 50% confluency in a six-well plate were cotransfected with RE1X3-pGL3 promoter vector (*30*), with mutagenized mREST in pEZ-Lv229-mREST-IRES2-mCherry, or with pRSV-CaMKIV (1 to 313) (#45063, Addgene, Watertown, MA) and the pRL TKSV40 vector expressing *RLuc* (Promega, Milano, Italy), using Lipofectamine 2000 (Thermo Fisher Scientific). Two days after transfection, cells were washed twice in PBS and collected in 1× Passive Lysis Buffer (PLB) from Dual-Luciferase Assay Kit (Promega), supplemented with protease inhibitor cocktail. The lysates were subjected to one free-thaw cycle and were centrifuged at ×100*g* at 4°C for 5 min to remove residual cell debris. The supernatants were collected, diluted 1:5 in 1× PLB, and added to multiwells containing the luciferase substrate LAR II. After a first basal reading of luminescence, a second reading was performed after the addition of Stop & Glo Reagent (Promega). All luminescence readings were obtained using a Spark Multimodal Microplate Reader (Tecan, Zurich, Switzerland).

### Generation of lentiviral vectors

VSV-pseudotyped third-generation lentiviruses were obtained as previously described (*75*). Briefly, lentiviruses were produced by transient four-plasmid cotransfection into HEK293T cells using the Ca^2+^ phosphate transfection method. Supernatants were collected, passed through a 0.45-μm filter, and purified by ultracentrifugation (×75,000*g* for 2 hours at 4°C). Viral vectors were titrated at concentrations ranging from 1 × 10^8^ to 1 × 10^9^ transducing units/ml and used at a multiplicity of infection (MOI) of 1 to 10.

#### RNA interference for CaMK knockdown

For specific knockdown of CaMKs in neurons, shRNA was cloned by inserting the annealed oligos of shRNA for either CaMKI or CaMKIV into the pLenti-U6-shRNA-PGK-GFP vector between the Xho I and Hpa I sites. The shRNA oligonucleotides used were as follows:

shCamk1a sense oligo III: 5′-TGCATTGTAGCCCTGGATGACTTCAAGAGA GTCATCCAGGGCTACAATGCTTTTTTC-3′

shCamk1a antisense oligo III: 5′-CTCGAGAAAAAAGCATTGTAGCCCTGGATGA CTCTCTTGAAGTCATCCAGGGCTACAATGCA-3′

shCamk1a sense oligo IV: 5′-TGGATCAAGCACCCCAACATTTTCAAGAGA AATGTTGGGGTGCTTGATCCTTTTTTC-3′

shCamk1a antisense oligo IV: 5′-CTCGAGAAAAAAGGATCAAGCACCCCAACATT TCTCTTGAAAATGTTGGGGTGCTTGATCCA-3′

shCamk4 sense oligo II: 5′-TGAGAGAATCTTCTTTATGCATTCAAGAGA TGCATAAAGAAGATTCTCTCTTTTTTC-3′

shCamk4 antisense oligo II: 5′-CTCGAGAAAAAAGAGAGAATCTTCTTTATGCA TCTCTTGAATGCATAAAGAAGATTCTCTCA-3′

shCamk4 sense oligo III: 5′-TGGTGTTAAAGAAAACAGTGGTTCAAGAGA CCACTGTTTTCTTTAACACCTTTTTTC-3′

shCamk4 antisense oligo III: 5′-CTCGAGAAAAAAGGTGTTAAAGAAAACAGTGG TCTCTTGAACCACTGTTTTCTTTAACACCA-3′

scramble sense oligo: 5′-TGAGAGAATCTTCTTTATGCATTCAAGAGA TTGGGTTGAAGGTGGATCCCTTTTTTC-3′

scramble antisense oligo: 5′-CTCGAGAAAAAAGGGATCCACCTTCAACCCAA TCTCTTGAATGCATAAAGAAGATTCTC-TCA-3′

#### RNA interference for REST knockdown

*pLV[shRNA]-mCherry-U6 > {shmREST_2017}*, which contains the shRNA sequence for REST (*11*, *12*) under the U6 promoter with a reporter mCherry under the phosphoglycerate kinase-1 (PGK) ubiquitous promoter, and the scrambled version thereof *pLV[shRNA]-mCherry-U6 > {Scramble_shRNA}* were manufactured and purchased from VectorBuilder Inc. (Chicago, Illinois, USA). The sh sequences are as follows:

shREST: ACATGCAAGACAGGTTCACAA*CTCGAG*TTGTGAACCTGTCTTGCATGT

Scrambled (Scr): CCTAAGGTTAAGTCGCCCTCG*CTCGAG*CGAGGGCGACTTAACCTTAGG

#### Expression of catalytically active CaMKIV

*pLV[Exp]-hPGK > HA/{CamKIV ca_1*–*313aa}: IRES:{NLS-EGFP-DeltaCre}* was purchased from VectorBuilder Inc. (Chicago, Illinois, USA) with the cDNA insert that encodes 1 to 313 amino acids of CaMKIV as in the pRSV-CaMK4 CA (1 to 313) (#45063; Addgene, Watertown, MA), under the promoter of hPGK with an internal ribosomal entry site (IRES) sequence, followed by EGFP-DeltaCre with nuclear localization signal.

### Transduction of primary neurons

WT primary cortical neurons at DIV7 TO DIV9 were transduced with the respective lentiviral vectors, which also encode the soluble reporters of transduction (see above). To express WT REST or its phosphorylation mutants: TA, SA, and TASA nonphosphorylatable mutants and TE, SE, and TESE phosphomimic mutants, neurons were infected at an MOI of 1. To combine CaMKIV modulation with REST silencing, WT primary neurons were cotransduced with shREST/mCherry (*11*, *12*) and/or shCaMKIV or catalytically active CaMKIV/green fluorescent protein (GFP), with the scrambled or empty versions of the respective viruses serving as controls at an MOI of 10. The infection efficiency was estimated to range from 70 to 90% by counting GFP-/mCherry-expressing neurons relative to the total number of cells. To trigger homeostatic plasticity responses, cortical neurons at DIV12 to DIV14 were treated for 48 hours with either 4AP (100 μM) or an equivalent amount of vehicle (water). Experiments were performed 7 days after infection at DIV14 to DIV16.

The knockdown of CaMKIV was also associated with full REST KO, using primary cortical neurons from RestGTi cKO mice that were alternatively transduced with sequences containing active or inactive Cre recombinase (Cre and its deletion mutant ΔCre), cloned into pGFP-Lenti using the PGK ubiquitous promoter (*75*). cKO cortical neurons were infected as described above at an MOI of 10. Experiments were performed 7 days after infection.

### Structural modeling of the REST:RE1 complex

#### Atomistic MD simulations of the WT REST-RE1 complex

Structures of the WT REST–RE1 assembly were generated as described in the Supplementary Materials (the “Molecular modeling of wild-type REST” section in Supplementary Methods and following). The CHARMM-GUI solution builder plug-in (*76*, *77*) was used to set up the system and generate input files for all-atom MD simulations. The DNA-protein assembly was inserted into a rectangular box of TIP3 water molecules (*78*) with a minimum solute–to–box-edge distance of 15 Å, applied along all directions. The total system charge was neutralized by adding Na^+^ and Cl^−^ ions, reaching a physiological concentration of 0.15 M. The whole system amounts to ∽112,000 atoms. Simulations were performed with NAMD 2.14 (*79*), using the Amber ff14SB (*80*) force field for the protein and the BSC1 (*81*) force field for the nucleic acid. Tetragonal periodic boundary conditions (*82*) were applied. The particle mesh Ewald method (*83*) was used to treat electrostatic interactions and to avoid box-surface effects, with a maximum grid spacing of 1 Å and sixth-order beta splines. A cutoff of 12 Å was used for Lennard-Jones interactions, which were smoothly switched off starting at 10 Å. Chemical bond distances involving hydrogen atoms were constrained using the SHAKE algorithm (*84*). A time step of 2 fs was used. Before entering the production stage, the system was energy minimized and equilibrated in the isobaric-isothermal ensemble (NPT) at *P* = 1 atm, and *T* = 310 K. Pressure and temperature were maintained constant using the Nose-Hoover Langevin piston (*85*) and the Langevin thermostat (*86*), respectively. Afterwards, three independent, 1-μs-long unbiased all-atom MD simulations were performed in the NPT ensemble.

#### Clustering of WT REST-RE1

To generate phosphomimic mutant conformations, we extracted snapshots from the equilibrium simulations of WT REST-RE1. Trajectories were individually subjected to clustering analysis using TTClust4.10.3 (*87*). MD frames were aligned using the backbone of both the protein and the DNA as a reference. Pairwise RMSDs were assembled into as matrix and used for hierarchical clustering with the Ward algorithm (*88*) to build the linkage matrix, minimizing the increase in within-cluster variance. Structures of cluster centers were visually inspected using the PyMOL Molecular Graphics System [2002, version 2.0, Schrödinger LLC, New York, NY; (*89*)], and, for each replica, the center of the most populated cluster was taken as the representative conformation of that MD run.

#### Atomistic MD simulations of REST phosphomimics

Two distinct phosphomimic mutants were generated by varying the amino acid identity of positions T154 and S192 (T294 and S322 in the experimental system, respectively) into glutamic acid. All simulation procedures and parameters were the same as for the WT system. A total of 18 independent, 1-μs-long, unbiased all-atom MD simulations, 9 per mutated system, was performed in the NPT ensemble.

### Patch-clamp electrophysiology

Whole-cell patch-clamp recordings were performed on low-density cortical neurons at RT at DIV14 to DIV16 (*66*–*68*). Patch pipettes were pulled (Narishige PC-10 puller) and fire polished from thin-borosilicate glass (Kimble Chase, #34500-99) to a final resistance of 3 to 4 MΩ when filled with a standard internal solution. Recordings were performed using an EPC-800 amplifier and LIH 8 + 8 digitizer (HEKA Elektronik). Recordings with leak currents > 200 pA or series resistance > 20 MΩ were discarded. For all the experiments, neurons were maintained in standard extracellular Tyrode solution containing 140 mM NaCl, 2 mM CaCl_2_, 1 mM MgCl_2_, 4 mM KCl, 10 mM glucose, and 10 mM Hepes (pH 7.3 with NaOH). This solution was supplemented with tetrodotoxin (0.3 μM; #1078, Tocris) to block voltage-gated sodium channels and the generation and propagation of spontaneous APs; bicuculline methiodide (30 μM; #2503, Tocris) to block γ-aminobutyric acid type A (GABA_A_) receptors; [(2*S*)-3-[(1*S*)-1-(3,4-dichlorophenyl)ethyl]amino-2-hydroxypropyl] (phenyl methyl)phosphinic acid hydrochloride (CGP; 5 μM; #1469, Tocris) to block GABA_B_ receptors; and d-(−)-2-amino-5-phosphonopentanoic acid (50 μM; #0106, Tocris) to block *N*-methyl-d-aspartate receptors. The internal solution (k-gluconate) used for recording mEPSCs in voltage-clamp configuration contained 126 mM k-gluconate, 4 mM NaCl, 1 mM MgSO_4_, 0.02 mM CaCl_2_, 0.1 mM 1,2-bis(2-aminophenoxy)ethane-*N*,*N*,*N*′,*N*′-tetraacetic acid, 15 mM glucose, 5 mM Hepes, 3 mM ATP, and 0.1 mM guanosine 5′-triphosphate (pH 7.3 with KOH). mEPSCs were acquired at a holding potential of −70 mV, sampled at 10 kHz, and filtered at one-fifth the acquisition rate with a low-pass Bessel filter. The amplitude and frequency of the miniature excitatory events were calculated with a peak-detection function in Mini Analysis (Synaptosoft), using appropriate threshold amplitudes and areas.

### Statistical analysis

Data are expressed as means ± SEM with individual experimental replications, as detailed in the figure legends. No exclusion criteria were preestablished. Normal distribution of data was assessed using the D’Agostino-Pearson’s normality test (*n* > 6) or the Shapiro-Wilk test (*n* < 6). The *F*-test was used to compare the variance between the two sample groups. The two-tailed unpaired Student’s *t* test was used to compare two normally distributed experimental groups. One-way or two-way analysis of variance (ANOVA), followed by Tukey’s, Holm-Šídák, or Dunnett’s multiple-comparison test, was used to compare more than two normally distributed experimental groups. The Kruskal-Wallis ANOVA was used to compare more than two nonnormally distributed experimental groups, followed by Dunn’s multiple-comparison test. The significance level was preset to *P* < 0.05. Statistical analysis was done using Prism (GraphPad Software Inc.).

## Supplementary Materials

### This PDF file includes:

Supplementary Methods Tables S1 and S2 Figs. S1 to S20 References

## References

[1] A. W. Bruce, I. J. Donaldson, I. C. Wood, S. A. Yerbury, M. I. Sadowski, M. Chapman, B. Göttgens, N. J. Buckley, Genome-wide analysis of repressor element 1 silencing transcription factor/neuron-restrictive silencing factor (REST/NRSF) target genes. Proc. Natl. Acad. Sci. U.S.A. 101, 10458–10463 (2004). 10.1073/pnas.0401827101 PMC478591 15240883

[2] N. Ballas, G. Mandel, The many faces of REST oversee epigenetic programming of neuronal genes. Curr. Opin. Neurobiol. 15, 500–506 (2005). 10.1016/j.conb.2005.08.015 16150588

[3] N. Mori, C. Schoenherr, D. J. Vandenbergh, D. J. Anderson, A common silencer element in the SCG10 and type II Na^+^ channel genes binds a factor present in nonneuronal cells but not in neuronal cells. Neuron 9, 45–54 (1992). 10.1016/0896-6273(92)90219-4 1321646

[4] S. D. Kraner, J. A. Chong, H. J. Tsay, G. Mandel, Silencing the type II sodium channel gene: A model for neural-specific gene regulation. Neuron 9, 37–44 (1992). 10.1016/0896-6273(92)90218-3 1321645

[5] N. Ballas, C. Grunseich, D. D. Lu, J. C. Speh, G. Mandel, REST and its corepressors mediate plasticity of neuronal gene chromatin throughout neurogenesis. Cell 121, 645–657 (2005). 10.1016/j.cell.2005.03.013 15907476

[6] M. Yeo, K. Berglund, G. Augustine, W. Liedtke, Novel repression of Kcc2 transcription by REST-RE-1 controls developmental switch in neuronal chloride. J. Neurosci. 29, 14652–14662 (2009). 10.1523/JNEUROSCI.2934-09.2009 PMC2833346 19923298

[7] A. Rodenas-Ruano, A. E. Chávez, M. J. Cossio, P. E. Castill, R. S. Zukin, REST-dependent epigenetic remodeling promotes the developmental switch in synaptic NMDA receptors. Nat. Neurosci. 15, 1382–1390 (2012). 10.1038/nn.3214 PMC3501125 22960932

[8] H. Aoki, A. Hara, T. Era, T. Kunisada, Y. Yamada, Genetic ablation of Rest leads to in vitro-specific derepression of neuronal genes during neurogenesis. Development 139, 667–677 (2012). 10.1242/dev.072272 22241837

[9] P. Baldelli, J. Meldolesi, The transcription repressor REST in adult neurons: Physiology, pathology, and diseases. eNeuro 2, ENEURO.0010-15.2015 (2015). 10.1523/ENEURO.0010-15.2015 PMC4596026 26465007

[10] C. Michetti, F. Benfenati, Homeostatic regulation of brain activity: From endogenous mechanisms to homeostatic nanomachines. Am. J. Physiol. Cell Physiol. 327, C1384–C1399 (2024). 10.1152/ajpcell.00470.2024 39401424

[11] D. Pozzi, G. Lignani, E. Ferrea, A. Contestabile, F. Paonessa, R. D’Alessandro, P. Lippiello, D. Boido, A. Fassio, J. Meldolesi, F. Valtorta, F. Benfenati, P. Baldelli, REST/NRSF-mediated intrinsic homeostasis protects neuronal networks from hyperexcitability. EMBO J. 32, 2994–3007 (2013). 10.1038/emboj.2013.231 PMC3831314 24149584

[12] F. Pecoraro-Bisogni, G. Lignani, A. Contestabile, E. Castroflorio, D. Pozzi, A. Rocchi, C. Prestigio, M. Orlando, P. Valente, M. Massacesi, F. Benfenati, P. Baldelli, REST-dependent presynaptic homeostasis induced by chronic neuronal hyperactivity. Mol. Neurobiol. 55, 4959–4972 (2018). 10.1007/s12035-017-0698-9 28786015

[13] C. Prestigio, D. Ferrante, A. Marte, A. Romei, G. Lignani, F. Onofri, P. Valente, F. Benfenati, P. Baldelli, REST/NRSF drives homeostatic plasticity of inhibitory synapses in a target-dependent fashion. eLife 10, e69058 (2021). 10.7554/eLife.69058 PMC8639147 34855580

[14] A. Singh-Taylor, J. Molet, S. Jiang, A. Korosi, J. L. Bolton, Y. Noam, K. Simeone, J. Cope, Y. Chen, A. Mortazavi, T. Z. Baram, NRSF-dependent epigenetic mechanisms contribute to programming of stress-sensitive neurons by neonatal experience, promoting resilience. Mol. Psychiatry 23, 648–657 (2018). 10.1038/mp.2016.240 PMC5503824 28070121

[15] M. Mampay, G. K. Sheridan, REST: An epigenetic regulator of neuronal stress responses in the young and ageing brain. Front. Neuroendocrinol. 53, 100744 (2019). 10.1016/j.yfrne.2019.04.001 31004616

[16] J. L. Bolton, A. Schulmann, M. M. Garcia-Curran, L. Regev, Y. Chen, N. Kamei, M. Shao, A. Singh-Taylor, S. Jiang, Y. Noam, J. Molet, A. Mortazavi, T. Z. Baram, Unexpected transcriptional programs contribute to hippocampal memory deficits and neuronal stunting after early-life adversity. Cell Rep. 33, 108511 (2020). 10.1016/j.celrep.2020.108511 PMC7817243 33326786

[17] T. Lu, L. Aron, J. Zullo, Y. Pan, H. Kim, Y. Chen, T. H. Yang, H. M. Kim, D. Drake, X. S. Liu, D. A. Bennett, M. P. Colaiácovo, B. A. Yankner, REST and stress resistance in ageing and Alzheimer’s disease. Nature 507, 448–454 (2014). 10.1038/nature13163 PMC4110979 24670762

[18] J. M. Zullo, D. Drake, L. Aron, P. O’Hern, S. C. Dhamne, N. Davidsohn, C. A. Mao, W. H. Klein, A. Rotenberg, D. A. Bennett, G. M. Church, M. P. Colaiácovo, B. A. Yankner, Regulation of lifespan by neural excitation and REST. Nature 574, 359–364 (2019). 10.1038/s41586-019-1647-8 PMC6893853 31619788

[19] A. Rocchi, E. Carminati, A. De Fusco, J. A. Kowalska, T. Floss, F. Benfenati, REST/NRSF deficiency impairs autophagy and leads to cellular senescence in neurons. Aging Cell 20, e13471 (2021). 10.1111/acel.13471 PMC8520714 34520100

[20] E. Centonze, A. Marte, M. Albini, A. Rocchi, F. Cesca, M. Chiacchiaretta, T. Floss, P. Baldelli, S. Ferroni, F. Benfenati, P. Valente, Neuron-restrictive silencer factor/repressor element 1-silencing transcription factor (NRSF/REST) controls spatial K^+^ buffering in primary cortical astrocytes. J. Neurochem. 165, 701–721 (2023). 10.1111/jnc.15755 36636908

[21] N. J. Buckley, R. Johnson, C. Zuccato, A. Bithell, E. Cattaneo, The role of REST in transcriptional and epigenetic dysregulation in Huntington’s disease. Neurobiol. Dis. 39, 28–39 (2010). 10.1016/j.nbd.2010.02.003 20170730

[22] S. Mcclelland, C. Flynn, C. Dubé, C. Richichi, Q. Zha, A. Ghestem, M. Esclapez, C. Bernard, T. Z. Baram, Neuron-restrictive silencer factor-mediated hyperpolarization-activated cyclic nucleotide gated channelopathy in experimental temporal lobe epilepsy. Ann. Neurol. 70, 454–465 (2011). 10.1002/ana.22479 PMC3177145 21905079

[23] S. Mcclelland, G. P. Brennan, C. Dubé, S. Rajpara, S. Iyer, C. Richichi, C. Bernard, T. Z. Baram, The transcription factor NRSF contributes to epileptogenesis by selective repression of a subset of target genes. eLife 3, e01267 (2014). 10.7554/eLife.01267 PMC4129437 25117540

[24] A. M. Hall, N. Kamei, M. Shao, H.-S. Mun, K. Chen, Y. Chen, T. Z. Baram, Inhibition of Neuron-Restrictive Silencing Factor (REST/NRSF) chromatin binding attenuates epileptogenesise. Neuro 11, ENEURO.0006-24.2024 (2024). 10.1523/ENEURO.0006-24.2024 PMC11103648 38641413

[25] E. Carminati, F. Buffolo, A. Rocchi, C. Michetti, F. Cesca, F. Benfenati, Mild inactivation of RE-1 silencing transcription factor (REST) reduces susceptibility to kainic acid-induced seizures. Front. Cell. Neurosci. 13, 580 (2020). 10.3389/fncel.2019.00580 PMC6965066 31998079

[26] F. Buffolo, V. Petrosino, M. Albini, M. Moschetta, F. Carlini, T. Floss, N. K. De Rosbo, F. Cesca, A. Rocchi, A. Uccelli, F. Benfenati, Neuroinflammation induces synaptic scaling through IL-1β-mediated activation of the transcriptional repressor REST/NRSF. Cell Death Dis. 12, 180 (2021). 10.1038/s41419-021-03465-6 PMC7884694 33589593

[27] G. Natali, C. Michetti, A. Krawczun-Rygmaczewska, T. Floss, F. Cesca, F. Benfenati, Conditional knockout of REST/NRSF in excitatory neurons reduces seizure susceptibility to chemical kindling. Front. Cell. Neurosci. 17, 1267609 (2023). 10.3389/fncel.2023.1267609 PMC10687554 38034589

[28] J.-Y. Hwang, R. S. Zukin, REST, a master transcriptional regulator in neurodegenerative disease. Curr. Opin. Neurobiol. 48, 193–200 (2018). 10.1016/j.conb.2017.12.008 PMC5892838 29351877

[29] M. Shimojo, L. B. Hersh, Characterization of the REST/NRSF-interacting LIM domain protein (RILP): Localization and interaction with REST/NRSF. J. Neurochem. 96, 1130–1138 (2006). 10.1111/j.1471-4159.2005.03608.x 16417580

[30] F. Paonessa, S. Criscuolo, S. Sacchetti, D. Amoroso, H. Scarongella, F. Pecoraro Bisogni, E. Carminati, G. Pruzzo, L. Maragliano, F. Cesca, F. Benfenati, Regulation of neural gene transcription by optogenetic inhibition of the RE1-silencing transcription factor. Proc. Natl. Acad. Sci. U.S.A. 113, E91–E100 (2015). 10.1073/pnas.1507355112 PMC4711829 26699507

[31] K. Palm, N. Belluardo, M. Metsis, T. Õ. Timmusk, Neuronal expression of zinc finger transcription factor REST/NRSF/XBR gene. J. Neurosci. 18, 1280–1296 (1998). 10.1523/JNEUROSCI.18-04-01280.1998 PMC6792720 9454838

[32] S. Rockowitz, W.-H. Lien, E. Pedrosa, G. Wei, M. Lin, K. Zhao, H. M. Lachman, E. Fuchs, D. Zheng, Comparison of REST cistromes across human cell types reveals common and context-specific functions. PLoS Comput. Biol. 10, e1003671 (2014). 10.1371/journal.pcbi.1003671 PMC4055426 24922058

[33] C. Brenner, R. Deplus, C. Didelot, A. Loriot, E. Viré, C. De Smet, A. Gutierrez, D. Danovi, D. Bernard, T. Boon, P. Giuseppe Pelicci, B. Amati, T. Kouzarides, Y. De Launoit, L. Di Croce, F. Fuks, Myc represses transcription through recruitment of DNA methyltransferase corepressor. EMBO J. 24, 336–346 (2005). 10.1038/sj.emboj.7600509 PMC545804 15616584

[34] W. J. Friesen, M. K. Darby, Phage display of RNA binding zinc fingers from transcription factor IIIA. J. Mol. Biol. 272, 10994–10997 (1997). 10.1074/jbc.272.17.10994 9110988

[35] A. Klug, The discovery of zinc fingers and their development for practical applications in gene regulation and genome manipulation. Q. Rev. Biophys. 43, 1–21 (2010). 10.1017/S0033583510000089 20478078

[36] T. F. Westbrook, G. Hu, X. L. Ang, P. Mulligan, N. N. Pavlova, A. Liang, Y. Leng, R. Maehr, Y. Shi, J. W. Harper, S. J. Elledge, SCFβ-TRCP controls oncogenic transformation and neural differentiation through REST degradation. Nature 452, 370–374 (2008). 10.1038/nature06780 PMC2688689 18354483

[37] D. Guardavaccaro, D. Frescas, N. V. Dorrello, A. Peschiaroli, A. S. Multani, T. Cardozo, A. Lasorella, A. Iavarone, S. Chang, E. Hernando, M. Pagano, Control of chromosome stability by the β-TrCP–REST–Mad2 axis. Nature 452, 365–369 (2008). 10.1038/nature06641 PMC2707768 18354482

[38] N. Kaneko, J.-Y. Hwang, M. Gertner, F. Pontarelli, R. S. Zukin, Casein kinase 1 suppresses activation of REST in insulted hippocampal neurons and halts ischemia-induced neuronal death. J. Neurosci. 34, 6030–6039 (2014). 10.1523/JNEUROSCI.4045-13.2014 PMC3996220 24760862

[39] E. Nesti, G. M. Corson, M. Mccleskey, J. A. Oyer, G. Mandel, C-terminal domain small phosphatase 1 and MAP kinase reciprocally control REST stability and neuronal differentiation. Proc. Natl. Acad. Sci. U.S.A. 111, E3929–E3936 (2014). 10.1073/pnas.1414770111 PMC4169941 25197063

[40] G. G. Turrigiano, The self-tuning neuron: Synaptic scaling of excitatory synapses. Cell 135, 422–435 (2008). 10.1016/j.cell.2008.10.008 PMC2834419 18984155

[41] G. Turrigiano, Too many cooks? Intrinsic and synaptic homeostatic mechanisms in cortical circuit refinement. Annu. Rev. Neurosci. 34, 89–103 (2011). 10.1146/annurev-neuro-060909-153238 21438687

[42] N. Ageta-Ishihara, S. Takemoto-Kimura, M. Nonaka, A. Adachi-Morishima, K. Suzuki, S. Kamijo, H. Fujii, T. Mano, F. Blaeser, T. A. Chatila, H. Mizuno, T. Hirano, Y. Tagawa, H. Okuno, H. Bito, Control of cortical axon elongation by a GABA-driven Ca^2+^/calmodulin-dependent protein kinase cascade. J. Neurosci. 29, 13720–13729 (2009). 10.1523/JNEUROSCI.3018-09.2009 PMC2796271 19864584

[43] D. C. Bersten, J. A. Wright, P. J. Mccarthy, M. L. Whitelaw, Regulation of the neuronal transcription factor NPAS4 by REST and microRNAs. Biochim. Biophys. Acta 1839, 13–24 (2013). 10.1016/j.bbagrm.2013.11.004 24291638

[44] H. Xu, L. Yong, X. Gao, Y. Chen, Y. Wang, F. Wang, X. Hou, CaMK4: Structure, physiological functions, and therapeutic potential. Biochem. Pharmacol. 224, 116204 (2024). 10.1016/j.bcp.2024.116204 38615920

[45] J. Kaiser, A. Risteska, A. G. Muller, H. Sun, B. Lei, K. Nay, A. R. Means, M. A. Cousin, D. H. Drewry, J. S. Oakhill, B. E. Kemp, A. J. Hannan, M. Berk, M. A. Febbraio, A. L. Gundlach, E. L. Hill-Yardin, J. W. Scott, Convergence on CaMK4: A key modulator of autism-associated signaling pathways in neurons. Biol. Psychiatry 97, 439–449 (2025). 10.1016/j.biopsych.2024.10.012 39442785

[46] S. M. Lemrow, K. A. Anderson, J. D. Joseph, T. J. Ribar, P. K. Noeldner, A. R. Means, Catalytic activity is required for calcium/calmodulin-dependent protein kinase IV to enter the nucleus. J. Biol. Chem. 279, 11664–11671 (2004). 10.1074/jbc.M312613200 14701808

[47] R. P. Matthews, C. R. Guthrie, L. M. Wailes, X. Zhao, A. R. Means, G. S. Mcknight, Calcium/calmodulin-dependent protein kinase types II and IV differentially regulate CREB-dependent gene expression. Mol. Cell. Biol. 14, 6107–6116 (1994). 10.1128/mcb.14.9.6107-6116.1994 PMC359137 8065343

[48] R. R. White, Y.-G. Kwon, M. Taing, D. S. Lawrence, A. M. Edelman, Definition of optimal substrate recognition motifs of Ca^2+^-calmodulin-dependent protein kinases IV and II reveals shared and distinctive features. J. Biol. Chem. 273, 3166–3172 (1998). 10.1074/jbc.273.6.3166 9452427

[49] D. Jantz, J. M. Berg, Reduction in DNA-binding affinity of Cys_2_His^2^ zinc finger proteins by linker phosphorylation. Proc. Natl. Acad. Sci. U.S.A. 101, 7589–7593 (2004). 10.1073/pnas.0402191101 PMC419650 15128941

[50] A. Joseph, G. G. Turrigiano, All for one but not one for all: Excitatory synaptic scaling and intrinsic excitability are coregulated by CaMKIV, whereas inhibitory synaptic scaling is under independent control. J. Neurosci. 37, 6778–6785 (2017). 10.1523/JNEUROSCI.0618-17.2017 PMC5508258 28592691

[51] Y. Qiu, N. O’Neill, B. Maffei, C. Zourray, A. Almacellas-Barbanoj, J. C. Carpenter, S. P. Jones, M. Leite, T. J. Turner, F. C. Moreira, On-demand cell-autonomous gene therapy for brain circuit disorders. Science 378, 523–532 (2022). 10.1126/science.abq6656 PMC7613996 36378958

[52] J. C. Lee, Y. G. Kwon, D. S. Lawrence, A. M. Edelman, A requirement of hydrophobic and basic amino acid residues for substrate recognition by Ca2+/calmodulin-dependent protein kinase Ia. Proc. Natl. Acad. Sci. U.S.A. 91, 6413–6417 (1994). 10.1073/pnas.91.14.6413 PMC44212 8022798

[53] S. Dale, W. A. Wilson, A. M. Edelman, D. G. Hardie, Similar substrate recognition motifs for mammalian AMP-activated protein kinase, higher plant HMG-CoA reductase kinase-A, yeast SNF1, and mammalian calmodulin-dependent protein kinase I. FEBS Lett. 361, 191–195 (1995). 10.1016/0014-5793(95)00172-6 7698321

[54] S. Dovat, T. Ronni, D. Russell, R. Ferrini, B. S. Cobb, S. T. Smale, A common mechanism for mitotic inactivation of C2H2 zinc finger DNA-binding domains. Genes Dev. 16, 2985–2990 (2002). 10.1101/gad.1040502 PMC187490 12464629

[55] R. Rizkallah, K. E. Alexander, M. M. Hurt, Global mitotic phosphorylation of C2H2zinc finger protein linker peptides. Cell Cycle 10, 3327–3336 (2011). 10.4161/cc.10.19.17619 PMC3233627 21941085

[56] R. Conesa-Bakkali, M. Morillo-Huesca, J. Martínez-Fábregas, Non-canonical, extralysosomal activities of lysosomal peptidases in physiological and pathological conditions: New clinical opportunities for cancer therapy. Cells 14, 68 (2025). 10.3390/cells14020068 PMC11763575 39851495

[57] M. C. Yoon, M. P. Christy, V. V. Phan, W. H. Gerwick, G. Hook, A. J. O’Donoghue, V. Hook, Molecular features of CA-074 pH-dependent inhibition of cathepsin B. Biochemistry 61, 228–238 (2022). 10.1021/acs.biochem.1c00684 PMC9096814 35119840

[58] A. M. Cuervo, J. F. Dice, E. Knecht, A population of rat liver lysosomes responsible for the selective uptake and degradation of cytosolic proteins. J. Biol. Chem. 272, 5606–5615 (1997). 10.1074/jbc.272.9.5606 9038169

[59] S. J. Myers, J. Peters, Y. Huang, M. B. Comer, F. Barthel, R. Dingledine, Transcriptional regulation of the GluR2 gene: Neural-specific expression, multiple promoters, and regulatory elements. J. Neurosci. 18, 6723–6739 (1998). 10.1523/JNEUROSCI.18-17-06723.1998 PMC6792970 9712644

[60] A. Calderone, T. Jover, K. Noh, H. Tanaka, H. Yokota, Y. Lin, S. Y. Grooms, R. Regis, M. V. L. Bennett, R. S. Zukin, Ischemic insults derepress the gene silencer REST in neurons destined to die. J. Neurosci. 23, 2112–2121 (2003). 10.1523/JNEUROSCI.23-06-02112.2003 PMC6741998 12657670

[61] K.-M. Noh, J.-Y. Hwang, A. Follenzi, R. Athanasiadou, T. Miyawaki, J. M. Greally, M. V. L. Bennett, R. S. Zukin, Repressor element-1 silencing transcription factor (REST)-dependent epigenetic remodeling is critical to ischemia-induced neuronal death. Proc. Natl. Acad. Sci. U.S.A. 109, E962–E971 (2012). 10.1073/pnas.1121568109 PMC3341013 22371606

[62] P. Kallunki, G. M. Edelman, F. S. Jones, The neural restrictive silencer element can act as both a repressor and enhancer of L1 cell adhesion molecule gene expression during postnatal development. Proc. Natl. Acad. Sci. U.S.A. 95, 3233–3238 (1998). 10.1073/pnas.95.6.3233 PMC19725 9501246

[63] A. Perera, D. Eisen, M. Wagner, S. K. Laube, A. F. Künzel, S. Koch, J. Steinbacher, E. Schulze, V. Splith, N. Mittermeier, TET3 is recruited by REST for context-specific hydroxymethylation and induction of gene expression. Cell Rep. 11, 283–294 (2015). 10.1016/j.celrep.2015.03.020 25843715

[64] E. Pajarillo, A. Digman, I. Nyarko-Danquah, D.-S. Son, K. F. A. Soliman, M. Aschner, E. Lee, Astrocytic transcription factor REST upregulates glutamate transporter EAAT2, protecting dopaminergic neurons from manganese-induced excitotoxicity. J. Biol. Chem. 297, 101372 (2021). 10.1016/j.jbc.2021.101372 PMC8626589 34756885

[65] T. Nechiporuk, J. McGann, K. Mullendorff, J. Hsieh, W. Wurst, T. Floss, G. Mandel, The REST remodeling complex protects genomic integrity during embryonic neurogenesis. eLife 5, e09584 (2016). 10.7554/eLife.09584 PMC4728133 26745185

[66] P. Baldelli, A. Fassio, F. Valtorta, F. Benfenati, Lack of synapsin I reduces the readily releasable pool of synaptic vesicles at central inhibitory synapses. J. Neurosci. 27, 13520–13531 (2007). 10.1523/JNEUROSCI.3151-07.2007 PMC6673103 18057210

[67] M. Chiappalone, S. Casagrande, M. Tedesco, F. Valtorta, P. Baldelli, S. Martinoia, F. Benfenati, Opposite changes in glutamatergic and GABAergic transmission underlie the diffuse hyperexcitability of synapsin I-deficient cortical networks. Cereb. Cortex 19, 1422–1439 (2009). 10.1093/cercor/bhn182 19020204

[68] P. Valente, E. Castroflorio, P. Rossi, M. Fadda, B. Sterlini, R. I. Cervigni, C. Prestigio, S. Giovedì, F. Onofri, E. Mura, F. C. Guarnieri, A. Marte, M. Orlando, F. Zara, A. Fassio, F. Valtorta, P. Baldelli, A. Corradi, F. Benfenati, PRRT2 is a key component of the Ca^2+^-dependent neurotransmitter release machinery. Cell Rep. 15, 117–131 (2016). 10.1016/j.celrep.2016.03.005 PMC4826441 27052163

[69] P. Sun, H. Enslen, P. S. Myung, R. A. Maurer, Differential activation of CREB by Ca^2+^/calmodulin-dependent protein kinases type II and type IV involves phosphorylation of a site that negatively regulates activity. Genes Dev. 8, 2527–2539 (1994). 10.1101/gad.8.21.2527 7958915

[70] V. Castagnola, V. Tomati, L. Boselli, C. Braccia, S. Decherchi, P. P. Pompa, N. Pedemonte, F. Benfenati, A. Armirotti, Sources of biases in the in vitro testing of nanomaterials: The role of the biomolecular corona. Nanoscale Horiz. 9, 799–816 (2024). 10.1039/d3nh00510k 38563642

[71] N. Liessi, L. Maragliano, V. Castagnola, M. Bramini, F. Benfenati, A. Armirotti, Isobaric labeling proteomics allows a high-throughput investigation of protein corona orientation. Anal. Chem. 93, 784–791 (2021). 10.1021/acs.analchem.0c03134 PMC7818227 33285070

[72] C. L. Fisher, G. K. Pei, Modification of a PCR-based site-directed mutagenesis method. Biotechniques 23, 570–574 (1997). 10.2144/97234bm01 9343663

[73] J. Vandesompele, K. De Preter, F. Pattyn, B. Poppe, N. Van Roy, A. De Paepe, F. Speleman, Accurate normalization of real-time quantitative RT-PCR data by geometric averaging of multiple internal control genes. Genome Biol. 3, RESEARCH0034 (2002). 10.1186/gb-2002-3-7-research0034 PMC126239 12184808

[74] S. Bolte, F. P. Cordelières, A guided tour into subcellular colocalization analysis in light microscopy. J. Microsc. 224, 213–232 (2006). 10.1111/j.1365-2818.2006.01706.x 17210054

[75] M. De Palma, L. Naldini, Transduction of a gene expression cassette using advanced generation lentiviral vectors. Methods Enzymol. 346, 514–529 (2002). 10.1016/s0076-6879(02)46074-0 11883088

[76] J. Lee, X. Cheng, J. M. Swails, M. S. Yeom, P. K. Eastman, J. A. Lemkul, S. Wei, J. Buckner, J. C. Jeong, Y. Qi, S. Jo, V. S. Pande, D. A. Case, C. L. Brooks III, A. D. MacKerell Jr., J. B. Klauda, W. Im, Charmm-gui input generator for namd, gromacs, amber, openmm, and charmm/openmm simulations using the charmm36 additive force field. J. Chem. Theory Comput. 12, 405–413 (2016). 10.1021/acs.jctc.5b00935 PMC4712441 26631602

[77] J. Lee, D. S. Patel, J. Ståhle, S. J. Park, N. R. Kern, S. Kim, J. Lee, X. Cheng, M. A. Valvano, O. Holst, Y. A. Knirel, Y. Qi, S. Jo, J. B. Klauda, G. Widmalm, W. Im, Charmm-gui membrane builder for complex biological membrane simulations with glycolipids and lipoglycans. J. Chem. Theory Comput. 15, 775–786 (2019). 10.1021/acs.jctc.8b01066 30525595

[78] W. L. Jorgensen, J. Chandrasekhar, J. D. Madura, R. W. Impey, M. L. Klein, Comparison of simple potential functions for simulating liquid water. J. Chem. Phys. 79, 926–935 (1983).

[79] J. C. Phillips, D. J. Hardy, J. D. C. Maia, J. E. Stone, J. V. Ribeiro, R. C. Bernardi, R. Buch, G. Fiorin, J. Hénin, W. Jiang, R. McGreevy, M. C. R. Melo, B. K. Radak, R. D. Skeel, A. Singharoy, Y. Wang, B. Roux, A. Aksimentiev, Z. Luthey-Schulten, L. V. Kalé, K. Schulten, C. Chipot, E. Tajkhorshid, Scalable molecular dynamics on CPU and GPU architectures with NAMD. J. Chem. Phys. 153, 044130 (2020). 10.1063/5.0014475 PMC7395834 32752662

[80] J. A. Maier, C. Martinez, K. Kasavajhala, L. Wickstrom, K. E. Hauser, C. Simmerling, ff14SB: Improving the accuracy of protein side chain and backbone parameters from ff99SB. J. Chem. Theory Comput. 11, 3696–3713 (2015). 10.1021/acs.jctc.5b00255 PMC4821407 26574453

[81] I. Ivani, P. D. Dans, A. Noy, A. Pérez, I. Faustino, A. Hospital, J. Walther, P. Andrio, R. Goñi, A. Balaceanu, G. Portella, F. Battistini, J. L. Gelpí, C. González, M. Vendruscolo, C. A. Laughton, S. A. Harris, D. A. Case, M. Orozco, Parmbsc1: A refined force field for DNA simulations. Nat. Methods 13, 55–58 (2016). 10.1038/nmeth.3658 PMC4700514 26569599

[82] S. W. de Leeuw, J. W. Perram, E. R. Smith, Simulation of electrostatic systems in periodic boundary conditions. I. Lattice sums and dielectric constants. Proc. R. Soc. A 373, 27–56 (1980).

[83] T. Darden, D. York, L. Pedersen, Particle mesh Ewald: An *N·*log(*N*) method for Ewald sums in large systems. J. Chem. Phys. 98, 10089–10092 (1993).

[84] J. P. Ryckaert, G. Ciccotti, H. J. C. Berendsen, Numerical integration of the Cartesian equations of motion of a system with constraints: Molecular dynamics of n-alkanes. J. Comput. Phys. 23, 327–341 (1977).

[85] S. E. Feller, Y. Zhang, R. W. Pasto, B. R. Brooks, Constant pressure molecular dynamics simulation: The Langevin piston method. J. Chem. Phys. 103, 4613–4621 (1995).

[86] B. Leimkuhler, E. Noorizadeh, F. Theil, A gentle stochastic thermostat for molecular dynamics. J. Stat. Phys. 135, 261–277 (2009).

[87] T. Tubiana, J.-C. Carvaillo, Y. Boulard, S. Bressanelli, TTClust: A versatile molecular simulation trajectory clustering program with graphical summaries. J. Chem. Inf. Model. 58, 2178–2182 (2018). 10.1021/acs.jcim.8b00512 30351057

[88] F. Murtagh, P. Legendre, Ward’s hierarchical agglomerative clustering method: Which algorithms implement ward’s criterion? J. Classif. 31, 274–295 (2014).

[89] Schrödinger LLC, The PyMOL Molecular Graphics System, version 2.0 (Schrödinger LLC, New York, NY, 2002).

[90] W. Zheng, Q. Wuyun, P. L. Freddolino, Y. Zhang, Integrating deep learning, threading alignments, and a multi-MSA strategy for high-quality protein monomer and complex structure prediction in CASP15. Proteins 91, 1684–1703 (2023). 10.1002/prot.26585 PMC10840719 37650367

[91] J. Yang, Y. Zhang, Protein structure and function prediction using I-TASSER. Curr. Protoc. Bioinformatics 52, 5.8.1–5.8.15 (2015). 10.1002/0471250953.bi0508s52 PMC4871818 26678386

[92] J. Jumper, R. Evans, A. Pritzel, T. Green, M. Figurnov, O. Ronneberger, K. Tunyasuvunakool, R. Bates, A. Žídek, A. Potapenko, A. Bridgland, C. Meyer, S. A. A. Kohl, A. J. Ballard, A. Cowie, B. Romera-Paredes, S. Nikolov, R. Jain, J. Adler, T. Back, S. Petersen, D. Reiman, E. Clancy, M. Zielinski, M. Steinegger, M. Pacholska, T. Berghammer, S. Bodenstein, D. Silver, O. Vinyals, A. W. Senior, K. Kavukcuoglu, P. Kohli, D. Hassabis, Highly accurate protein structure prediction with AlphaFold. Nature 596, 583–589 (2021). 10.1038/s41586-021-03819-2 PMC8371605 34265844

[93] H. Park, G. R. Lee, D. E. Kim, I. Anishchenko, Q. Cong, D. Baker, High-accuracy refinement using Rosetta in CASP13. Proteins 87, 1276–1282 (2019). 10.1002/prot.25784 PMC6851472 31325340

[94] Y. Yan, H. Tao, J. He, S. Y. Huang, The HDOCK server for integrated protein–protein docking. Nat. Protoc. 15, 1829–1852 (2020). 10.1038/s41596-020-0312-x 32269383

[95] H. M. Berman, J. Westbrook, Z. Feng, G. Gilliland, T. N. Bhat, H. Weissig, I. N. Shindyalov, P. E. Bourne, The Protein Data Bank. Nucleic Acids Res. 28, 235–242 (2000). 10.1093/nar/28.1.235 PMC102472 10592235

[96] G. M. Boratyn, A. A. Schäffer, R. Agarwala, S. F. Altschul, D. J. Lipman, T. L. Madden, Domain enhanced lookup time accelerated BLAST. Biol. Direct 7, 12 (2012). 10.1186/1745-6150-7-12 PMC3438057 22510480

[97] S. Henikoff, J. G. Henikoff, Amino acid substitution matrices from protein blocks. Proc. Natl. Acad. Sci. 89, 10915–10919 (1992). 10.1073/pnas.89.22.10915 PMC50453 1438297

[98] S. L. Dürr, A. Levy, U. Rothlisberger, Metal3D: A general deep learning framework for accurate metal ion location prediction in proteins. Nat. Commun. 14, 2713 (2023). 10.1038/s41467-023-37870-6 PMC10175565 37169763

[99] E. Neria, S. Fischer, M. Karplus, Simulation of activation free energies in molecular systems. J. Chem. Phys. 105, 1902–1921 (1996).

[100] P. Mark, L. Nilsson, Structure and dynamics of the TIP3P, SPC, and SPC/E water models at 298 K. J. Phys. Chem. 105, 9954–9960 (2001).

[101] S. Miyamoto, P. A. Kollman, SETTLE: An analytical version of the SHAKE and RATTLE algorithm for rigid water models. J. Comput. Chem. 13, 952–962 (1992).

[102] G. Fiorin, M. L. Klein, J. Hénin, Using collective variables to drive molecular dynamics simulations. Mol. Phys. 111, 3345–3362 (2013).

