# Supplementary material for: Phosphorylation by CaMKIV regulates the transcriptional repressor activity and half-life of REST/NRSF
Source: Sci Adv. 2026 Oct 16;12(42):eaec0781. doi: 10.1126/sciadv.aec0781 (PMC13674487; doi:10.1126/sciadv.aec0781)
Supplement: Supplementary file 1 [file sciadv.aec0781_sm.pdf]

Science Advances
AAAS

Supplementary Materials for

Phosphorylation by CaMKIV regulates the transcriptional repressor activity and half-life of REST/NRSF

Hanako Tsushima Semini *et al.*

Corresponding author: Luca Maragliano, l.maragliano@staff.univpm.it; Fabio Benfenati, fabio.benfenati@iit.it

Sci. Adv. **12**, eaec0781 (2026)
DOI: 10.1126/sciadv.aec0781

This PDF file includes:

Supplementary Methods
Tables S1 and S2
Figs. S1 to S20
References

SUPPLEMENTARY METHODS

Molecular Modeling of Wild-Type REST

The amino acid sequence V130-H267: "VAEDKCRSSKAKPFRCKPCQYEAESEEQFVHHIRIHSAK KFFVEESAEKQAKAWESGSSPAEEGEFSKGPIRCDRCGYNTNRYDHYMAHLKHHLRAGENERIY KCIICTYTTVSEYHWRKHLRNHFPRKVYTCSKCNYFSDRKNNYVQHVRTHTGERPYKCELCPYSS SQKTHLTRHMRTHSGEKPFKCDQCNYVASNQHEVTRHARQVHNGPKPLNCPHCDYKTADRSNF KKHVELHVNPRQFNCPVCDYAASKKCNLQYHFKSKH", accounting for the first eight zinc finger domains (ZNF) of the wt mouse REST protein (mREST) was used as input for three independent structure-prediction program: D-I-TASSER (90,91), AlphaFold2 (92), and RoseTTAFold (93). Based on visual inspection with PyMOL (89), the D-I-TASSER (90) model was selected as the most reliable structural candidate for subsequent docking experiments (**Figure S9**).

Protein–DNA Docking

To assemble the REST–RE1 complex, the selected V130–H267 REST fragment and the RE1 DNA sequence GCTTTCAGCACCACGGACAGC (containing three upstream nucleotides) were submitted to HDOCK (94), a hybrid docking server that combines template-based modeling with free docking. HDOCK (94) uses a soft rigid-body docking strategy with limited backbone flexibility and relies on a knowledge-based scoring function integrating interface energy, buried surface area, and statistical potentials. The top-ranked binding mode (model 1, **Figure S8 - Step 4**) served as the basis for subsequent simulations.

Multiple Sequence Alignment

The REST amino acid sequence comprising residues V130–H267 was used as the query for a homology search against the Protein Data Bank (PDB) (95). The NCBI Domain Enhanced Lookup Time Accelerated BLAST (DELTA-BLAST) algorithm (96) was employed, with the maximum number of target sequences set to 50. An expectation value (E-value) threshold of 1e-5 and a word size of 3 were specified. Alignments were scored using the BLOSUM62 substitution matrix (97), with gap penalties of 11 for gap existence and 1 for gap extension. Alignments were generated using a conditional compositional score matrix to correct for sequence-specific amino acid biases, and low-complexity regions were masked to minimize spurious matches driven by compositional heterogeneity.

Template Selection for REST-RE1 Complex

PDB entries retrieved from the multiple sequence alignment (MSA) were downloaded and inspected via PyMOL (89). Duplicate chains and crystallization cofactors were removed, and records displaying missing residues were excluded both to prevent artifacts in the superimposition and to ensure structural completeness. Pairwise structural alignments were then performed between REST and each remaining protein chain, using REST as the mobile selection in the alignment procedure (**Figure S8 – Step 3; Figure S10A**). The corresponding RMSD values are reported in **Table S2**.
Among the aligned structures, the protein–DNA complex deposited under PDB ID 1ubd was selected as the optimal template for REST–RE1 complex assembly (**Figure S10B**). This structure contains four zinc finger domains—only one fewer than REST—providing a biologically meaningful reference for modeling protein–DNA interactions. In addition, 1ubd contains no missing residues and yielded one of the lowest RMSD values in the dataset. Although a few entries produced marginally lower RMSDs, they failed to meet the full set of selection criteria outlined above. 1ubd was also instrumental in resolving ambiguities among the alternative docking poses by offering a validated

spatial framework for the orientation of zinc finger domains relative to DNA. Furthermore, the conserved coordination geometry observed in 1ubd served as a structural guide for the accurate placement and chelation of Zn^{2+} ions within the modeled REST Zn-finger domains, ensuring correct folding and stability of the DNA-binding interface.

Metal3D Benchmarks

PDB entries 1a1h and 4r2a were selected as benchmark structures based on their low pairwise RMSD and their representation of distinct model organisms. Specifically, 1a1h corresponds to a murine zinc finger protein and 4r2a to a human zinc finger protein. Prior to analysis, all divalent zinc ions and nucleic acid molecules were stripped from the coordinate files, yielding protein-only structures suitable for being submitted to Metal3D convolutional neural network (98).

The coordinates of Zn^{2+} ions were then predicted at an isovalue probability threshold of $p = 0.9$ and subsequently integrated into the corresponding protein-only structures. These reconstituted assemblies were superimposed onto the original PDB entries to quantify deviations between the experimental and predicted Zn^{2+} ion positions (**Figure S11**).

Zn^{2+} Ion Coordinate Prediction and Model Incorporation

The predicted REST protein structure was submitted to the Metal3D (98) Colab Notebook, allowing the remaining convolutional neural network layers to initialize and process the input. A grid with a resolution of 0.5 Å was generated to compile per-residue metal-binding probability scores. Zn^{2+} 3D coordinates corresponding to an isovalue probability threshold of $p = 0.9$ were then extracted and incorporated into the protein-only REST model. Multiple coordinate sets were predicted for each zinc-binding domain at this threshold. To select a single set of Zn^{2+} coordinates for each domain, the predictions were superimposed onto the zinc-binding site of the template structure (PDB: 1ubd) with PyMOL (89) to assess positional deviations and identify the most structurally consistent coordinates (**Figure S12**).

Geometrical Optimization of ZNF Domains via Molecular Dynamics (MD) Simulations

The solution builder plug-in of the CHARMM-GUI web server (76,77) was used to prepare the system and generate input files for MD simulations. The DNA–protein assembly was embedded in a rectangular box of TIP3P (99,100) water molecules, with a minimum solute-to-box-edge distance of 15 Å applied along all directions. The total system charge was neutralized by adding Na^+ and Cl^- ions until a physiological concentration of 0.15 M was reached. The whole system amounts to ∽112000 atoms. Simulations were performed with NAMD 2.14 (79) combining the AmberFf14SB (80) force filed for the protein and the BSC1 (81) force field for the nucleic acid. Tetragonal periodic boundary conditions (PBC) were applied. The Particle Mesh Ewald method was used to treat electrostatic interactions and to avoid box surface effects with a maximum grid spacing of 1 Å and sixth-order beta splines. A cut-off of 9 Å was employed for Lennard-Jones interactions, which were smoothly switched off starting at 13 Å. Chemical bond distances involving hydrogen atoms were constrained using the SHAKE (101) algorithm. A time-step of 2 fs was utilized. Prior to initiating the production phase, the system underwent a rigorous protocol consisting of energy minimization followed by a 250 ns-long equilibration, conducted in an isobaric–isothermal (NPT) ensemble (P = 1 atm, T = 310 K). Harmonic positional restraints were applied to the Cα atoms of the protein backbone to preserve the overall fold and the Colvar (collective variables) module (102) was employed to maintain the proper zinc ion binding. Specifically, the Colvar Coordination Number variable was defined using a set of donor atoms surrounding the Zn^{2+} center to drive the system toward its native pseudo-tetrahedral coordination geometry (**Figure S13**). Once the proper Zn^{2+} coordination environment was achieved, the simulation progressed to the production stage. At this point, all positional restraints on Cα atoms were released. The Coordination Number restraint on the zinc coordination shell was

maintained for all production runs. Throughout the simulation, the target pressure (P=1) and temperature (T=310 K) were regulated using a Nosé–Hoover Langevin piston (85) and Langevin thermostat (86), ensuring stable thermodynamic conditions under NPT ensemble constraints.

SUPPLEMENTARY TABLES

Table S1. **Phospho-potential scores of putative mouse and human REST sites.**

Prediction software	REST S177/S182		REST T294/T299		REST S322/S327		Notes
	mouse	human	mouse	human	mouse	human	
Musite	0.260	0.260	0.414	0.414	0.782	0.779	Generic PK
NetPhos3.1	0.433	0.425	0.457	0.457	0.462	0.462	CaMKs
KinasePhos3	NA	--	NA	--	NA	0.790	CMGC PKs
Kinexus	NA	No	NA	Yes	NA	Yes	Generic PK
Scansite	1.183	1.253	--	--	2.067	2.067	CLK2

The scores of the three putative CaMKIV phosphorylation sites obtained by scanning the mouse and human REST *consensus* sequences with distinct phosphor-site prediction softwares predicting the phosphorylation potential are shown. PK, protein kinase; CMGC, CMGC family kinases (Cyclin-dependent, Mitogen-activated, Glycogen synthase, and CDK-like kinases); CLK2, dual specificity protein kinase; --, not detected; ND, not available.

Table S2. **RMSD values resulting from structural alignments.**

RCSBPDB ID	***RMSD (Å)***
1a1f	1.49
1a1h	1.24
1a1i	1.19
1a1l	1.31
1jk1	1.18
1mey	1.47
1p47	1.23
1ubd	1.80
1zaa	1.29
2i13	1.93
2jp9	1.30
4r2a	1.23
5egb	2.75
5t0u	1.95
5v3g	2.69
5v3j	4.03
5v3m	4.05
5wjq	3.96
5yef	2.10
5yeg	8.41
6b0o	2.03
6blw	1.83
6e93	2.19
6ml2	1.36
6wmi	2.51
7eyi	3.04
7n5s	2.92
7n5w	10.52
7txc	6.72
7ysf	2.05
8e3e	3.25
8ssq	2.43
8sss	1.97
8sst	1.94
8ssu	2.61

RMSD values are in Å. Superimpositions were performed taking the protein chain of REST (V130-H267) as the mobile selection in PyMOL. RMSD values are given in Å.

SUPPLEMENTARY FIGURES

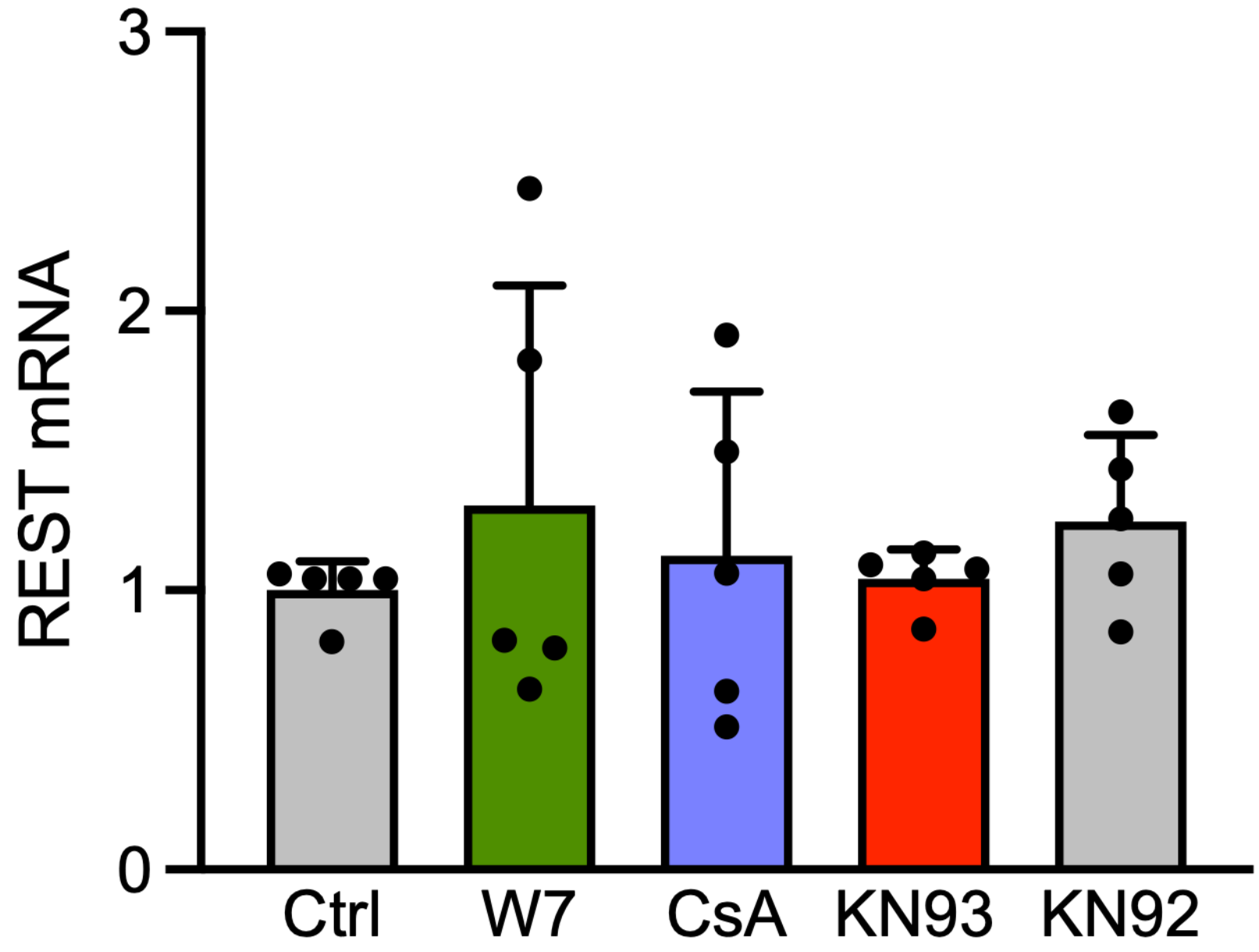

Figure S1. Inhibition of Ca^{2+}-calmodulin-dependent activities does not affect REST transcription in primary cortical neurons. REST mRNA levels in primary cortical neurons (DIV14) treated with vehicle, the Ca^{2+}-calmodulin antagonist W7 (20 µM), the phosphatase inhibitor cyclosporin A (CsA; 1 µM), the cell-permeable CaMK inhibitor KN93 (10 µM), and its inactive derivative KN92 (10 µM). Mean (± sem with individual experimental points) REST mRNA levels assessed by real-time qPCR. $p>0.83$, one-way ANOVA/Tukey's tests (n=5 independent experiments).

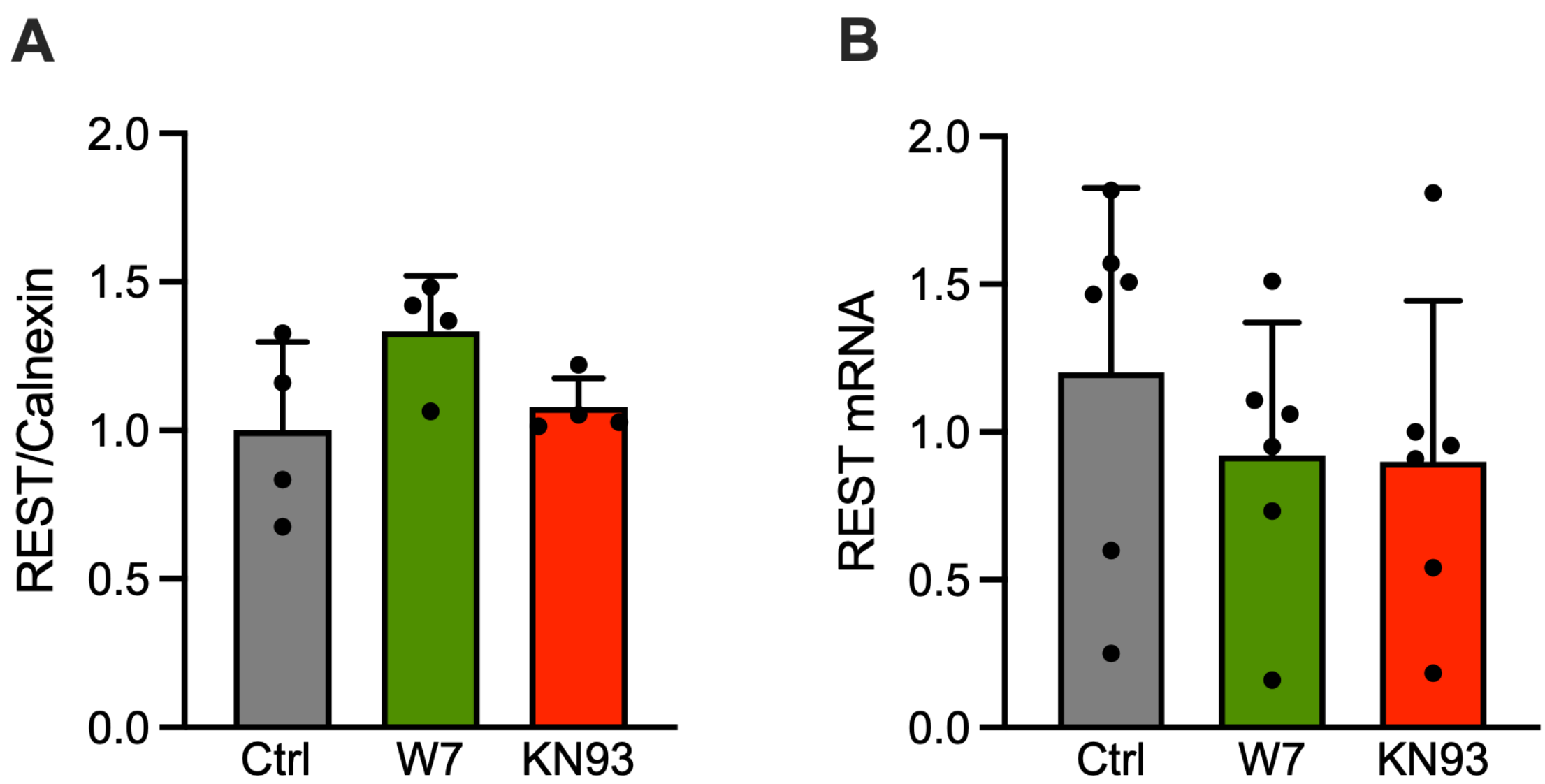

Figure S2. Inhibition of Ca^{2+}-calmodulin-dependent activities does not affect REST transcription and translation in primary astrocytes. A. Immunoblotting of REST in primary astrocytes treated with vehicle, the Ca^{2+}-calmodulin antagonist W7 (20 µM), and the CaMK inhibitor KN93 (10 µM). Calnexin was used as a control for equal loading. REST expression is shown as the mean (± sem) ratio of REST to calnexin immunoreactivities, with individual experimental points. $p>0.09$; one-way ANOVA/Dunnett's tests *vs* control (n=4 independent experiments). **B.** REST mRNA levels in primary astrocytes treated as in A. Mean (± sem) REST mRNA levels assessed by real-time qPCR, with individual experimental points. $p>0.54$, one-way ANOVA/Dunnett's tests *vs* control (n=6 independent experiments).

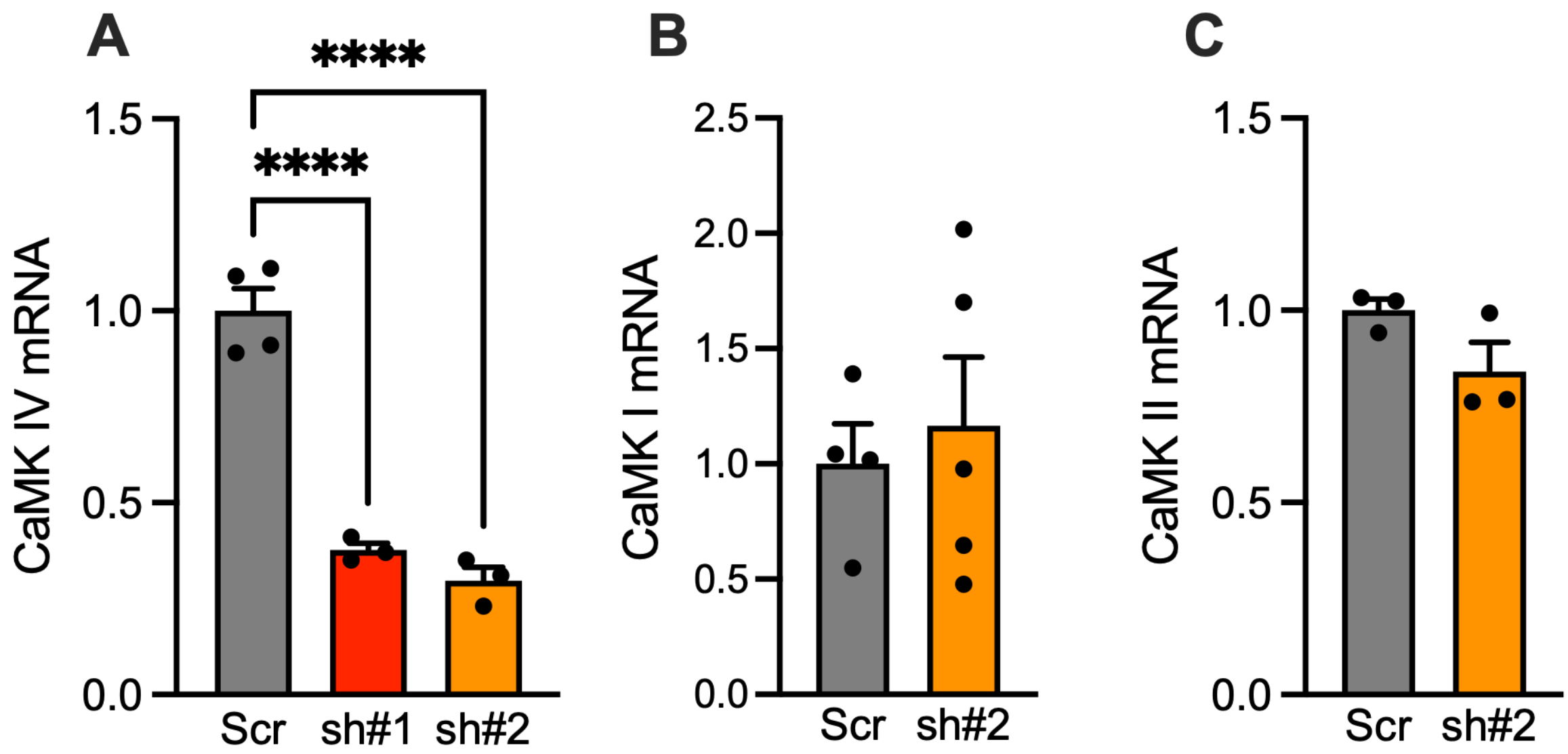

Figure S3. Silencing the CaMKIV by RNA interference does not affect REST, CaMKI, and CaMKII expression in primary neurons. Two short hairpin RNAs (sh#1 and sh#2) were used to test the efficacy of CaMKIV downregulation. The mean (± sem; with individual experimental points) CaMKIV mRNA levels (**A**), assessed by real-time qPCR, were significantly downregulated by both shRNAs. CaMKIV downregulation by sh#2 did not affect the mRNA levels of CaMKI (**B**) and CaMKII (**C**). A: ****$p<0.0001$, one-way ANOVA/Tukey's tests (n=4 independent experiments). B,C: $p=0.65$ (B), $p=0.16$ (C); unpaired Student's *t*-test (n= 5 and 3 independent experiments for B and C, respectively).

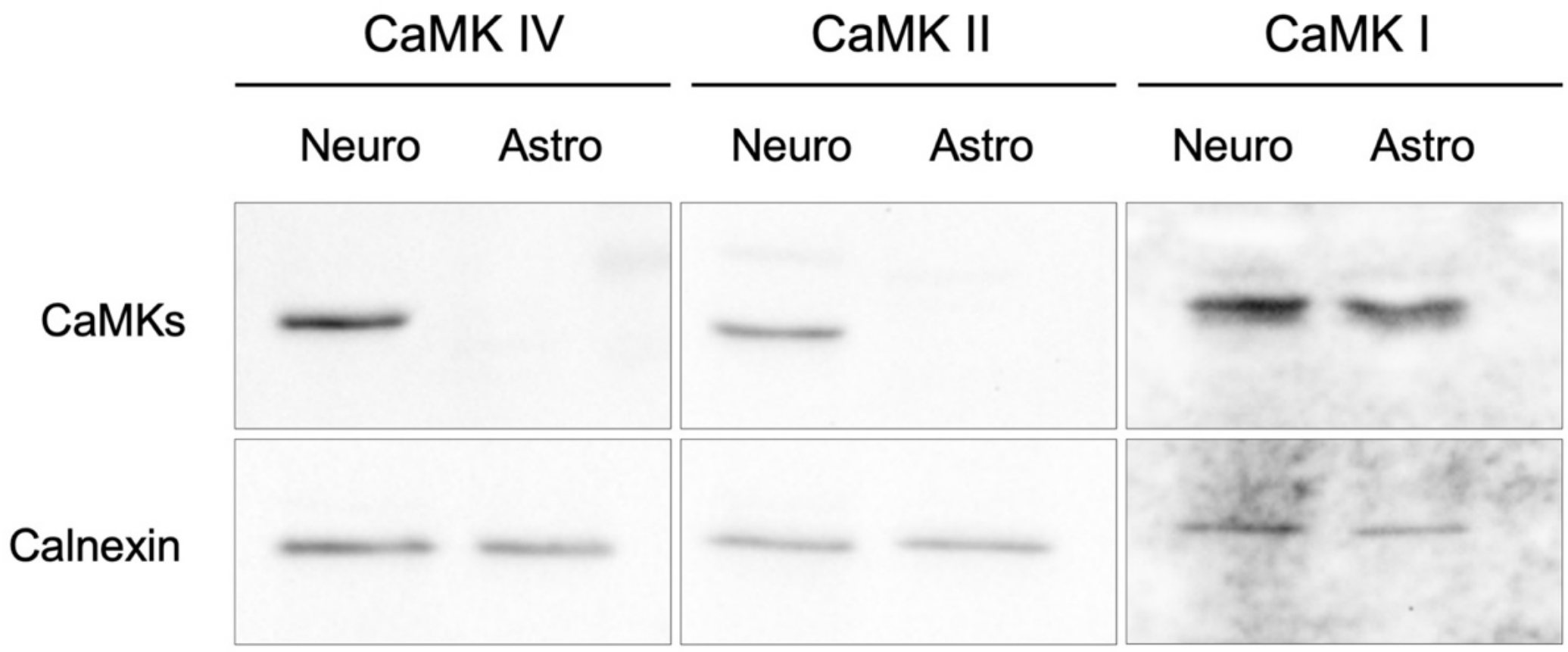

Figure S4. The expression of CaMKII and CaMKIV is neuron specific. Immunoblots of CaMKI, CaMKII, and CaMKIV in primary neurons and primary astrocytes. Calnexin was used as a loading control. While CaMKI is expressed to a similar extent in both neurons and astrocytes, CaMKII and CaMKIV display a purely neuron-specific expression.

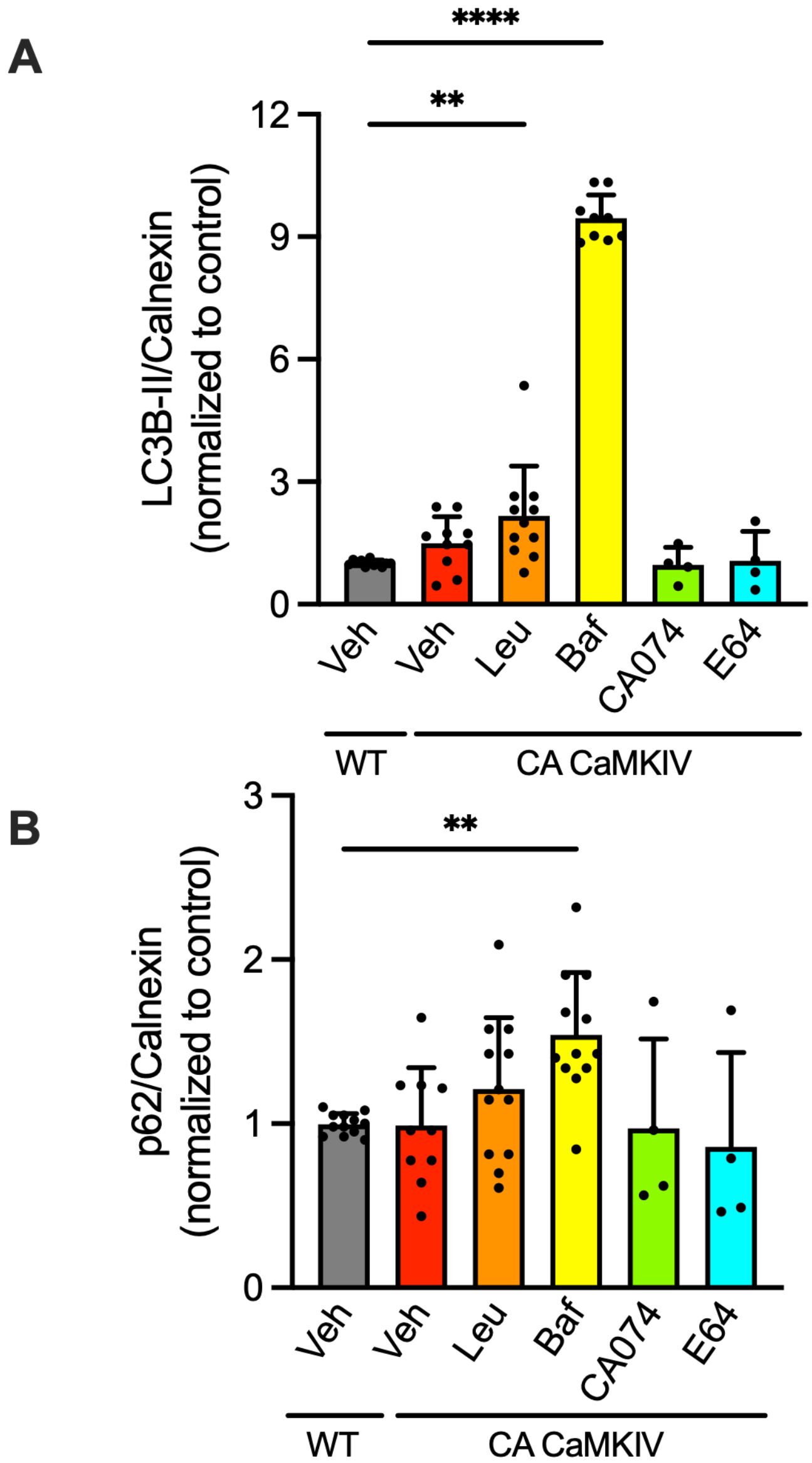

Figure S5. Bafilomycin induces autophagy blockade in HEK293T cells. HEK293T cells transfected with either WT or constitutively active (CA) CaMKIV, and treated with cycloheximide, were treated for 16 h with vehicle (DMSO, Veh), leupeptin (Leu, 40 µM), bafilomycin (Baf, 100 nM), CA074 (10 µM) or E64 (10 µM), as described in Figure 3C, which reports the representative immunoblots, monitoring the expression of REST, calnexin, and the autophagy markers p62 and LC3B-II. Quantitative analysis of the autophagic block was performed by calculating the mean (± sem; with individual experimental points) protein levels of the autophagy markers LC3B-II (**A**) and p62 (**B**), shown as ratios relative to calnexin immunoreactivity under the various experimental conditions and normalized to the control (WT CaMKIV/Veh). A: **p=0.0022 CA/Leu *vs* control, ****p<0.0001 CA/Baf *vs* control (n=3-6 independent preparations). B: **p=0.0039 CA/Baf *vs* control (n=4-8 independent preparations). One-way ANOVA/Dunnett's tests.

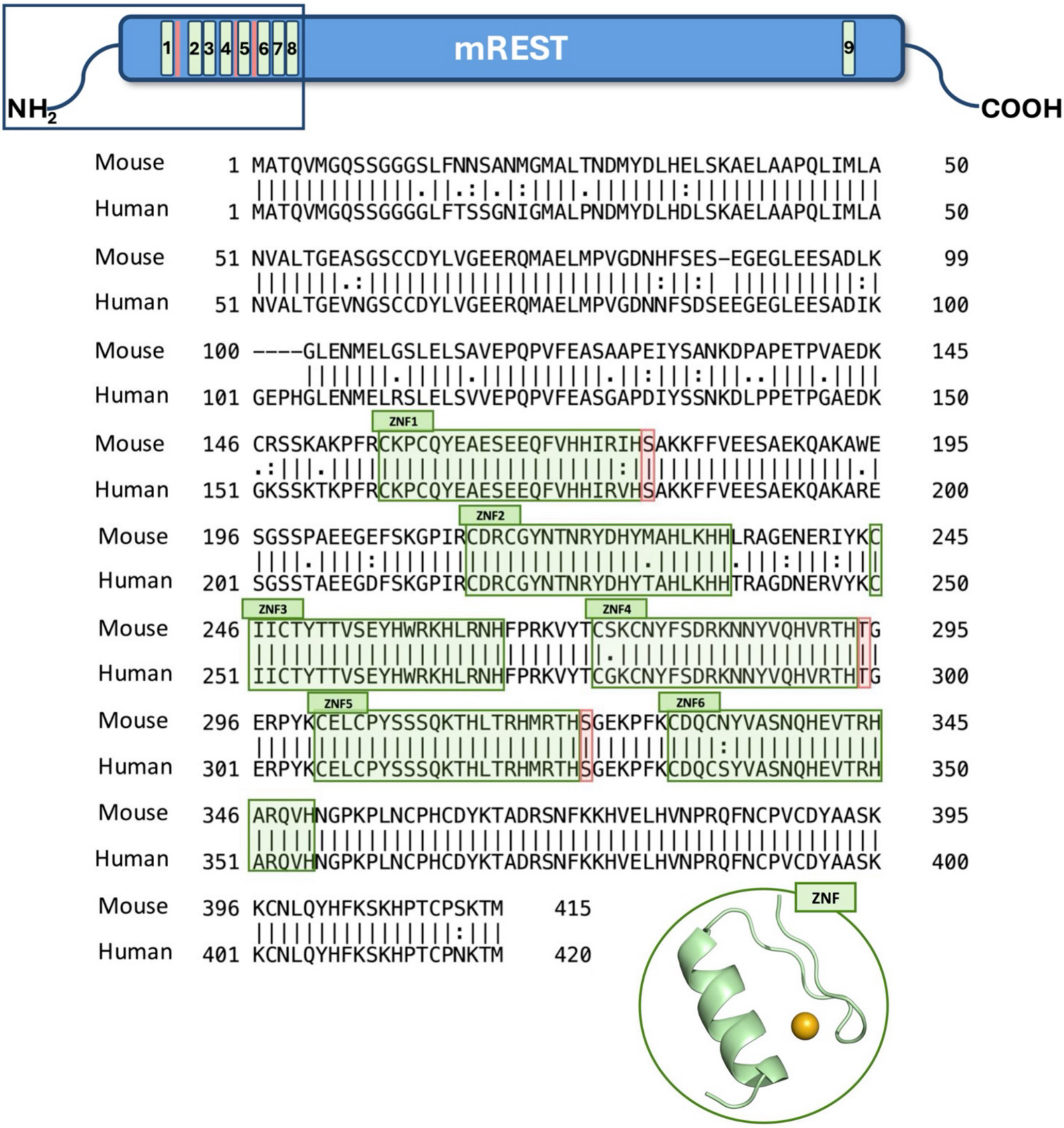

Figure S6. Alignment of the primary structures of human and mouse REST. Amino acids are shown in single-letter code. Sequences shaded in green refer to the Zn-finger domains (numbered 1 to 6), while three putative phosphorylation sites ($S^{177/182}$, $T^{294/299}$, and $S^{322/327}$ in mouse and human, respectively) that match the *consensus* sequence for CaMKs (S/T-XX-R/K) and are conserved between mouse and human REST are highlighted in red.

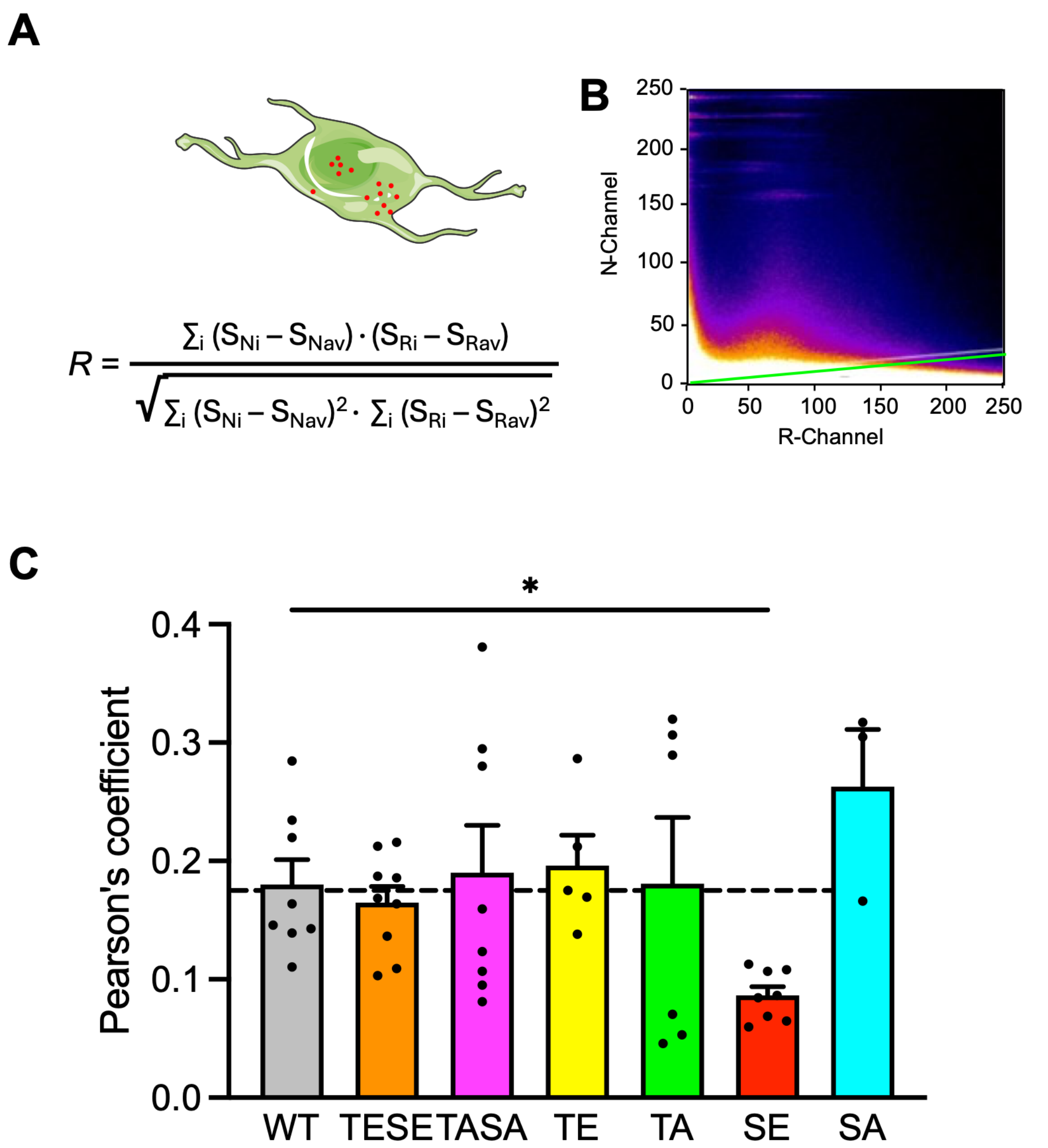

Figure S7. Nuclear/cytosolic shuttling of REST mutants in HEK293T cells as evaluated by spatial correlation analysis. A. Scheme of the image analysis of a stained HEK293T cell (Hoechst in dark green and REST in red) with the formula used to calculate Pearson's coefficient R (S_{Ni}, nuclear Hoechst signal, sample "i"; S_{Nav}, average nuclear Hoechst signal: S_{Ri}, REST signal, sample "i"; S_{Rav}, average REST signal). **B.** Sample scatter plot output of the Colocalization Finder Plugin. X and Y axes are the intensity of the pixels on the scatter plot for the Hoechst (H) and anti-REST (R) fluorescence channels, respectively. The green line displays Pearson's correlation. Heatmap colors represent pixel density. **C.** Mean (± sem) of the Pearson's coefficient evaluating the spatial correlation between the pixel distribution of the Hoechst nuclear fluorescence and that of REST immunoreactivity. *p=0.0125, SE *vs* WT; one-way Welch's ANOVA/Dunnett's tests.

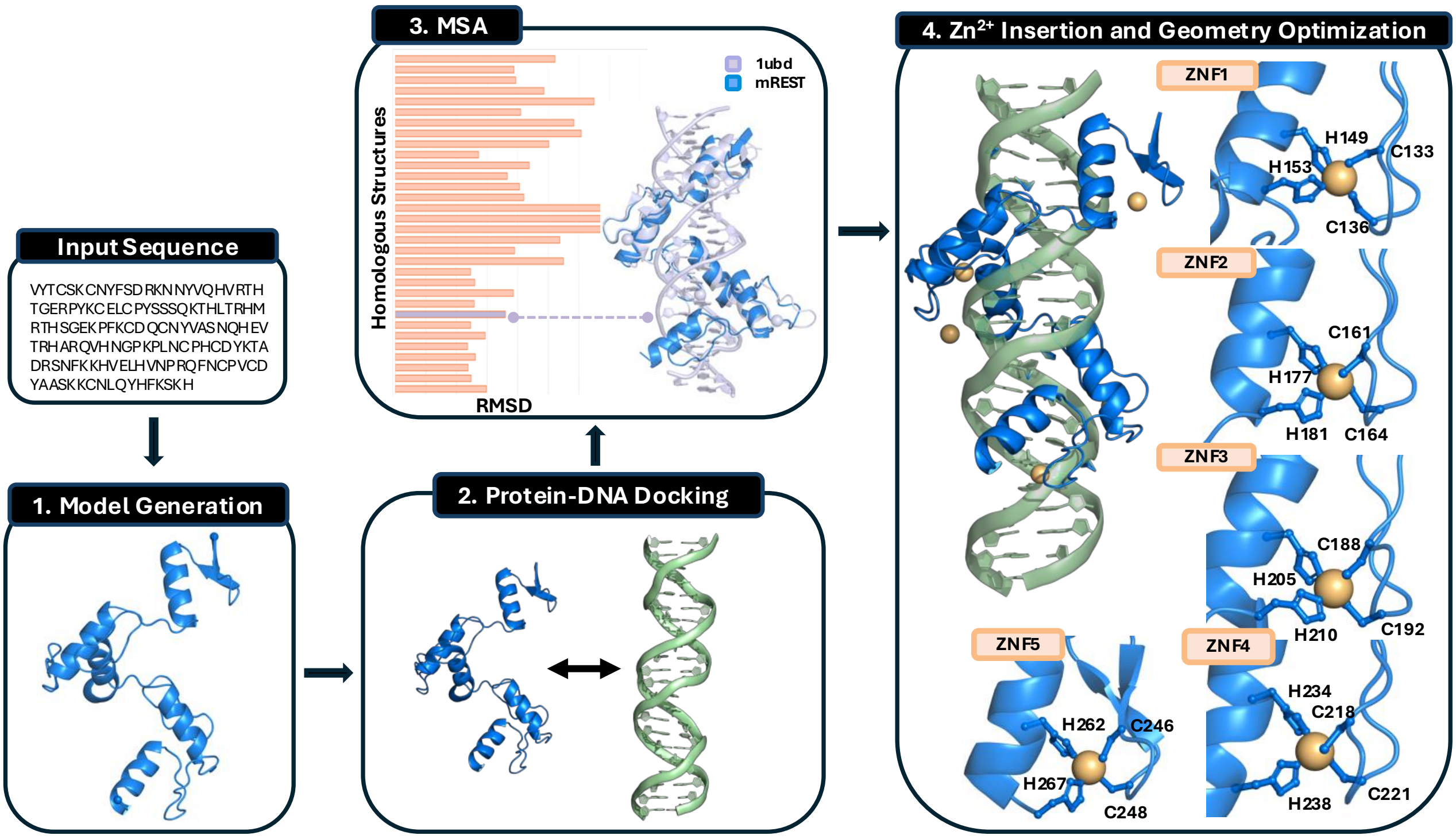

Figure S8. Schematic overview of the REST:RE1 structural modeling workflow. Schematic representation of the tailored, multi-step structural modeling pipeline developed to assemble the REST:RE1 protein–DNA complex. The workflow is organized into four interdependent modules, each enclosed in black-framed boxes and connected by arrows to indicate the sequential order of the steps. The pipeline begins with the generation of structural models for the individual REST and RE1 components (1), followed by a protein–DNA docking stage (2) to produce initial complex conformations. This is followed by multiple sequence alignment (3) aimed at identifying suitable structural templates to guide REST:RE1 interface refinement, helping to resolve ambiguities in docking poses, Zn^{2+} ions positioning, and to improve the biological relevance and accuracy of the modeled interactions. In the final step (4), Zn^{2+} ions are inserted into the Zn-finger domains of REST, and local coordination geometries are optimized to ensure correct chelation. Methodological details are provided in the Supplementary Information.

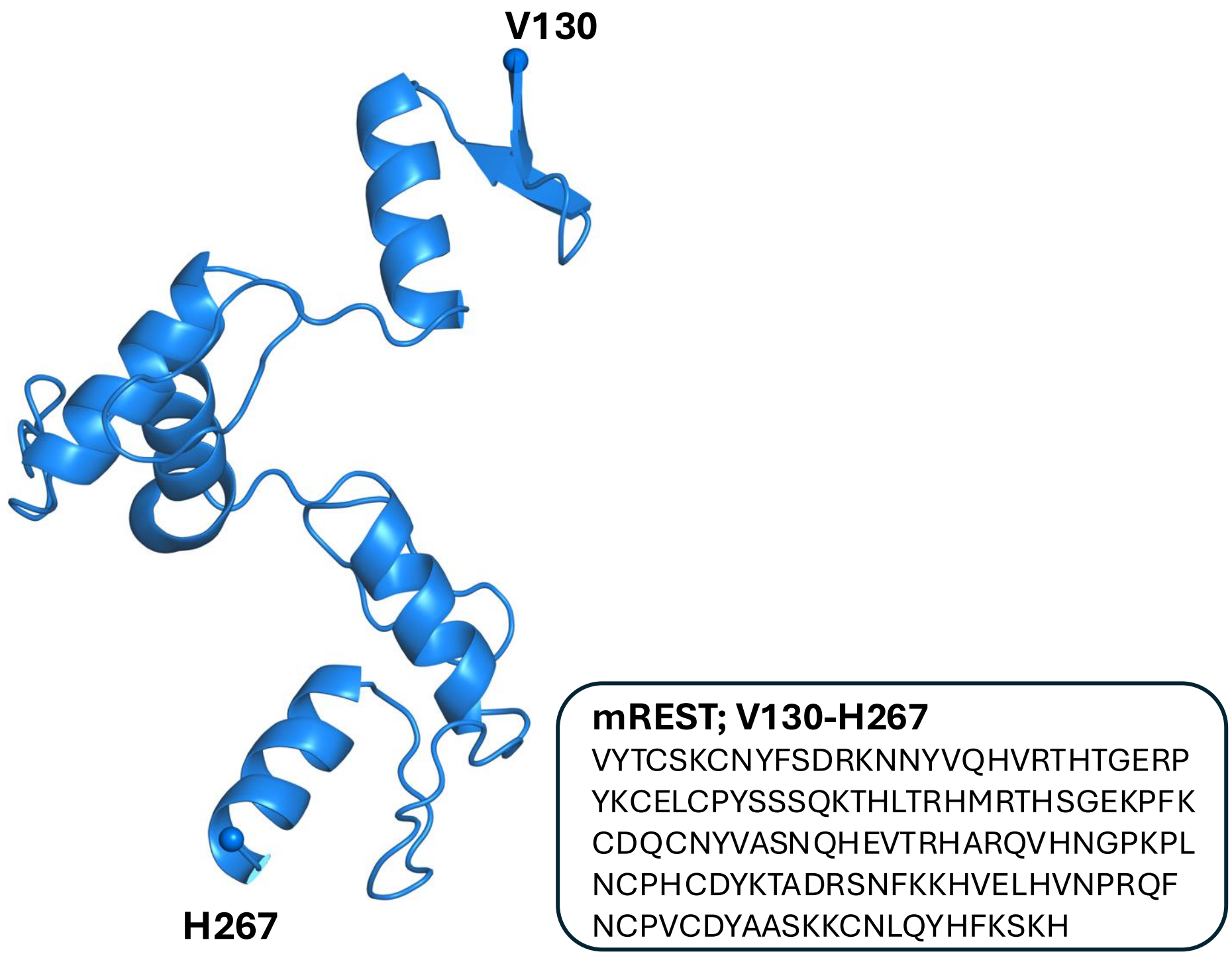

Figure S9. **Molecular modeling of mREST ZNF domains.** D-I-TASSER prediction for mREST amino acid string (V130-H267), displayed as blue cartoon; α-carbons of N- and C-terminal residues are represented as spheres. In the black rectangle, the primary sequence employed as input to the D-I-TASSER structural modeling algorithm is shown.

A

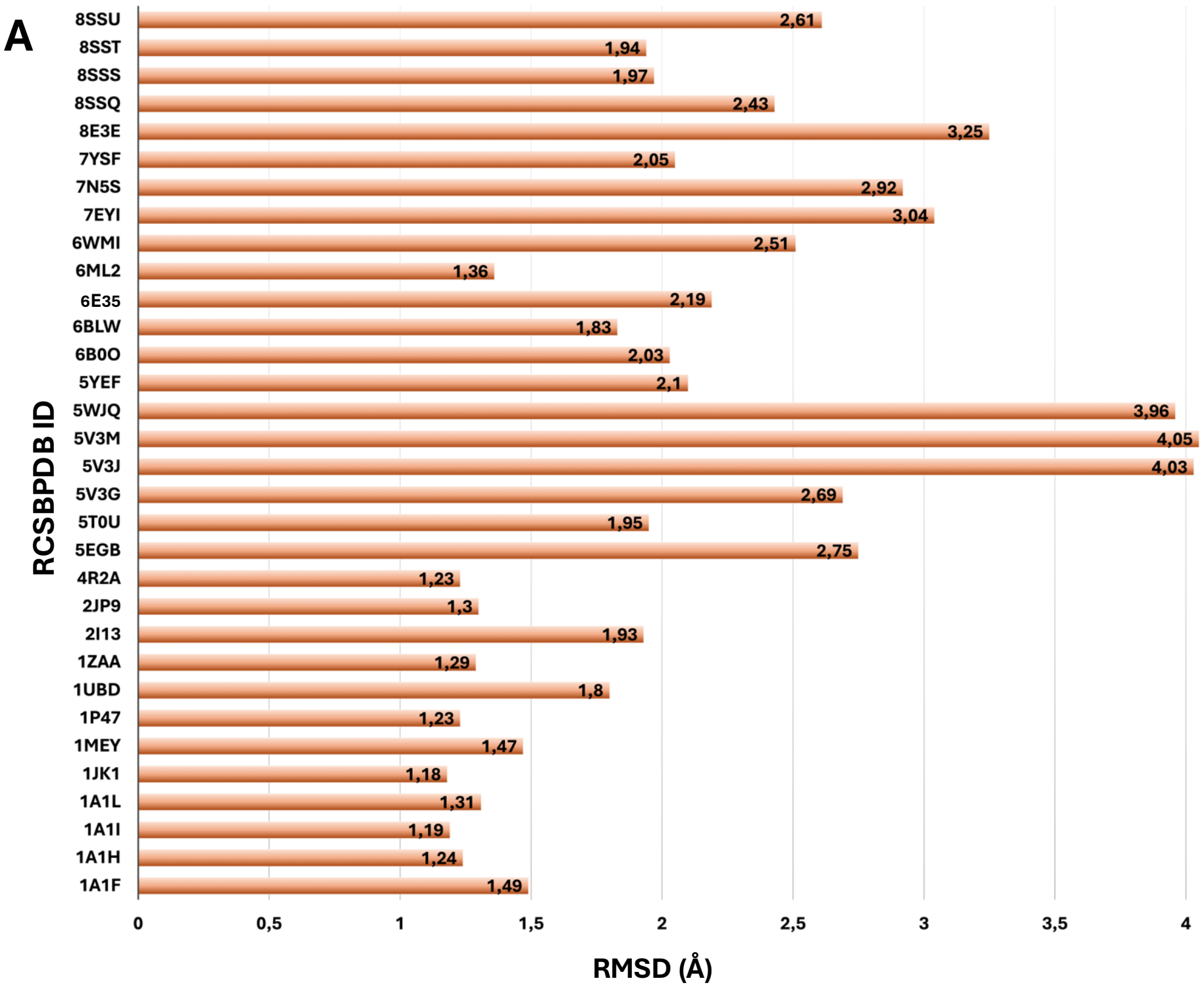

B

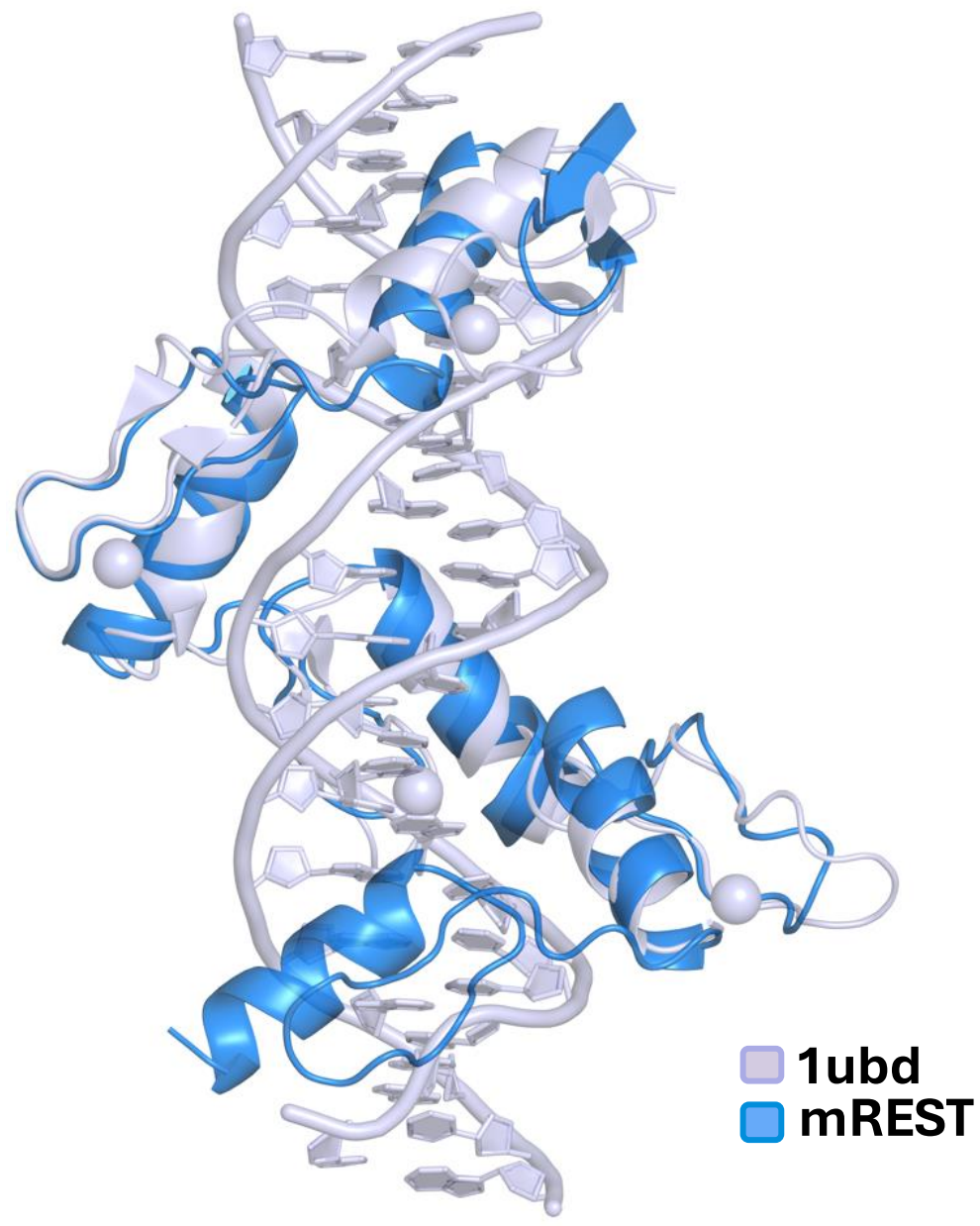

Figure S10. Structural template identification for REST:RE1. A. Root-mean-square deviations (RMSD, in Å) resulting from pairwise structural alignments between the modeled mREST ZF region and experimentally determined Zn-finger domains from the RCSB Protein Data Bank. Individual RMSD values for each alignment are annotated at the end of each bar. **B.** Superimposed structures of mREST (blue) and PDBID 1ubd, in lilac. Both protein and DNA components are displayed in cartoon representation. Coordinated Zn^{2+} ions are shown as spheres.

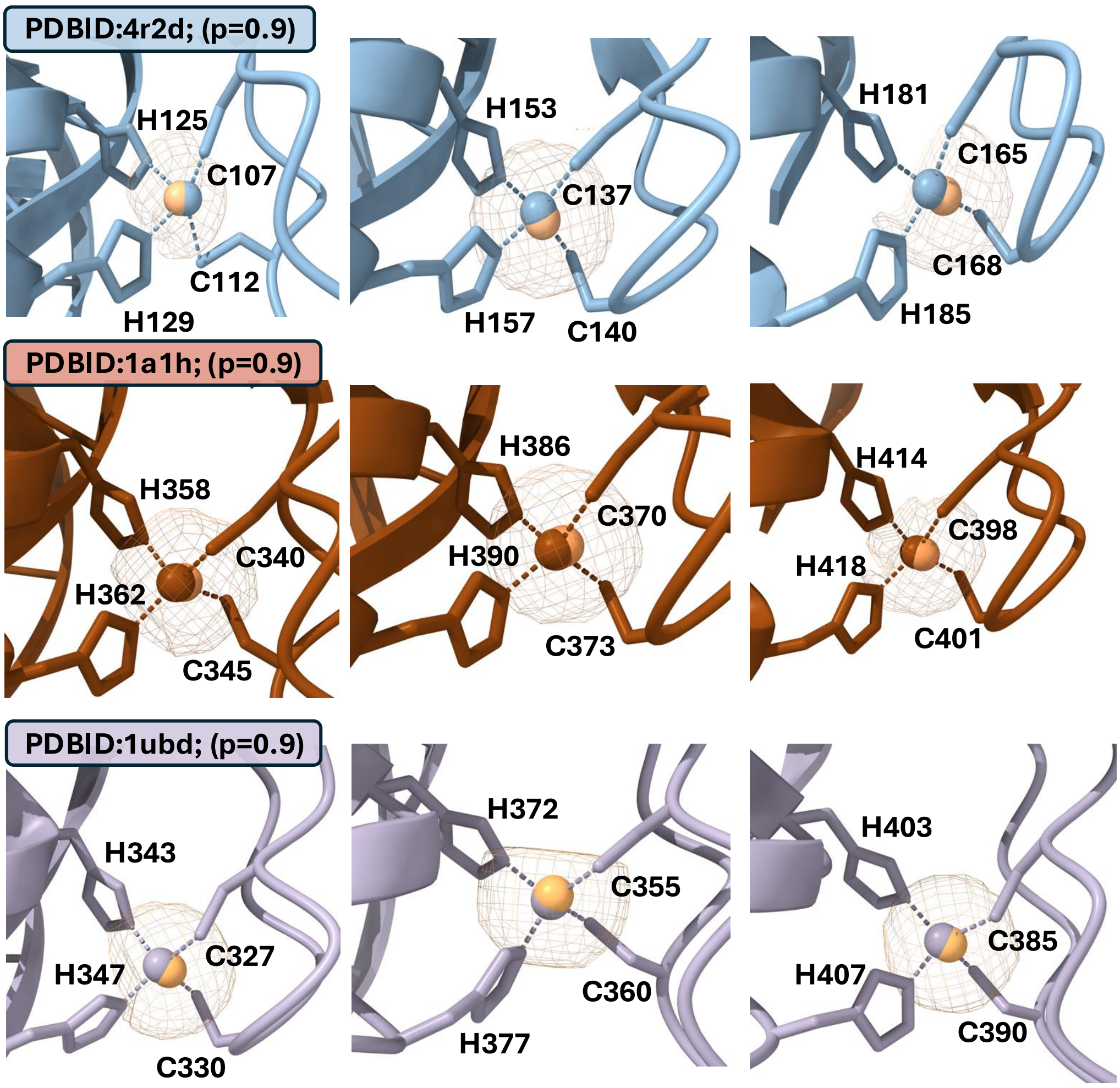

Figure S11. Benchmarking of Metal3D predictions for Zn^{2+} coordination sites. Comparison between predicted (orange) and known Zn^{2+} position for a set of experimentally determined protein structures. Protein backbones are reported in cartoon, while the side chains of zinc-chelating residues are shown as sticks. Transparent mesh surfaces indicate probability iso-surfaces of p = 0.9 for ion position.

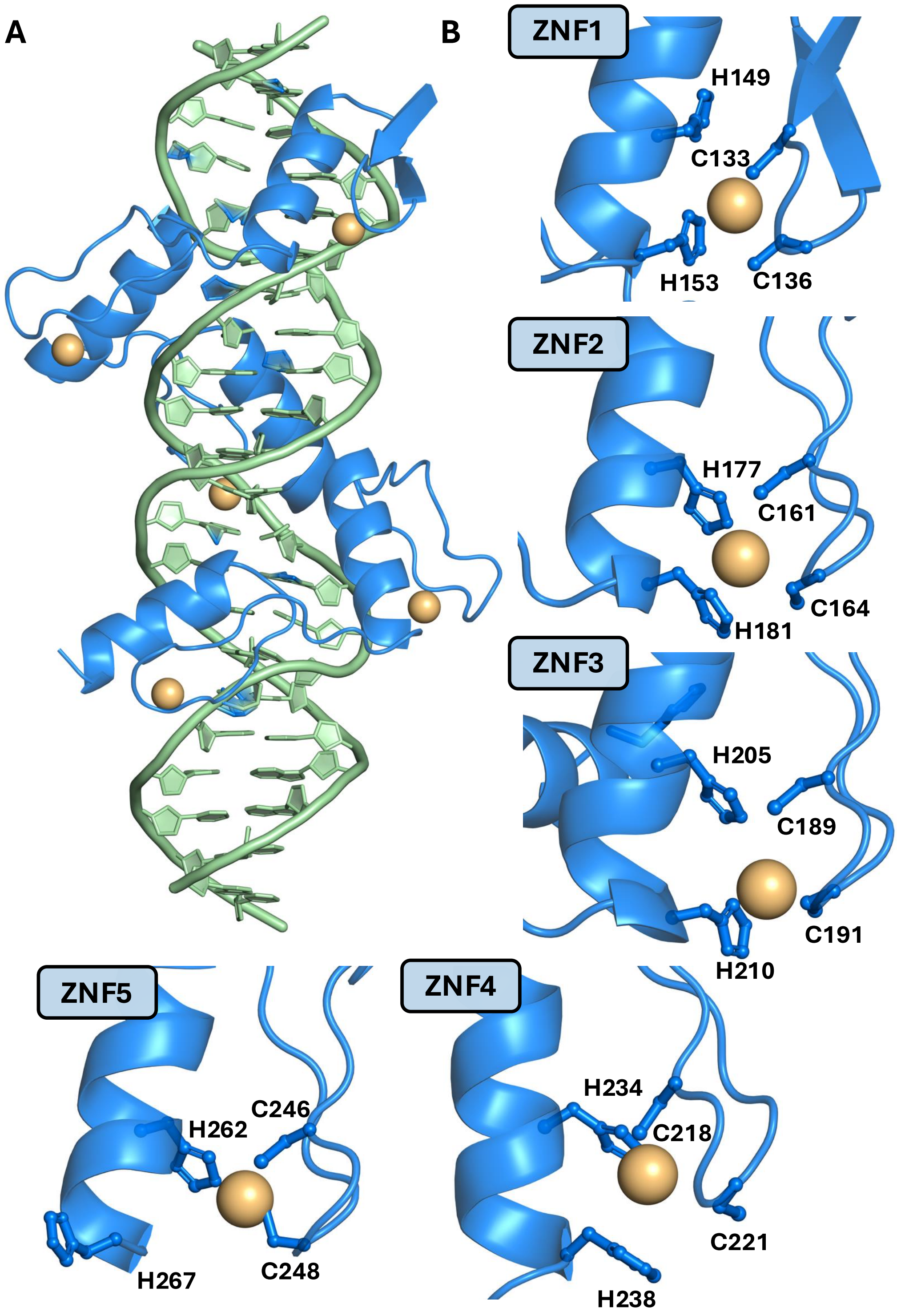

Figure S12. Pre-optimized REST ZNF domains docked onto the RE1 DNA element. A. Top-ranked docking conformation of the mREST ZNF array bound to its cognate RE1 DNA motif. **B.** Pre-optimized structures showing non-refined geometry of ZNF1 through ZNF5. Protein and DNA molecules are depicted in cartoon representation and colored blue and green, respectively; Zn^{2+}-coordinating side chains are rendered as ball-and-stick, while coordinated Zn^{2+} ions are shown as orange spheres.

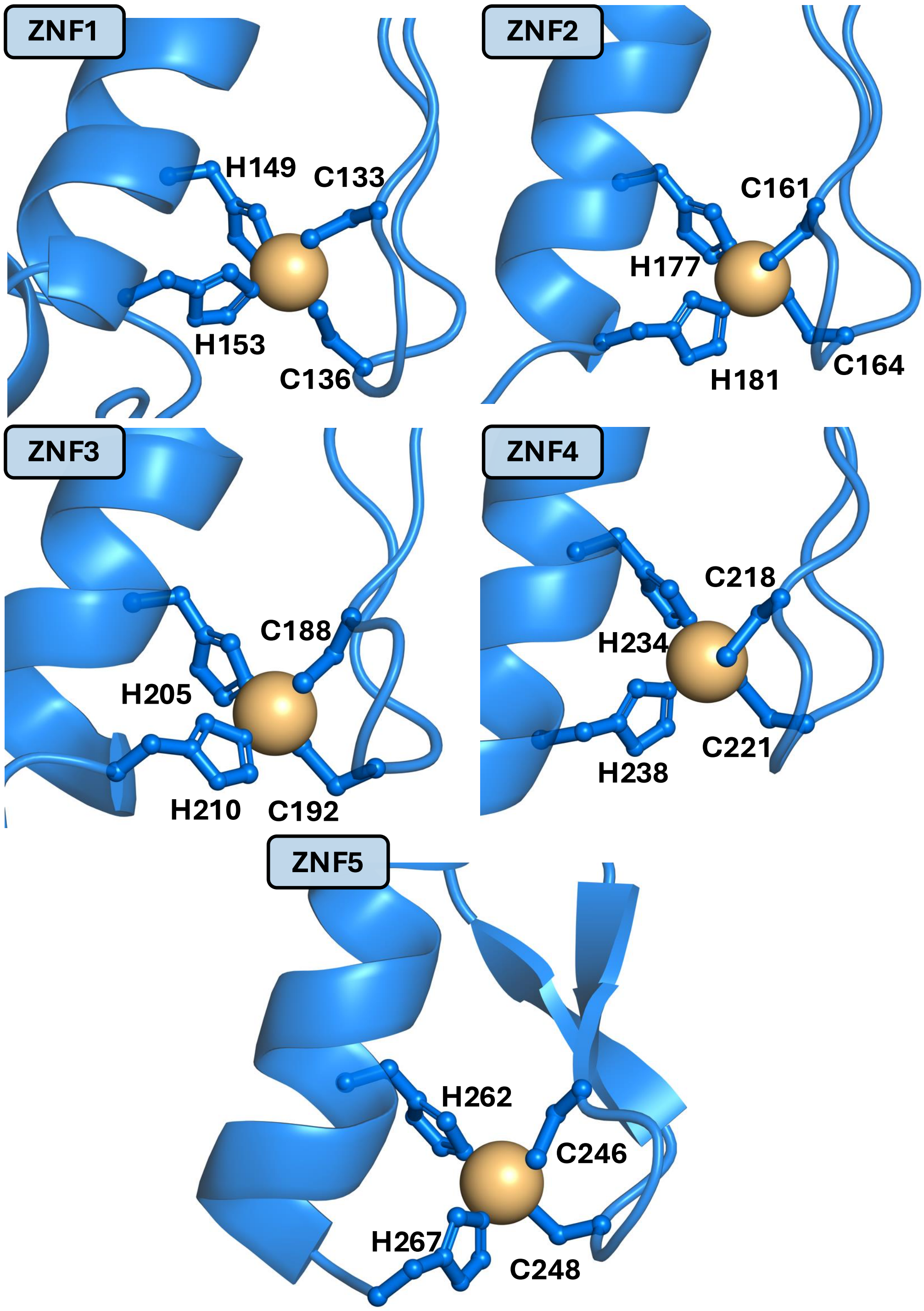

Figure S13. Optimized Zn^{2+}-coordination spheres. Representative snapshots at the end of the refinement step, showing canonical pseudo-tetrahedral coordination geometries of all Zn^{2+} ions. The protein backbone is shown as a blue cartoon, with coordinating residues represented as sticks and spheres. Zn^{2+} ions are visualized as orange spheres.

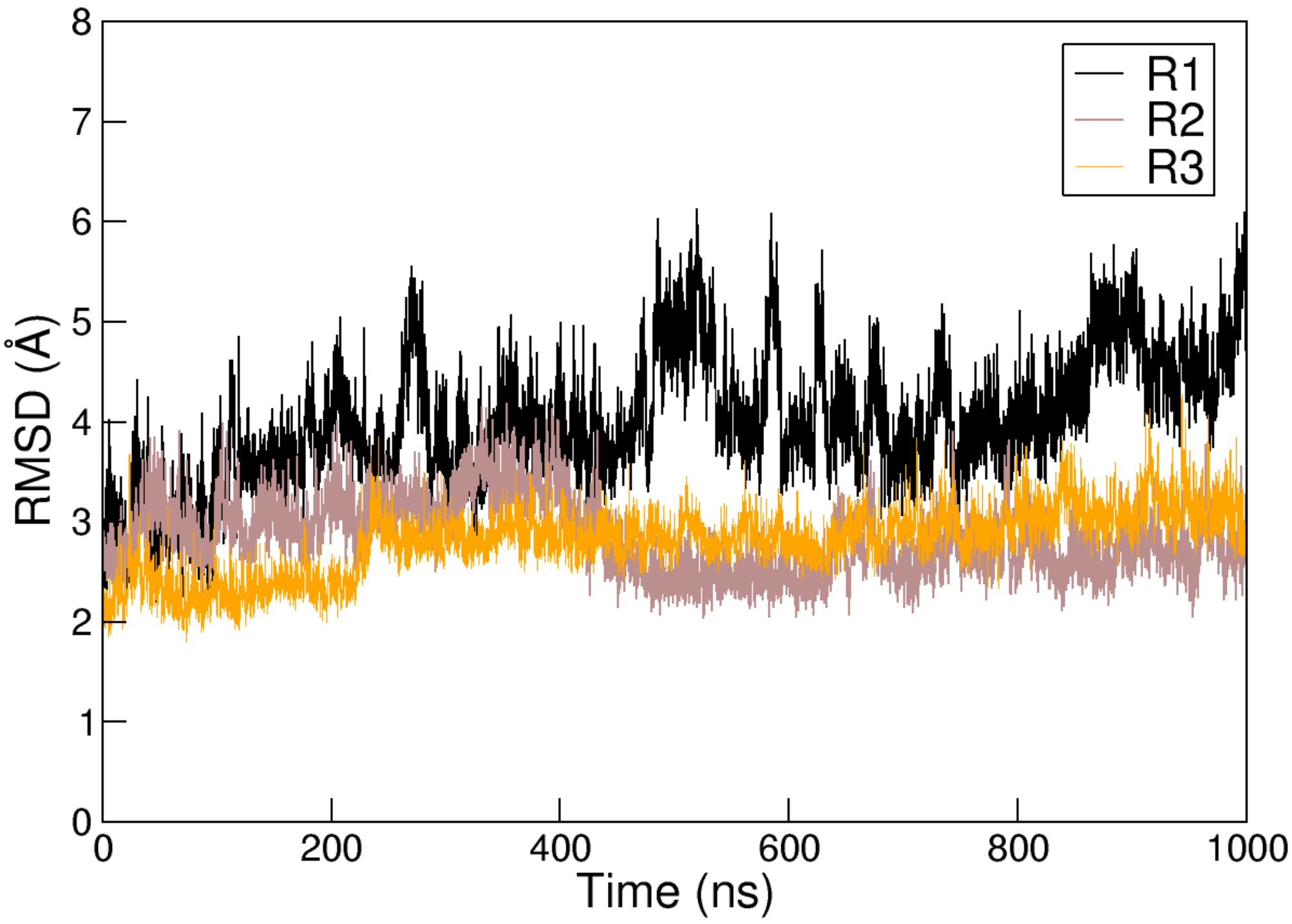

Figure S14. RMSD profiles of the WT mREST:RE1 complex. Time evolution of the root mean square deviation (RMSD) profiles of the full mREST:RE1 complex calculated over the course of three independent MD simulations. Each trajectory was generated under identical simulation conditions, starting from the same minimized and equilibrated initial structure. RMSD values were computed using all heavy atoms of both mREST and the RE1 DNA segment, after least-squares fitting to the initial frame based on the complete assembly.

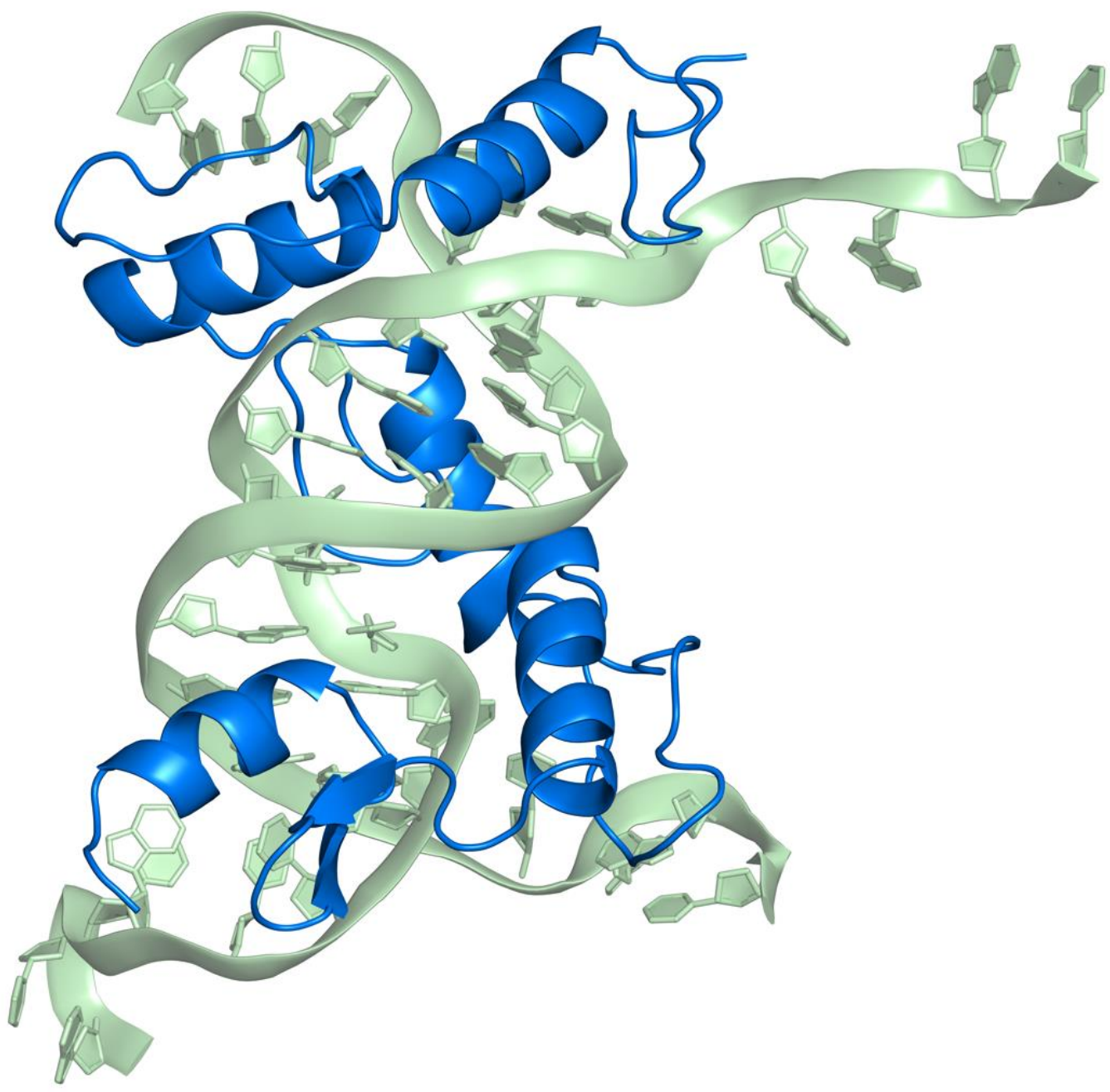

Figure S15. Fragilization of DNA termini leads to S322E system collapse. Structural snapshot illustrating the collapse of the REST:RE1 complex carrying the S322E phosphomimic substitution. The protein is shown in blue and the DNA double helix in green. The image captures a severe distortion of the canonical B-form DNA conformation, with pronounced bending and fraying at the terminal base pairs, leading to global destabilization of the protein–DNA interface. The REST domain loses its defined binding orientation, with helices disengaging from the DNA major groove and extensive disruption of side-chain contacts. These conformational aberrations underscore the critical importance of DNA end integrity in maintaining REST:RE1 structural stability, and suggest that perturbations such as the S322E mutation may exacerbate interface vulnerability by promoting electrostatic repulsion and misalignment.

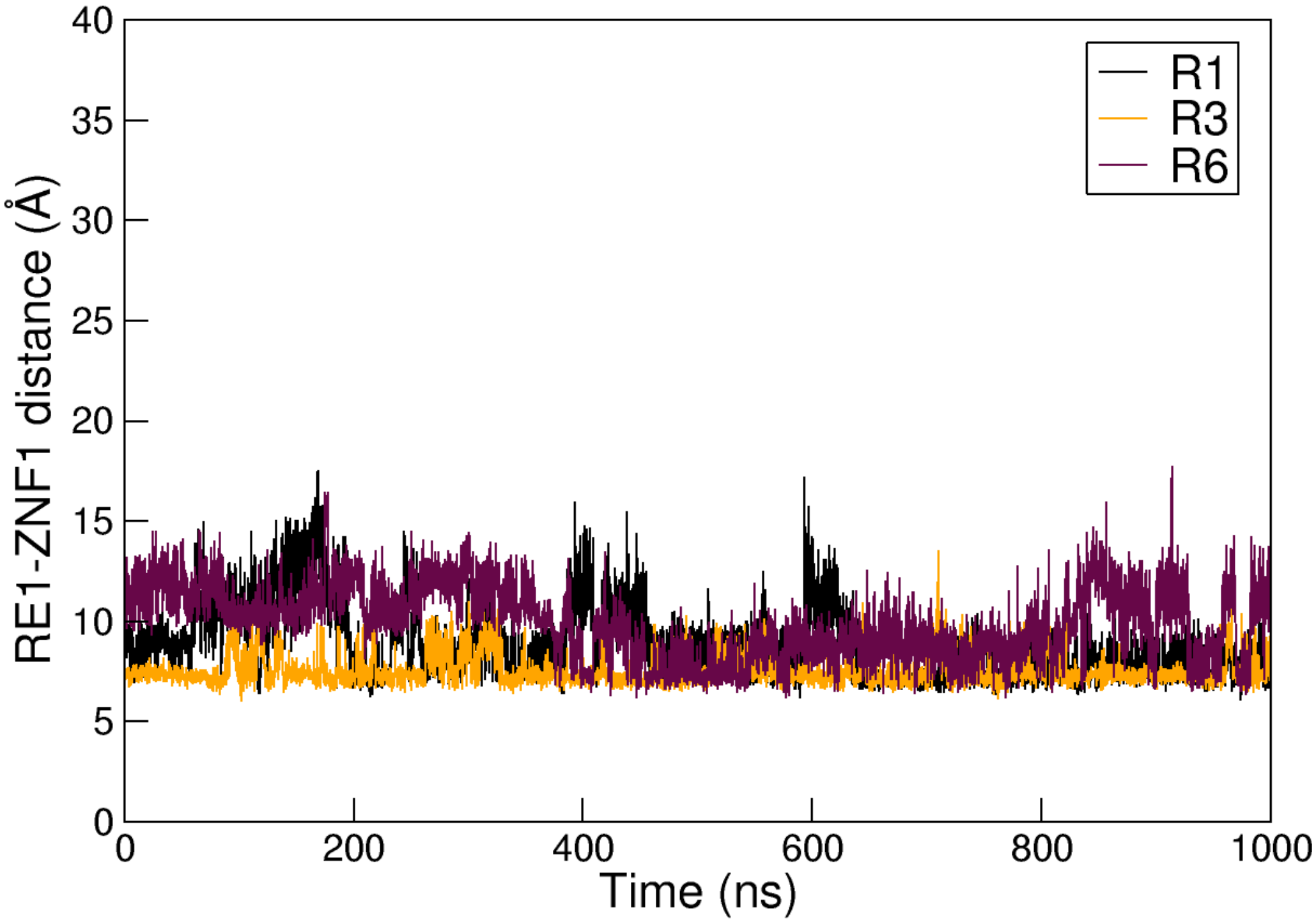

Figure S16. Time evolution of the RE1-ZNF1 distance for selected replicas of mutant S322E. Distance between RE1 and ZNF1 measured over time for three independent MD simulations, replica R1 (black), R3 (orange), and R6 (maroon), of the S322E phosphomimic variant of the mREST:RE1 complex. The distance is calculated between the phosphorus atom of the RE1 DNA segment and the center of mass of the Zn^{2+}-coordinating atoms within the first ZNF domain (ZNF1) of mREST.

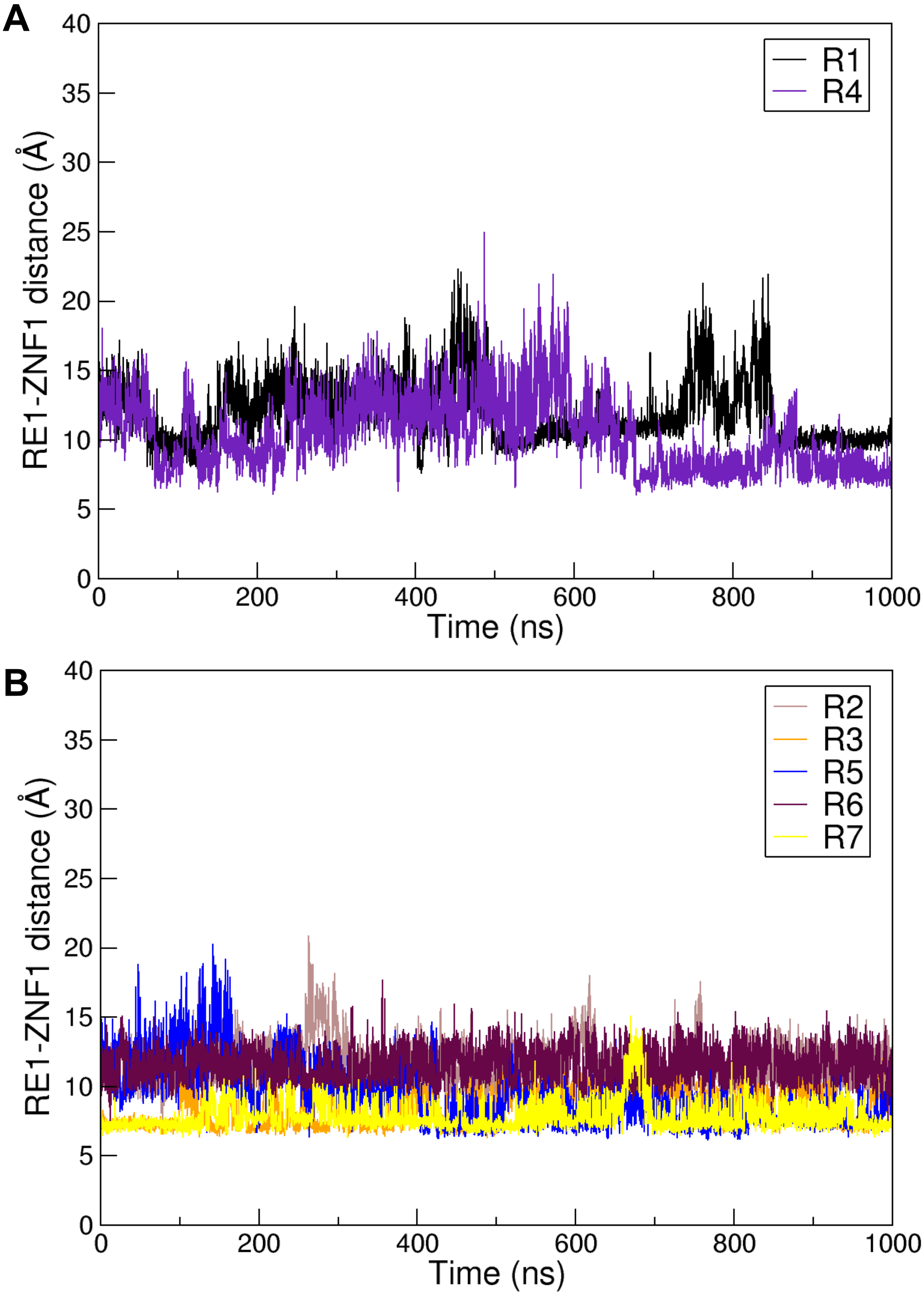

Figure S17. Time evolution of the RE1–ZNF1 distance for selected replicas of mutant T294E. A. Distance between RE1 and ZNF1measured over time for two independent MD simulations, replica R1 (black) and R4 (purple) of the T294E phosphomimic variant of the mREST:RE1 complex. **B.** Same for replica R2 (orange), R3 (brown), R5 (blue), R6 (maroon), and R7 (yellow).

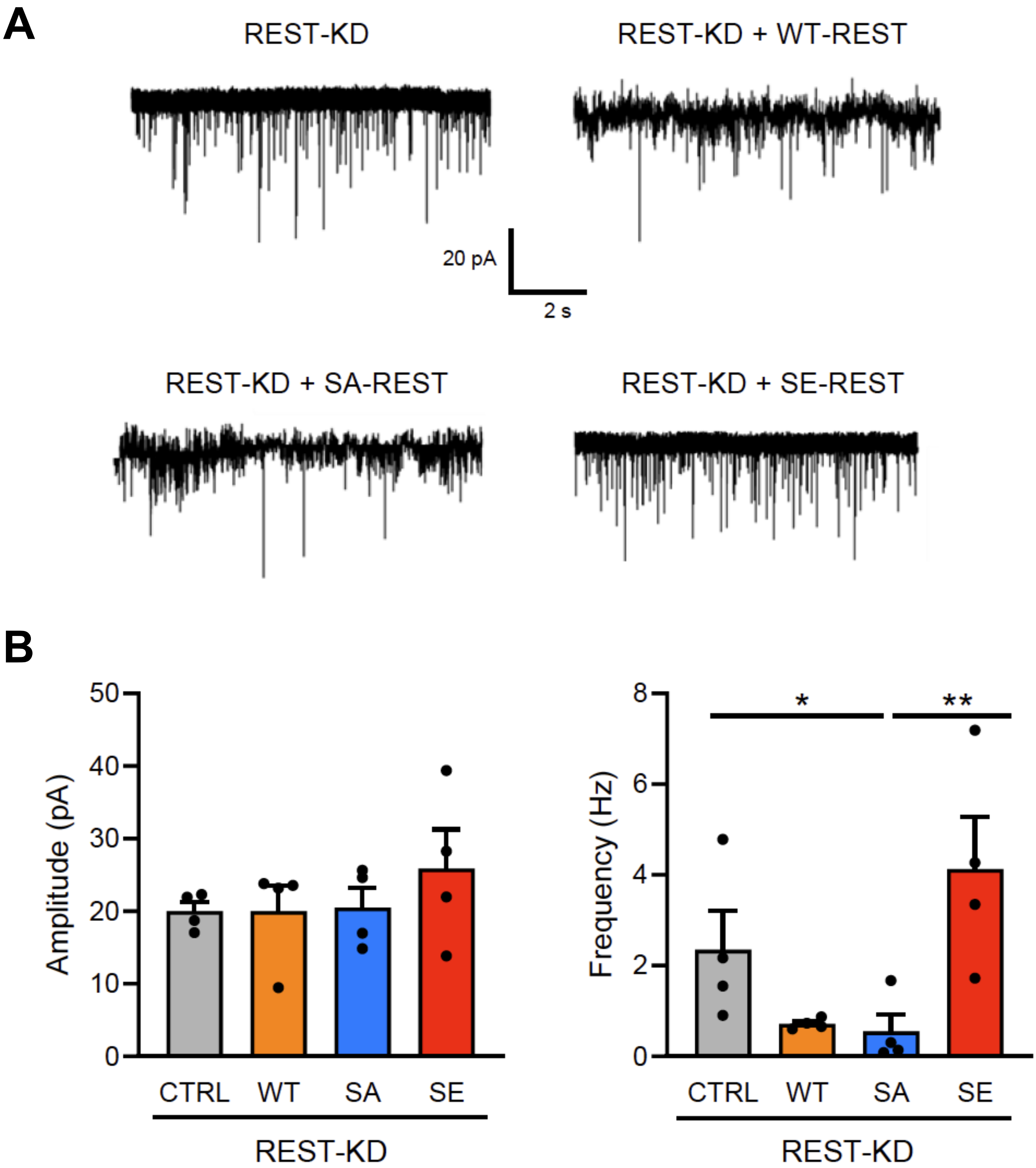

Figure S18. The phosphomimic REST mutant fails to rescue the increase in mEPSC frequency induced by REST silencing in primary neurons. A. Representative traces of mEPSCs recorded in REST-silenced primary cortical neurons (REST-KD) transduced with a control vector, WT REST, the non-phosphorylatable (SA) mutant, or the phosphomimic (SE) mutant at DIV7 and recorded at DIV14. **B***.* Mean (± sem with individual experimental points) of the mEPSC amplitude (*left*) and frequency (*right*) in the 4 experimental groups (n = 4-5 for each experimental group). *p<0.05, **p<0.01; one-way ANOVA/Holm-Šídák (frequency: *p=0.0243 Ctrl *vs* WT or SA; **p=0.0040 SE *vs* SA) tests.

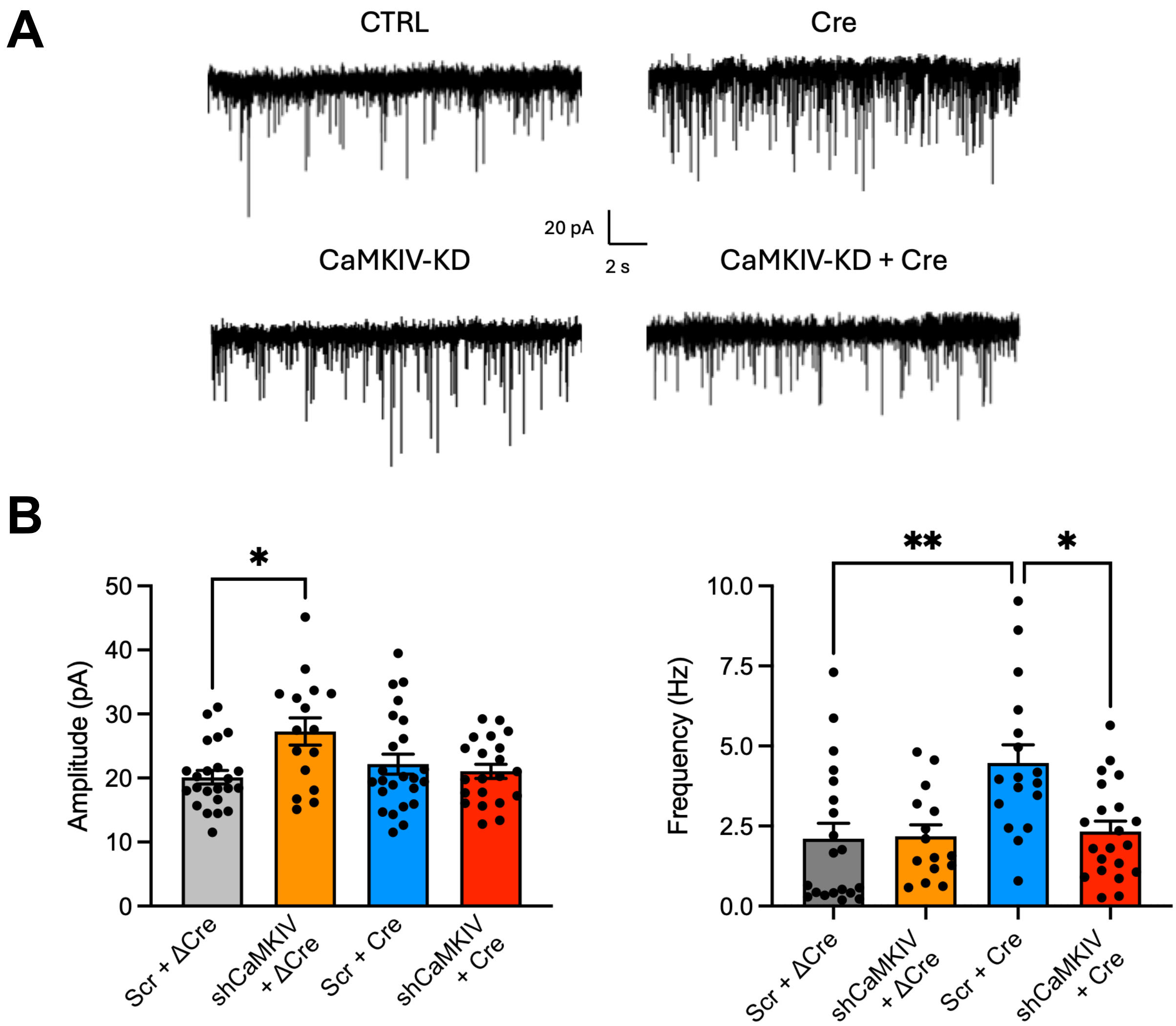

Figure S19. mEPSCs in neurons after the conditional knockout of REST and/or CaMKIV knockdown. A. Representative traces of mEPSCs recorded in primary cortical neurons that were knocked out for REST (*Rest*^{Cre}) or kept WT (*Rest*$^{\Delta Cre}$) and co-transduced with either *CaMKIV*^{Scr} or *CaMKIV*^{Sh} expressing lentiviruses. **B.** Mean (± sem with individual experimental points) of the mEPSC amplitude (*left*) and frequency (*right*) in *Rest*$^{\Delta Cre}$ + *CaMKIV*^{Scr} (n=20), *Rest*$^{\Delta Cre}$ + *CaMKIV*^{Sh} (n=15), *Rest*^{Cre} + *CaMKIV*^{Scr} (n=17), *Rest*^{Cre} + *CaMKIV*^{Sh} (n=21) neurons from n=3 independent neuronal preparations. Amplitude: *p=0.0172, *CaMKIV*^{Scr} *vs CaMKIV*^{Sh} with *Rest*$^{\Delta Cre}$; Frequency: **p=0.071, *Rest*$^{\Delta Cre}$ *vs Rest*^{Cre} with *CaMKIV*^{Scr}; *p=0.0126, *CaMKIV*^{Scr} *vs CaMKIV*^{Sh} with *Rest*^{Cre}; 2-way ANOVA/Tukey's tests.

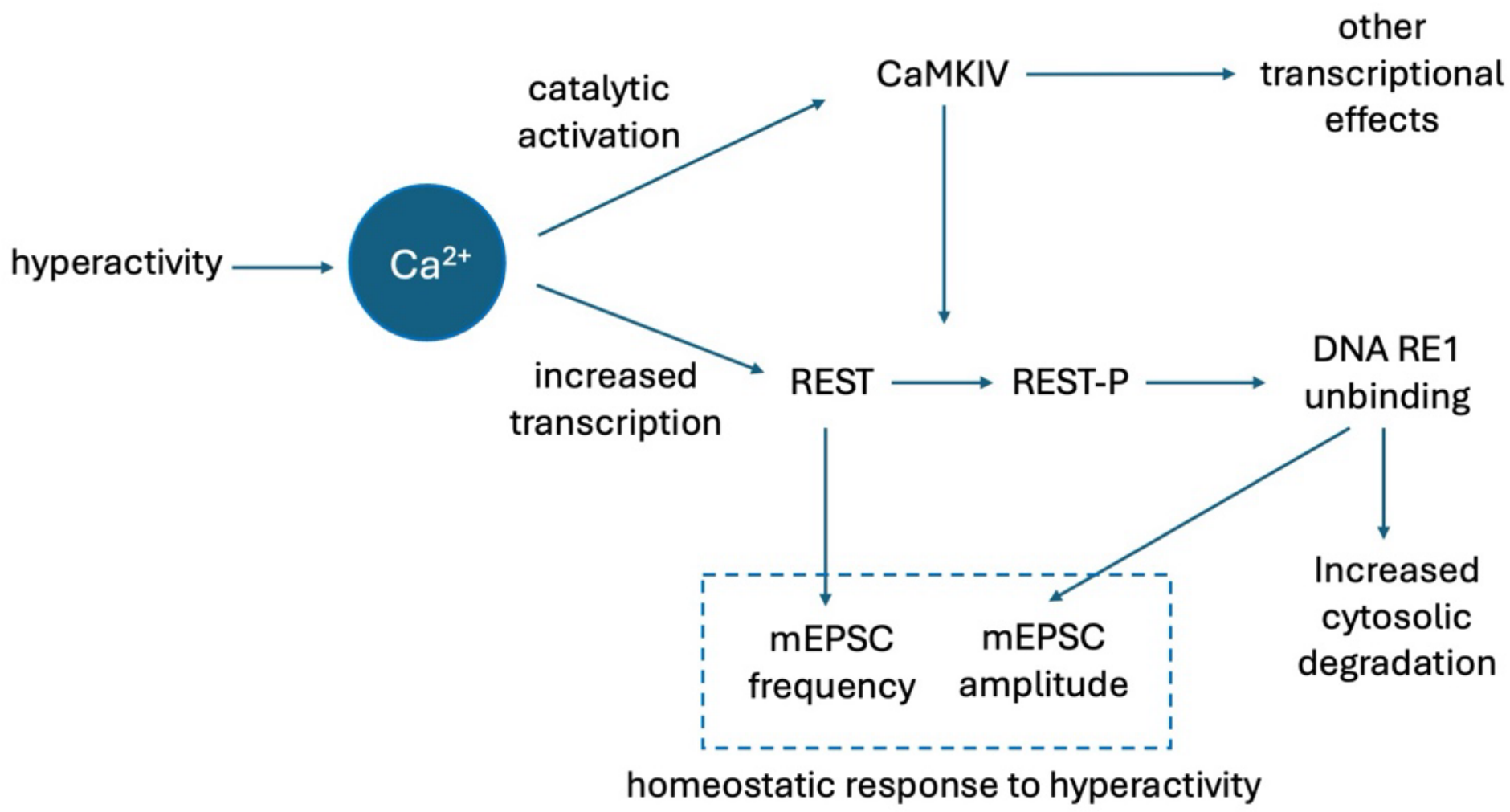

Figure S20. Proposed model for the interaction between CaMKIV and REST in the synaptic responses to chronic hyperactivity. Calcium entry during hyperactivity activates both REST expression and CaMKIV, which translocates to the nucleus and phosphorylates transcription factors, including REST. By acting at the transcriptional level in the nucleus, REST homeostatically downregulates excitatory synaptic transmission by decreasing mEPSC frequency, while activation of CaMKIV results in a homeostatic downscaling of mEPSC amplitude. Phosphorylated REST at S^{322} has reduced affinity for the RE1 sequences of its target genes, and, upon unbinding, shuttles back to the cytosol, where it is eventually targeted for degradation. The effect of CaMKIV on mEPSC amplitude, which depends on REST expression, is likely attributable to the loss of the transcriptional effects of REST upon CaMKIV-phosphorylation.

REFERENCES

1. A. W. Bruce, I. J. Donaldson, I. C. Wood, S. A. Yerbury, M. I. Sadowski, M. Chapman, B. Göttgens, N. J. Buckley, Genome-wide analysis of repressor element 1 silencing transcription factor/neuron-restrictive silencing factor (REST/NRSF) target genes. *Proc. Natl. Acad. Sci. U.S.A.* **101**,10458–10463 (2004).

2. N. Ballas, G. Mandel, The many faces of REST oversee epigenetic programming of neuronal genes. *Curr. Opin. Neurobiol.* **15**, 500–506 (2005).

3. N. Mori, C. Schoenherr, D. J. Vandenbergh, D. J. Anderson, A common silencer element in the SCG10 and type II Na^+ channel genes binds a factor present in nonneuronal cells but not in neuronal cells. *Neuron* **9,** 45–54 (1992).

4. S. D. Kraner, J. A. Chong, H. J. Tsay, G. Mandel, Silencing the type II sodium channel gene: A model for neural-specific gene regulation. *Neuron* **9**, 37–44 (1992).

5. N. Ballas, C. Grunseich, D. D. Lu, J. C. Speh, G. Mandel, REST and its corepressors mediate plasticity of neuronal gene chromatin throughout neurogenesis. *Cell* **121,** 645–657 (2005).

6. M. Yeo, K. Berglund, G. Augustine, W. Liedtke, Novel repression of Kcc2 transcription by REST-RE-1 controls developmental switch in neuronal chloride. *J. Neurosci.* **29**, 14652–14662 (2009).

7. A. Rodenas-Ruano, A. E. Chávez, M. J. Cossio, P. E. Castill, R. S. Zukin, REST-dependent epigenetic remodeling promotes the developmental switch in synaptic NMDA receptors. *Nat. Neurosci.* **15**, 1382–1390 (2012).

8. H. Aoki, A. Hara, T. Era, T. Kunisada, Y. Yamada, Genetic ablation of Rest leads to in vitro-specific derepression of neuronal genes during neurogenesis. *Development* **139**, 667–677 (2012).

9. P. Baldelli, J. Meldolesi, The transcription repressor REST in adult neurons: Physiology, pathology, and diseases. *eNeuro* **2**, ENEURO.0010-15.2015 (2015).

10. C. Michetti, F. Benfenati, Homeostatic regulation of brain activity: From endogenous mechanisms to homeostatic nanomachines. *Am. J. Physiol. Cell Physiol.* **327**, C1384–C1399 (2024).

11. D. Pozzi, G. Lignani, E. Ferrea, A. Contestabile, F. Paonessa, R. D'Alessandro, P. Lippiello, D. Boido, A. Fassio, J. Meldolesi, F. Valtorta, F. Benfenati, P. Baldelli, REST/NRSF-mediated intrinsic homeostasis protects neuronal networks from hyperexcitability. *EMBO J.* **32**, 2994–3007 (2013).

12. F. Pecoraro-Bisogni, G. Lignani, A. Contestabile, E. Castroflorio, D. Pozzi, A. Rocchi, C. Prestigio, M. Orlando, P. Valente, M. Massacesi, F. Benfenati, P. Baldelli, REST-dependent presynaptic homeostasis induced by chronic neuronal hyperactivity. *Mol. Neurobiol.* **55**, 4959–4972 (2018).

13. C. Prestigio, D. Ferrante, A. Marte, A. Romei, G. Lignani, F. Onofri, P. Valente, F. Benfenati, P. Baldelli, REST/NRSF drives homeostatic plasticity of inhibitory synapses in a target-dependent fashion. *eLife* **10,** e69058 (2021).

14. A. Singh-Taylor, J. Molet, S. Jiang, A. Korosi, J. L. Bolton, Y. Noam, K. Simeone, J. Cope, Y. Chen, A. Mortazavi, T. Z. Baram, NRSF-dependent epigenetic mechanisms contribute to programming of stress-sensitive neurons by neonatal experience, promoting resilience. *Mol. Psychiatry* **23**, 648–657 (2018).

15. M. Mampay, G. K. Sheridan, REST: An epigenetic regulator of neuronal stress responses in the young and ageing brain. *Front. Neuroendocrinol.* **53**, 100744 (2019).

16. J. L. Bolton, A. Schulmann, M. M. Garcia-Curran, L. Regev, Y. Chen, N. Kamei, M. Shao, A. Singh-Taylor, S. Jiang, Y. Noam, J. Molet, A. Mortazavi, T. Z. Baram, Unexpected transcriptional programs contribute to hippocampal memory deficits and neuronal stunting after early-life adversity. *Cell Rep.* **33**, 108511 (2020).

17. T. Lu, L. Aron, J. Zullo, Y. Pan, H. Kim, Y. Chen, T. H. Yang, H. M. Kim, D. Drake, X. S. Liu, D. A. Bennett, M. P. Colaiácovo, B. A. Yankner, REST and stress resistance in ageing and Alzheimer's disease. *Nature* **507,** 448–454 (2014).

18. J. M. Zullo, D. Drake, L. Aron, P. O'Hern, S. C. Dhamne, N. Davidsohn, C. A. Mao, W. H. Klein, A. Rotenberg, D. A. Bennett, G. M. Church, M. P. Colaiácovo, B. A. Yankner, Regulation of lifespan by neural excitation and REST. *Nature* **574**, 359–364 (2019).

19. A. Rocchi, E. Carminati, A. De Fusco, J. A. Kowalska, T. Floss, F. Benfenati, REST/NRSF deficiency impairs autophagy and leads to cellular senescence in neurons. *Aging Cell* **20,** e13471 (2021).

20. E. Centonze, A. Marte, M. Albini, A. Rocchi, F. Cesca, M. Chiacchiaretta, T. Floss, P. Baldelli, S. Ferroni, F. Benfenati, P. Valente, Neuron-restrictive silencer factor/repressor element 1-silencing transcription factor (NRSF/REST) controls spatial K^+ buffering in primary cortical astrocytes. *J. Neurochem.* **165**, 701–721 (2023).

21. N. J. Buckley, R. Johnson, C. Zuccato, A. Bithell, E. Cattaneo, The role of REST in transcriptional and epigenetic dysregulation in Huntington's disease. *Neurobiol. Dis.* **39**, 28–39 (2010).

22. S. Mcclelland, C. Flynn, C. Dubé, C. Richichi, Q. Zha, A. Ghestem, M. Esclapez, C. Bernard, T. Z. Baram, Neuron-restrictive silencer factor-mediated hyperpolarization-activated cyclic nucleotide gated channelopathy in experimental temporal lobe epilepsy. *Ann. Neurol.* **70**, 454–465 (2011).

23. S. Mcclelland, G. P. Brennan, C. Dubé, S. Rajpara, S. Iyer, C. Richichi, C. Bernard, T. Z. Baram, The transcription factor NRSF contributes to epileptogenesis by selective repression of a subset of target genes. *eLife* **3**, e01267 (2014).

24. A. M. Hall, N. Kamei, M. Shao, H.-S. Mun, K. Chen, Y. Chen, T. Z. Baram, Inhibition of Neuron-Restrictive Silencing Factor (REST/NRSF) chromatin binding attenuates epileptogenesise. *Neuro* **11**, ENEURO.0006-24.2024 (2024).

25. E. Carminati, F. Buffolo, A. Rocchi, C. Michetti, F. Cesca, F. Benfenati, Mild inactivation of RE-1 silencing transcription factor (REST) reduces susceptibility to kainic acid-induced seizures. *Front. Cell. Neurosci.* **13**, 580 (2020).

26. F. Buffolo, V. Petrosino, M. Albini, M. Moschetta, F. Carlini, T. Floss, N. K. De Rosbo, F. Cesca, A. Rocchi, A. Uccelli, F. Benfenati, Neuroinflammation induces synaptic scaling through IL-1β-mediated activation of the transcriptional repressor REST/NRSF. *Cell Death Dis.* **12**, 180 (2021).

27. G. Natali, C. Michetti, A. Krawczun-Rygmaczewska, T. Floss, F. Cesca, F. Benfenati, Conditional knockout of REST/NRSF in excitatory neurons reduces seizure susceptibility to chemical kindling. *Front. Cell. Neurosci.* **17**, 1267609 (2023).

28. J.-Y. Hwang, R. S. Zukin, REST, a master transcriptional regulator in neurodegenerative disease. *Curr. Opin. Neurobiol.* **48**, 193–200 (2018).

29. M. Shimojo, L. B. Hersh, Characterization of the REST/NRSF-interacting LIM domain protein (RILP): Localization and interaction with REST/NRSF. *J. Neurochem.* **96**, 1130–1138 (2006).

30. F. Paonessa, S. Criscuolo, S. Sacchetti, D. Amoroso, H. Scarongella, F. Pecoraro Bisogni, E. Carminati, G. Pruzzo, L. Maragliano, F. Cesca, F. Benfenati, Regulation of neural gene transcription by optogenetic inhibition of the RE1-silencing transcription factor. *Proc. Natl. Acad. Sci. U.S.A.* **113**, E91–100 (2015).

31. K. Palm, N. Belluardo, M. Metsis, T. Õ. Timmusk, Neuronal expression of zinc finger transcription factor REST/NRSF/XBR gene. *J. Neurosci.* **18**, 1280–1296 (1998).

32. S. Rockowitz, W.-H. Lien, E. Pedrosa, G. Wei, M. Lin, K. Zhao, H. M. Lachman, E. Fuchs, D. Zheng, Comparison of REST cistromes across human cell types reveals common and context-specific functions. *PLoS Comput. Biol.* **10**, e1003671 (2014).

33. C. Brenner, R. Deplus, C. Didelot, A. Loriot, E. Viré, C. De Smet, A. Gutierrez, D. Danovi, D. Bernard, T. Boon, P. Giuseppe Pelicci, B. Amati, T. Kouzarides, Y. De Launoit, L. Di Croce, F.

Fuks, Myc represses transcription through recruitment of DNA methyltransferase corepressor. *EMBO J.* **24**, 336–346 (2005).

34. W. J. Friesen, M. K. Darby, Phage display of RNA binding zinc fingers from transcription factor IIIA. *J. Mol. Biol.* **272**, 10994–10997 (1997).

35. A. Klug, The discovery of zinc fingers and their development for practical applications in gene regulation and genome manipulation. *Q. Rev. Biophys.* **43**, 1–21 (2010).

36. T. F. Westbrook, G. Hu, X. L. Ang, P. Mulligan, N. N. Pavlova, A. Liang, Y. Leng, R. Maehr, Y. Shi, J. W. Harper, S. J. Elledge, SCFβ-TRCP controls oncogenic transformation and neural differentiation through REST degradation. *Nature* **452**, 370–374 (2008).

37. D. Guardavaccaro, D. Frescas, N. V. Dorrello, A. Peschiaroli, A. S. Multani, T. Cardozo, A. Lasorella, A. Iavarone, S. Chang, E. Hernando, M. Pagano, Control of chromosome stability by the β-TrCP–REST–Mad2 axis. *Nature* **452**, 365–369 (2008).

38. N. Kaneko, J.-Y. Hwang, M. Gertner, F. Pontarelli, R. S. Zukin, Casein kinase 1 suppresses activation of REST in insulted hippocampal neurons and halts ischemia-induced neuronal death. *J. Neurosci.* **34**, 6030–6039 (2014).

39. E. Nesti, G. M. Corson, M. Mccleskey, J. A. Oyer, G. Mandel, C-terminal domain small phosphatase 1 and MAP kinase reciprocally control REST stability and neuronal differentiation. *Proc. Natl. Acad. Sci. U.S.A.* **111**, E3929–36 (2014).

40. G. G. Turrigiano, The self-tuning neuron: Synaptic scaling of excitatory synapses. *Cell* **135**, 422–435 (2008).

41. G. Turrigiano, Too many cooks? Intrinsic and synaptic homeostatic mechanisms in cortical circuit refinement. *Annu. Rev. Neurosci.* **34**, 89–103 (2011).

42. N. Ageta-Ishihara, S. Takemoto-Kimura, M. Nonaka, A. Adachi-Morishima, K. Suzuki, S. Kamijo, H. Fujii, T. Mano, F. Blaeser, T. A. Chatila, H. Mizuno, T. Hirano, Y. Tagawa, H.

Okuno, H. Bito, Control of cortical axon elongation by a GABA-driven Ca^{2+}/calmodulin-dependent protein kinase cascade. *J. Neurosci.* **29**, 13720–13729 (2009).

43. D. C. Bersten, J. A. Wright, P. J. Mccarthy, M. L. Whitelaw, Regulation of the neuronal transcription factor NPAS4 by REST and microRNAs. *Biochim. Biophys. Acta* **1839**, 13–24 (2013).

44. H. Xu, L. Yong, X. Gao, Y. Chen, Y. Wang, F. Wang, X. Hou, CaMK4: Structure, physiological functions, and therapeutic potential. *Biochem. Pharmacol.* **224**, 116204 (2024).

45. J. Kaiser, A. Risteska, A. G. Muller, H. Sun, B. Lei, K. Nay, A. R. Means, M. A. Cousin, D. H. Drewry, J. S. Oakhill, B. E. Kemp, A. J. Hannan, M. Berk, M. A. Febbraio, A. L. Gundlach, E. L. Hill-Yardin, J. W. Scott, Convergence on CaMK4: A key modulator of autism-associated signaling pathways in neurons. *Biol. Psychiatry* **97**, 439–449 (2025).

46. S. M. Lemrow, K. A. Anderson, J. D. Joseph, T. J. Ribar, P. K. Noeldner, A. R. Means, Catalytic activity is required for calcium/calmodulin-dependent protein kinase IV to enter the nucleus. *J. Biol. Chem.* **279**, 11664–11671 (2004).

47. R. P. Matthews, C. R. Guthrie, L. M. Wailes, X. Zhao, A. R. Means, G. S. Mcknight, Calcium/calmodulin-dependent protein kinase types II and IV differentially regulate CREB-dependent gene expression. *Mol. Cell. Biol.* **14**, 6107–6116 (1994).

48. R. R. White, Y.-G. Kwon, M. Taing, D. S. Lawrence, A. M. Edelman, Definition of optimal substrate recognition motifs of Ca^{2+}-calmodulin-dependent protein kinases IV and II reveals shared and distinctive features *J. Biol. Chem.* **273**, 3166–3172 (1998).

49. D. Jantz, J. M. Berg, Reduction in DNA-binding affinity of Cys_2His^2 zinc finger proteins by linker phosphorylation. *Proc. Natl. Acad. Sci. U.S.A.* **101**, 7589–7593 (2004).

50. A. Joseph, G. G. Turrigiano, All for one but not one for all: Excitatory synaptic scaling and intrinsic excitability are coregulated by CaMKIV, whereas inhibitory synaptic scaling is under independent control. *J. Neurosci.* **37**, 6778–6785 (2017).

51. Y. Qiu, N. O'Neill, B. Maffei, C. Zourray, A. Almacellas-Barbanoj, J. C. Carpenter, S. P. Jones, M. Leite, T. J. Turner, F. C. Moreira, On-demand cell-autonomous gene therapy for brain circuit disorders. *Science* **378**, 523–532 (2022).

52. J. C. Lee, Y. G. Kwon, D. S. Lawrence, A. M. Edelman, A requirement of hydrophobic and basic amino acid residues for substrate recognition by Ca2+/calmodulin-dependent protein kinase Ia. *Proc. Natl. Acad. Sci. U.S.A.* **91**, 6413–6417 (1994).

53. S. Dale, W. A. Wilson, A. M. Edelman, D. G. Hardie, Similar substrate recognition motifs for mammalian AMP-activated protein kinase, higher plant HMG-CoA reductase kinase-A, yeast SNF1, and mammalian calmodulin-dependent protein kinase I. *FEBS Lett.* **361**, 191–195 (1995).

54. S. Dovat, T. Ronni, D. Russell, R. Ferrini, B. S. Cobb, S. T. Smale, A common mechanism for mitotic inactivation of C2H2 zinc finger DNA-binding domains. *Genes Dev.* **16**, 2985–2990 (2002).

55. R. Rizkallah, K. E. Alexander, M. M. Hurt, Global mitotic phosphorylation of C2H2zinc finger protein linker peptides. *Cell Cycle* **10**, 3327–3336 (2011).

56. R. Conesa-Bakkali, M. Morillo-Huesca, J. Martínez-Fábregas, Non-canonical, extralysosomal activities of lysosomal peptidases in physiological and pathological conditions: New clinical opportunities for cancer therapy. *Cells* **14**, 68 (2025).

57. M. C. Yoon, M. P. Christy, V. V. Phan, W. H. Gerwick, G. Hook, A. J. O'Donoghue, V. Hook, Molecular features of CA-074 pH-dependent inhibition of cathepsin B. *Biochemistry* **61**, 228–238 (2022).

58. A. M. Cuervo, J. F. Dice, E. Knecht, A population of rat liver lysosomes responsible for the selective uptake and degradation of cytosolic proteins. *J. Biol. Chem.* **272**, 5606–5615 (1997).

59. S. J. Myers, J. Peters, Y. Huang, M. B. Comer, F. Barthel, R. Dingledine, Transcriptional regulation of the GluR2 gene: Neural-specific expression, multiple promoters, and regulatory elements. *J. Neurosci.* **18**, 6723–6739 (1998).

60. A. Calderone, T. Jover, K. Noh, H. Tanaka, H. Yokota, Y. Lin, S. Y. Grooms, R. Regis, M. V. L. Bennett, R. S. Zukin, Ischemic insults derepress the gene silencer REST in neurons destined to die. *J. Neurosci.* **23**, 2112–2121 (2003).

61. K.-M. Noh, J.-Y. Hwang, A. Follenzi, R. Athanasiadou, T. Miyawaki, J. M. Greally, M. V. L. Bennett, R. S. Zukin, Repressor element-1 silencing transcription factor (REST)-dependent epigenetic remodeling is critical to ischemia-induced neuronal death. *Proc. Natl. Acad. Sci. U.S.A.* **109** E962–E971 (2012).

62. P. Kallunki, G. M. Edelman, F. S. Jones, The neural restrictive silencer element can act as both a repressor and enhancer of L1 cell adhesion molecule gene expression during postnatal development. *Proc. Natl. Acad. Sci. U.S.A.* **95**, 3233–3238 (1998).

63. A. Perera, D. Eisen, M. Wagner, S. K. Laube, A. F. Künzel, S. Koch, J. Steinbacher, E. Schulze, V. Splith, N. Mittermeier, TET3 is recruited by REST for context-specific hydroxymethylation and induction of gene expression. *Cell Rep.* **11**, 283–294 (2015).

64. E. Pajarillo, A. Digman, I. Nyarko-Danquah, D.-S. Son, K. F. A. Soliman, M. Aschner, E. Lee, Astrocytic transcription factor REST upregulates glutamate transporter EAAT2, protecting dopaminergic neurons from manganese-induced excitotoxicity. *J. Biol. Chem.* **297**, 101372 (2021).

65. T. Nechiporuk, J. McGann, K. Mullendorff, J. Hsieh, W. Wurst, T. Floss, G. Mandel, The REST remodeling complex protects genomic integrity during embryonic neurogenesis. *eLife* **5**, e09584 (2016).

66. P. Baldelli, A. Fassio, F. Valtorta, F. Benfenati, Lack of synapsin I reduces the readily releasable pool of synaptic vesicles at central inhibitory synapses. *J. Neurosci.* **27**, 13520–13531 (2007).

67. M. Chiappalone, S. Casagrande, M. Tedesco, F. Valtorta, P. Baldelli, S. Martinoia, F. Benfenati, Opposite changes in glutamatergic and GABAergic transmission underlie the diffuse hyperexcitability of synapsin I-deficient cortical networks. *Cereb. Cortex* **19**, 1422–1439 (2009).

68. P. Valente, E. Castroflorio, P. Rossi, M. Fadda, B. Sterlini, R. I. Cervigni, C. Prestigio, S. Giovedì, F. Onofri, E. Mura, F. C. Guarnieri, A. Marte, M. Orlando, F. Zara, A. Fassio, F. Valtorta, P. Baldelli, A. Corradi, F. Benfenati, PRRT2 is a key component of the Ca^{2+}-dependent neurotransmitter release machinery. *Cell Rep.* **15**, 117–131 (2016).

69. P. Sun, H. Enslen, P. S. Myung, R. A. Maurer, Differential activation of CREB by Ca^{2+}/calmodulin-dependent protein kinases type II and type IV involves phosphorylation of a site that negatively regulates activity. *Genes Dev.* **8**, 2527–2539 (1994).

70. V. Castagnola, V. Tomati, L. Boselli, C. Braccia, S. Decherchi, P. P. Pompa, N. Pedemonte, F. Benfenati, A. Armirotti, Sources of biases in the in vitro testing of nanomaterials: The role of the biomolecular corona. *Nanoscale Horiz.* **9**, 799–816 (2024).

71. N. Liessi, L. Maragliano, V. Castagnola, M. Bramini, F. Benfenati, A. Armirotti, Isobaric labeling proteomics allows a high-throughput investigation of protein corona orientation. *Anal. Chem.* **93**, 784–791 (2021).

72. C. L. Fisher, G. K. Pei. Modification of a PCR-based site-directed mutagenesis method. *Biotechniques* **23**, 570–574 (1997).

73. J. Vandesompele, K. De Preter, F. Pattyn, B. Poppe, N. Van Roy, A. De Paepe, F. Speleman, Accurate normalization of real-time quantitative RT-PCR data by geometric averaging of multiple internal control genes. *Genome Biol.* **3**, RESEARCH0034 (2002).

74. S. Bolte, F. P. Cordelières, A guided tour into subcellular colocalization analysis in light microscopy. *J. Microsc.* **224**, 213–232 (2006).

75. M. De Palma, L. Naldini, Transduction of a gene expression cassette using advanced generation lentiviral vectors. *Methods Enzymol.* **346**, 514–529 (2002).

76. J. Lee, X. Cheng, J. M. Swails, M. S. Yeom, P. K. Eastman, J. A. Lemkul, S. Wei, J. Buckner, J. C. Jeong, Y. Qi, S. Jo, V. S. Pande, D. A. Case, C. L. Brooks III, A. D. MacKerell Jr, J. B. Klauda, W. Im, Charmm-gui input generator for namd, gromacs, amber, openmm, and

charmm/openmm simulations using the charmm36 additive force field. *J. Chem. Theory Comput.* **12**, 405–413 (2016).

77. J. Lee, D. S. Patel, J. Ståhle, S. J. Park, N. R. Kern, S. Kim, J. Lee, X. Cheng, M. A. Valvano, O. Holst, Y. A. Knirel, Y. Qi, S. Jo, J. B. Klauda, G. Widmalm, W. Im, Charmm-gui membrane builder for complex biological membrane simulations with glycolipids and lipoglycans. *J. Chem. Theory Comput.* **15**, 775–786 (2019).

78. W. L. Jorgensen, J. Chandrasekhar, J. D. Madura, R. W. Impey, M. L. Klein, Comparison of simple potential functions for simulating liquid water. *J. Chem. Phys.* **79**, 926–935 (1983).

79. J. C. Phillips, D. J. Hardy, J. D. C. Maia, J. E. Stone, J. V. Ribeiro, R. C. Bernardi, R. Buch, G. Fiorin, J. Hénin, W. Jiang, R. McGreevy, M. C. R. Melo, B. K. Radak, R. D. Skeel, A. Singharoy, Y. Wang, B. Roux, A. Aksimentiev, Z. Luthey-Schulten, L. V. Kalé, K. Schulten, C. Chipot, E. Tajkhorshid, Scalable molecular dynamics on CPU and GPU architectures with NAMD. *J. Chem. Phys.* **153**, 044130 (2020).

80. J. A. Maier, C. Martinez, K. Kasavajhala, L. Wickstrom, K. E. Hauser, C. Simmerling, ff14SB: Improving the accuracy of protein side chain and backbone parameters from ff99SB. *J. Chem. Theory Comput.* **11**, 3696–3713 (2015).

81. I. Ivani, P. D. Dans, A. Noy, A. Pérez, I. Faustino, A. Hospital, J. Walther, P. Andrio, R. Goñi, A. Balaceanu, G. Portella, F. Battistini, J. L. Gelpí, C. González, M. Vendruscolo, C. A. Laughton, S. A. Harris, D. A. Case, M. Orozco, Parmbsc1: A refined force field for DNA simulations. *Nat. Methods* **13**, 55–58 (2016).

82. S. W. de Leeuw, J. W. Perram, E. R. Smith, Simulation of electrostatic systems in periodic boundary conditions. I. Lattice sums and dielectric constants. *Proc. R. Soc. A* **373**, 27–56 (1980).

83. T. Darden, D. York, L. Pedersen, Particle mesh Ewald: An $N \cdot \log(N)$ method for Ewald sums in large systems. *J. Chem. Phys.* **98**, 10089–10092 (1993).

84. J. P. Ryckaert, G. Ciccotti, H. J. C. Berendsen, Numerical integration of the Cartesian equations of motion of a system with constraints: Molecular dynamics of n-alkanes. *J. Comput. Phys.* **23**, 327–341 (1977).

85. S. E. Feller, Y. Zhang, R. W. Pasto, B. R. Brooks, Constant pressure molecular dynamics simulation: The Langevin piston method. *J. Chem. Phys.* **103**, 4613–4621 (1995).

86. B. Leimkuhler, E. Noorizadeh, F. Theil, A gentle stochastic thermostat for molecular dynamics. *J. Stat. Phys.* **135**, 261–277 (2009).

87. T. Tubiana, J.-C. Carvaillo, Y. Boulard, S. Bressanelli, TTClust: A versatile molecular simulation trajectory clustering program with graphical summaries. *J. Chem. Inf. Model.* **58**, 2178–2182 (2018).

88. F. Murtagh, P. Legendre, Ward's hierarchical agglomerative clustering method: Which algorithms implement ward's criterion? *J. Classif.* **31**, 274–295 (2014).

89. Schrödinger LLC, The PyMOL Molecular Graphics System, version 2.0 (Schrödinger LLC, New York, NY, 2002).

90. W. Zheng, Q. Wuyun, P. L. Freddolino, Y. Zhang, Integrating deep learning, threading alignments, and a MULTI-MSA strategy for high-quality protein monomer and complex structure prediction in CASP15. *Proteins* **91**, 1684–1703 (2023).

91. J. Yang, Y. Zhang, Protein structure and function prediction using I-TASSER. *Curr. Protoc. Bioinformatics* **52**, 5.8.1–5.8.15 (2015).

92. J. Jumper, R. Evans, A. Pritzel, T. Green, M. Figurnov, O. Ronneberger, K. Tunyasuvunakool, R. Bates, A. Žídek, A. Potapenko, A. Bridgland, C. Meyer, S. A. A. Kohl, A. J. Ballard, A. Cowie, B. Romera-Paredes, S. Nikolov, R. Jain, J. Adler, T. Back, S. Petersen, D. Reiman, E. Clancy, M. Zielinski, M. Steinegger, M. Pacholska, T. Berghammer, S. Bodenstein, D. Silver, O. Vinyals, A. W. Senior, K. Kavukcuoglu, P. Kohli, D. Hassabis, Highly accurate protein structure prediction with AlphaFold. *Nature* **596**, 583–589 (2021).

93. H. Park, G. R. Lee, D. E. Kim, I. Anishchenko, Q. Cong, D. Baker, High-accuracy refinement using Rosetta in CASP13. *Proteins* **87**, 1276–1282 (2019).

94. Y. Yan, H. Tao, J. He, S. Y. Huang, The HDOCK server for integrated protein–protein docking. *Nat. Protoc.* **15**, 1829–1852 (2020).

95. H. M. Berman, J. Westbrook, Z. Feng, G. Gilliland, T. N. Bhat, H. Weissig, I. N. Shindyalov, P. E. Bourne. The Protein Data Bank. *Nucleic Acids Res.* **28**, 235–242 (2000).

96. G. M. Boratyn, A. A. Schäffer, R. Agarwala, S. F. Altschul, D. J. Lipman, T. L. Madden, Domain enhanced lookup time accelerated BLAST. *Biol. Direct* **7**, 12 (2012).

97. S. Henikoff, J. G. Henikoff, Amino acid substitution matrices from protein blocks. *Proc. Natl. Acad. Sci.* **89**, 10915–10919 (1992).

98. S. L. Dürr, A. Levy, U. Rothlisberger, Metal3D: A general deep learning framework for accurate metal ion location prediction in proteins. *Nat. Commun.* **14**, 2713 (2023).

99. E. Neria, S. Fischer, M. Karplus, Simulation of activation free energies in molecular systems. *J. Chem. Phys.* **105**, 1902–1921 (1996).

100. P. Mark, L. Nilsson, Structure and dynamics of the TIP3P, SPC, and SPC/E water models at 298 K. *J. Phys. Chem.* **105**, 9954–9960 (2001).

101. S. Miyamoto, P. A. Kollman, SETTLE: An analytical version of the SHAKE and RATTLE algorithm for rigid water models. *J. Comput. Chem.* **13**, 952–962 (1992).

102. G. Fiorin, M. L. Klein, J. Hénin, Using collective variables to drive molecular dynamics simulations. *Mol. Phys.* **111**, 3345–3362 (2013).
